# Multi-Port Vacuum Assembly Device Designed for Simplified Point-of-Need Chemical and Biological Analyses Using Mass Spectrometry

**DOI:** 10.1021/acs.analchem.6c02029

**Published:** 2026-08-22

**Authors:** Sarah Trimpin, Ellen D. Inutan, Hope Coffinberger, Frank S. Yenchick, Dushan Kovacevic, Sullivan Stimac, John W. Tomsho, Ken Mackie, Milan Pophristic, Charles N. McEwen

**Affiliations:** † Department of Chemistry, 2954 Wayne State University, Detroit 48202, Michigan, United States; ‡ Research & Development, MSTM, Newark 19711, Delaware, United States; § Department of Chemistry, 69330 Mindanao State University - Iligan Institute of Technology, Iligan City 9200, Philippines; ∥ Department of Chemistry & Biochemistry, 6557 Saint Joseph’s University, Philadelphia 19104, Pennsylvania, United States; ⊥ Singidunum, LLC, Farmington Hills 48331, Michigan, United States; # Department of Psychological and Brain Sciences, 1772 Indiana University, Bloomington 47405, Indiana, United States

## Abstract

A persistent challenge in analytical chemistry is the effective analysis of all components in a sample mixture. For mass spectrometric analysis, this begins with ionization. Over the past several decades, extensive research has been conducted to enhance sample cleanliness and optimize various ionization techniques. The proof-of-feasibility analytical technology presented herein pertains to a vacuum chamber assembly equipped with easily accessible multiple ports specifically designed for different mass spectrometry applications. These ports, *inter alia*, facilitate atmospheric-pressure ionization, such as ESI and APCI, as well as newer methods, including *inlet* ionization, *vacuum* ionization, and ambient ionization, thus enabling the ready selection of the most suitable ionization/analysis methods on a single mass spectrometer without requiring instrument venting. This capability is enabled by a unique multiport vacuum interface assembly that accommodates a diverse range of functions to enhance ionization and facilitate the transfer of analyte ions and charged particles into a mass analyzer. The ports may serve auxiliary functions that expand and improve the available ionization methodologies or provide features that are not typically accessible. This technology demonstrates the potential to maximize the information content extractable directly from a sample in a timely and cost-effective manner, thus promising extensive applications across various fields, including medicine and national security.

## Graphical abstract

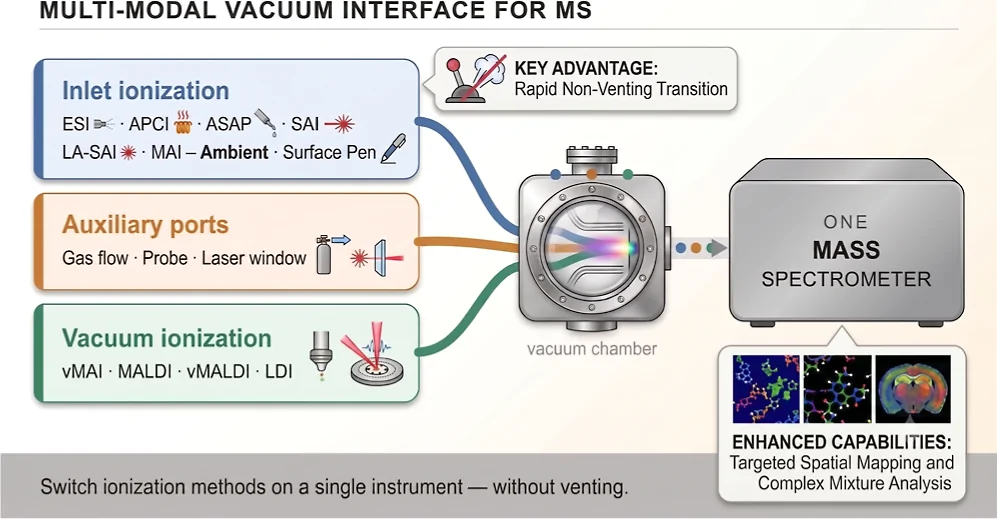

## Introduction

Mass spectrometry (MS) is a powerful analytical technique capable of providing detailed compositional and quantitative information from minimal sample material. The technology addresses diverse analytical challenges across biological, inorganic, and synthetic systems, often in combination with liquid or gas chromatography and other sample cleanup and preparation methods. Applications range from bacterial differentiation and environmental threat detection to material quality assessment, demonstrating the broad utility of MS across scientific disciplines.

Despite its analytical power, MS presents significant barriers to widespread adoption. The technology requires substantial upfront capital investment and ongoing operational costs, including maintenance and specialized training. Different analytical objectives often necessitate distinct ionization sources and methodologies, and multiple specialized instrument systems. This complexity typically confines MS to centralized laboratory settings staffed by expert users, limiting accessibility for researchers working across diverse areas such as intact protein analysis or forensic applications.

The challenge of selecting appropriate ionization methods and instrument configurations represents a critical bottleneck in MS accessibility. Addressing this complexity through simplified, more intuitive MS platforms represents an emerging research frontier with considerable promise. By reducing technical barriers and expertise requirements, next-generation MS systems could democratize access to rapid, accurate analytical capabilities, enabling novice users to fully leverage the technology’s potential across a broader range of scientific and practical applications.

In MS analysis, a mass analyzer separates gaseous ions based on their mass-to-charge (*m*/*z*) ratios. An ionization process is required to generate the gaseous, preferably intact, molecular ions that are transferred through the analyzer, thereby separating them and enabling their individual detection and mass determination. Separation and detection occur under subatmospheric pressure (subAP) conditions, typically in high- or ultrahigh-vacuum. Therefore, a crucial aspect of MS measurements is converting compounds into gaseous molecular ions,^1^ and, for most ionization methods, transferring from atmospheric pressure (AP) to vacuum. Because of their importance, ionization and ion-transmission processes have been modeled and optimized over several decades to increase the number of analyte ions generated and reaching the analyzer and detector.^2–6^

Ionization sources, such as electrospray ionization (ESI)^7^ and matrix-assisted laser desorption/ionization (MALDI),^8^ use matrices, either liquid or solid, to facilitate ionization. In contrast, more recent methods, such as the atmospheric solids analysis probe (ASAP)^9,10^ and direct analysis in real time (DART)^11,12^ involve minimal sample preparation but are limited to volatile compounds with relatively low molecular weights. They have become popular due to their simplicity as direct analytical methods that bypass extensive separation and cleanup techniques prior to mass measurements.

Voltages applied to lens elements are frequently used to guide ions toward the mass analyzer and to eliminate neutral particles, thereby helping maintain instrument cleanliness. Lens elements include ion funnels, S-lenses, and multipoles. Aggressive methods are used to remove matrix molecules from charged particles containing analyte before they reach these lens elements. Charged particles can carry a substantial amount of analyte, which then goes unanalyzed if the matrix is not removed before mass separation. Ionization sources that ionize at AP may employ heated gases to desolvate charged particles and to aid the transfer of bare ions into the mass analyzer via gas flow from AP to the subAP.

*Vacuum* ionization methods, such as electron ionization and chemical ionization, do not employ a matrix. These methods are limited to compounds that, upon heating, have a vapor pressure partly due to intact molecules. MALDI^5,8^ and matrix-assisted ionization (MAI) from vacuum (vMAI)^13^ use a matrix in which the analyte is embedded. Charged particles are likely the means by which most low-volatility analytes reach the gas phase as ions.^14^ In MALDI, a laser beam striking the matrix/analyte sample generates gaseous particles and facilitates desolvation. vMAI is a newer ionization process that uses a matrix that sublimes when placed at subAP, producing both low- and high-mass ions without the need for a laser or any applied heat or other energy source. One postulate for the origin of charged particles is a chaotic sublimation process, somewhat akin to chaotic boiling.^15^ These charged particles must shed the neutral matrix to release the bare analyte ions for analysis. Desolvation processes are more effective in producing bare analyte ions at higher gas pressure, thus, less effort has gone into desolvation in the vacuum region before the mass analyzer. Without energy input, the loss of the neutral matrix occurs via evaporation or sublimation and is less complete over a longer period.

Other newer ionization processes, which do not rely on ESI^7^ or APCI^16^ or variant methods, include solvent-assisted ionization (SAI)^17,18^ and MAI^19^ where the ionization process occurs in the region between AP and the mass spectrometer vacuum with new and adaptable sample strategies.^20^ Ionization typically occurs within a capillary tube, which acts as a restriction limiting gas flow into the mass spectrometer’s vacuum. These newer processes eliminate ion losses that occur with AP methods in which a large fraction of ions and/or charged particles are not transmitted into the restriction. Therefore, these newer ionization methods typically transmit a larger fraction of the generated charged particles into the vacuum region.^15,21^ Even though commercial mass spectrometers often have decades of ESI optimization, the newer ionization methods can exceed those of ESI for certain important classes of compounds.^22,23^ By employing desolvation in the vacuum region, the sensitivity of ionization methods, under conditions of incomplete desolvation in the source region, can be enhanced. In either AP or *inlet* ionization, valuable analyte is trapped in particles, which may contribute to background ions, but not analyte ions. One means available to partially circumvent this problem is to use liquid or solid matrices, which more rapidly evaporate/sublime in vacuum. Evaporative cooling of gaseous particles can reduce the effectiveness of this approach.

The proof-of-feasibility technology presented here enables rapid and accurate chemical and biological analyses using various sources and sampling methods, including filter paper, swabs, and meshes, with options for enrichment or cell culture with challenging samples. The multiport vacuum chamber assembly enables rapid switching of ionization methods without venting. The system is user-friendly, allowing most users to operate a single port while using additional ports only when needed. The device can be retrofitted to existing mass spectrometers and adapted to other ionization and sampling devices as necessary, thus offering unparalleled ease and comprehensiveness at a relatively low cost, which is crucial for more effective measurements during physician visits, airport screenings, and health crises, such as pandemics and cancer progression monitoring.

## Experimental and Methods

The concept and technology were developed on a Q-Exactive Focus mass spectrometer (Thermo) and adapted for use on a SYNAPT G2S (Waters). Materials and other pertinent details, including but not limited to background information, details on the multiport operating features and the Surface Pen based on the concept of the Surface Reader Device schemes, photos, movies, and mass spectra can be found in the Supporting Information.

### General Aspects of the Multi-Port Vacuum Assembly Device

Compact vacuum chamber assemblies that allow immediate access to various ionization methods for API mass spectrometers are shown in Schemes S1 and S2. The assembly offers an array of possibilities, only a limited subset of which is selected for display and discussion below. The design enhances both ion and charge transfer, while maintaining sufficient sealing of the analyzer from AP, eliminating the need to vent between uses and thereby streamlining operations, reducing downtime, and reducing the need for specialized expertise. The vacuum assembly can employ variable gas flow and/or ion optics to transfer ions and other charged particles into the analyzer. Ports can also interface with auxiliary devices to enhance ionization processes, such as, e.g., using heat and/or collisions to improve desolvation (e.g., ESI) and ionization (e.g., SAI). Sampling devices operating on the basis of SAI enable flexible operations and distance (remote) sampling, including technologies that analyze surfaces remotely. Unlike MALDI or MALDESI,^24,25^ SAI ionization occurs primarily in the inlet tube, which, depending on the matrix used, may be heated, e.g., Sauereisen or other heater elements powered by a Variac.^1,20^ The sampling approach and success are, to a significant extent, related to the mass spectrometer vacuum system, which draws the sample into the subAP region, where ionization occurs on the fly. Because of the high sensitivity and small sample sizes, instrument contamination during normal use is about the same as with ESI. Shutters or valves enable cleaning the assembly without venting the instrument, while small-diameter tubes facilitate the secure exchange of AP ionization (API) sources. The multiport vacuum assembly supports multiple aspects of obtaining an answer promptly and efficiently.

### More Detailed Aspects of the Multi-Port Vacuum Assembly Device

The multiport assembly is machined from a single metal block with a conduit passing lengthwise through the block. Typically, this conduit is on the order of 6 mm in diameter with threaded ends to allow fittings to attach an inlet tube at one end and interface with the mass analyzer at the other end. Ports are added by drilling conduits at right angles and intersecting the lengthwise conduit. A port may be a tube with an inner diameter (ID) typically between 0.3 mm and 7 mm, depending on the mass spectrometer’s pumping capacity and whether the port is for AP or *inlet* ionization or for *vacuum* ionization. If for AP, gas flow similar to that used with the commercial API inlet is used. A port can be open to AP or sealed when not in use. Some ports enable gas flow for processes such as sublimation while maintaining the analyzer’s pressure stability. Ports may vary in inner diameter, and gas flow through them is crucial for achieving the desired pressure in a vacuum chamber, typically between 10 mbar and 10^–2^ mbar, which is optimal for the downstream analyzer’s operation. There is no need for additional pumping, but with low pumping capacity instruments, it can be added to improve sensitivity with larger ID tubing.

The multiport vacuum chamber assembly is readily retrofitted by replacing the default commercial ionization source of the respective mass spectrometer. Different ports can be used in synergy, allowing fluid communication between the multiport vacuum chamber and the analyzer device, and, if desired, the commercial ion source. Adding ports can enable *vacuum* ionization methods and improve laser beam interaction with samples, such as in MALDI. Additionally, auxiliary ports can introduce low gas flows to enhance ionization rates and support the transfer of charged particles into the analyzer. Various functionalities may include a laser for laser-based methods such as MALDI, a shutter for device exchange without venting, and the introduction of reaction gases for various processes.

### AP, Inlet, and Vacuum Ionization Sources and Methods

The multiport vacuum chamber assembly connects the vacuum chamber conduit to the subatmospheric pressure (sub-AP) of the analyzer device, using available ports to facilitate varied functions while maintaining optimal vacuum conditions. Ports may be equipped with valves or features like capillary inlets and skimmer cones to support gas flow without compromising pressure. Configurations may include API inlets (e.g., ESI and ASAP), *inlet* ionization inlets (e.g., SAI and MAI), and *vacuum* ionization inlets (e.g., vMALDI and vMAI). This assembly enables rapid changes to ionization sources without venting, thereby improving the performance of various methods. It ensures efficient transfer of gaseous ions *and* charged particles to the analyzer, matching the sensitivity and operational capabilities of traditional commercial ionization sources. The design is adaptable to high- and low-performance instruments, providing additional “desolvation” to enhance sensitivity via gas-flow adjustments, laser application, or collisional surfaces within the chamber region (the higher-pressure end of a mass or ion mobility system). Unused ports can be sealed with a solid tube which fits snuggly within the conduit to prevent eddy flow. Upon installing the vacuum chamber assembly on the analyzer, air-flow is optimized for the configuration.

#### Sample Preparation and Materials

Materials can be cut or used as is; refer to Supporting Information for additional information. Sample preparation is not critical.^1,20^ The interested reader is referred to the work of LSI,^26^ MAI,^13,19^ SAI^17,18^ and related source and sampling configurations. Specific examples include bacteria,^20^ drugs,^18^ uranium^20^ sample preparation and analysis previously described.

### Sampling Devices

The multiport vacuum chamber assembly can enable remote sampling via long tubes made of materials such as PEEK, fused silica, PTFE, silicon, and metal. The design supports multiple ionization methods, enabling simultaneous or sequential analysis across different devices while managing temperature, gas flow, voltages, and ion transfer. This assembly can be connected to existing MS or related analyzer systems by replacing the commercial ionization source with a vacuum-tight flange. Although the initial installation of the vacuum assembly requires venting the instrument, it creates a seal for effective operation. However, using a commercial API source may limit the number of available ports and restrict voltage access for specific functions. One of the ports might incorporate a capillary tube connecting the higher-pressure exterior with the lower-pressure vacuum region. These tubes, which range from less than 1 mm to over 1 m in length (for remote sampling) and with inner diameters of typically 0.1 to 3 mm, efficiently transport gas-phase ions *and* charged particles. The gas, often air, but potentially other gases such as nitrogen or argon, is routed to the analyzer for further processing of the arriving ions and charged clusters.

## Results and Discussion

### General Implementation of Analytical Innovation

A vacuum chamber assembly is designed to accommodate multiple ports (Schemes 1, S1–S4). This assembly enables diverse combinations of traditional and newer ionization technologies to address various challenges, all with a single mass spectrometer, providing speed and ease of use. The device supports a range of applications across disciplines such as biology, chemistry, health, forensics, and environmental science, ensuring versatility, rapid throughput, and cost-effectiveness among other attributes. The innovation facilitates easy sample presentation for MS analysis from AP for use with AP, *inlet*, or *vacuum* ionization methods. Quantification is achievable using internal standards. Ions *and* charged particles are transmitted from different ionization sources into the analyzer eliminating the need to vent the instrument transitioning between different ionization techniques. Additionally, auxiliary ports can augment existing functions or introduce new capabilities for analytical measurements. Specific examples are provided below.

**Scheme 1:**
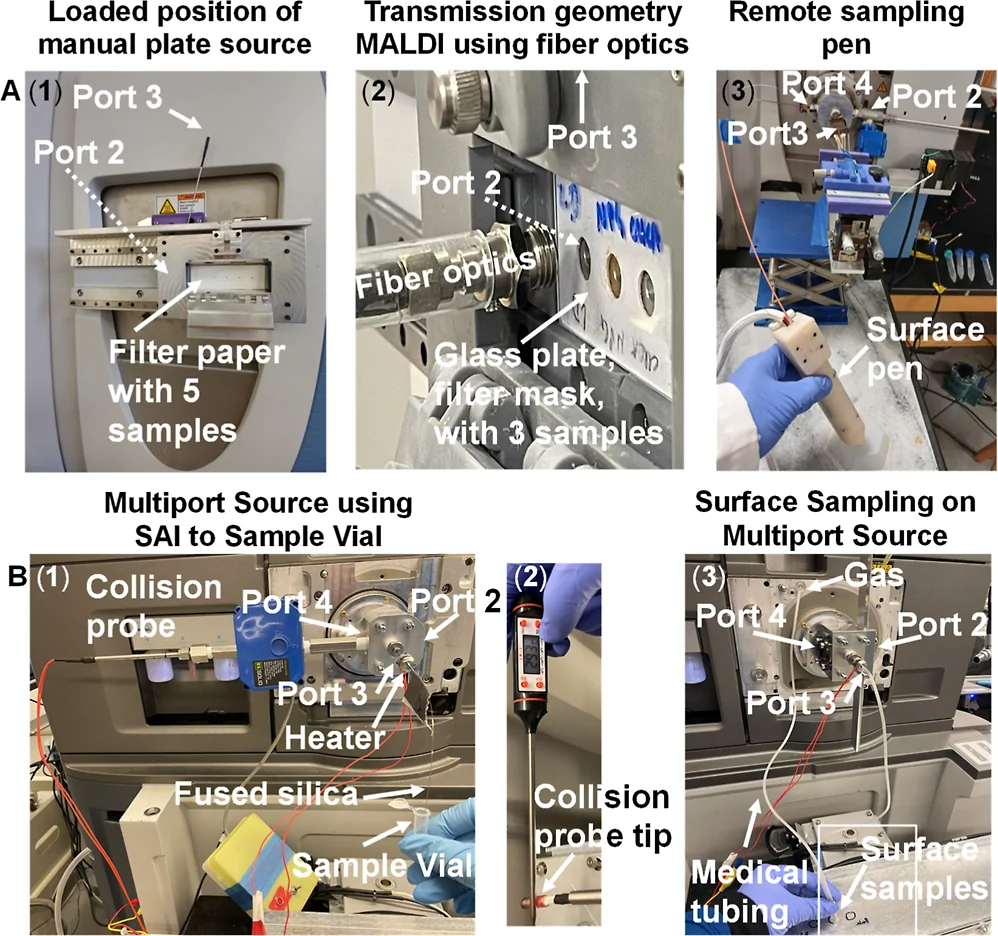
Photos of Multiport Sources on Two Different Mass Spectrometers: (A) Thermo Q-Exactive Focus, (**1**) 3-Port Plate Source (vMAI) and SAI, ESI, or Auxiliary Port, (**2**) 3-Port Plate Source (MALDI, LDI) and SAI, ESI, or Auxiliary Port, (**3**) 4-Port Remote Surface Sampling Source (SAI), vMAI Probe Source, and SAI, ESI, or Axillary Port; (B) Waters SYNAPT G2S (**1**) 4-Port Plate Source (vMAI) and SAI, ESI, or Auxiliary Port, and/or Collision Surface Probe; (**2**) Displays the Collision Probe Heated by the Variac; 3-Port Plate Source (MALDI, LDI); (**3**) 4-Port Plate Source (vMAI) and SAI, ESI, or Auxiliary Port, and Laser Window to Perform Laser on Port 2 in Reflection Geometry in Addition to the Transmission Geometry Alignment. There are Many More Options and Combinations, Only a Few Additional Variations are Added to the Supporting Information. The Pressure Within the Vacuum Chamber is that Entertained by the Vacuum Pumps of the Respective Mass Spectrometer. Port 1 is in Fluid Communication With the Mass Analyzer. Additional Photos are provided in the Supporting Information

### Specific Examples of Analytical Technology

The present work exemplifies the capabilities of analytical innovation through the rapid introduction of samples into vacuum by redefining ionization sources and illustrating the potential applications in MS (Schemes 1, S1–S4). All multiport sources have a port that interfaces with the mass spectrometer vacuum, at least one API port for ESI, APCI, SAI, MAI, and ambient ionization methods, and at least one port for *vacuum* ionization methods such as vMAI and MALDI. The simplest being a 3-port design (Scheme S1). A 4-port configuration (Scheme S2) provides an additional port that may, for example, be used to transmit a laser beam to the vacuum port used for vMALDI operation. Various ionization techniques, such as intermediate pressure MALDI, vMAI, ESI, and SAI, can be used, as well as sample supports and sampling devices, with examples illustrated in Schemes S3 and S4. Videos (Movies S1–S12) are included to demonstrate the process of data acquisition and the consecutive use of different methodologies. We highlight the versatility of this approach by analyzing an array of sample applications showcasing the effectiveness of multiple ionization methods on a single mass spectrometer. This is made feasible by the vacuum-tight assembly, incorporating multiple ports that can be retrofitted by customers, as discussed in the subsequent sections and the Supporting Information. This technology is not confined to a particular vendor, further enhancing its applicability.

Examples of the utility of having a 3-port source with multiple ionization methods readily available on a single mass spectrometer are presented below. A defined mixture comprising a drug, carbohydrate, glycopeptide, peptide, and small protein was analyzed using the methods of vMAI (Figure 1A1) and SAI (Figure 1A2). A so-called plate source, which allows matrix/analyte samples to be rapidly exposed to vacuum where ionization spontaneously commences, serves as the origin for vMAI (front port, Scheme S1), while SAI is conducted by passing solution through the inlet tube (top port). Both ionization methods yield excellent ion abundances with minimal chemical background; however, it is noteworthy that only SAI demonstrates the capacity to ionize maltopentose as a protonated ion at *m*/*z* 829. vMAI operates without additional energy input, in this case with the matrix 3-nitrobenzonitrile (3-NBN), resulting in its preferential ionization of relatively polar and basic compounds and little propensity for ionization with, e.g., Na^+^. In contrast, SAI employing water or methanol/water as well as heating the inlet tube is capable of ionizing a broader range of substances, including polymers.^27^ In this case, switching ionization methods is accomplished by the selection of the port for sample introduction. This example highlights the importance of employing multiple ionization methods, but also a note of caution. The more comprehensive the ionization method, the more complex the mass spectra obtained with direct ionization methods, including more background ions. For many analyses, better results are obtained by selecting an ionization method or conditions that are more specific to the desired analysis.

**Figure 1:**
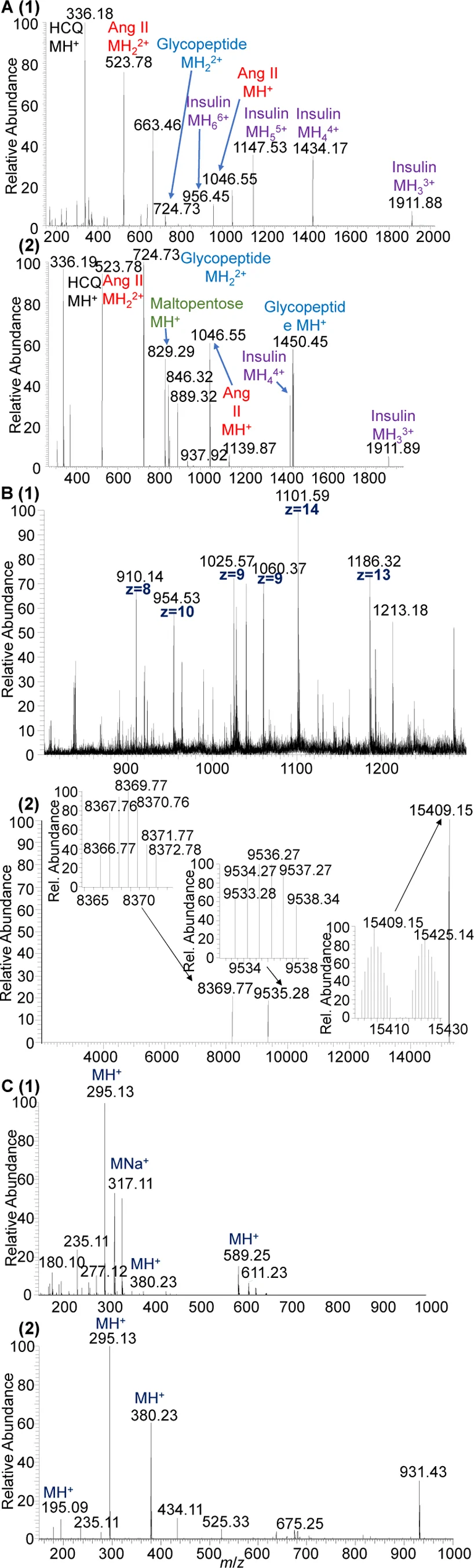
Mass spectra based on multiport vacuum assembly system equipped with AP, *inlet*, and *vacuum* ionization: (A) model mixture (drug, carbohydrate, glycopeptides, peptides, and small proteins) (**1**) vMAI (plate based) and (**2**) SAI (inlet tube heated to ∼120 °C; (B) vMAI (plate-based) of bacteria *B. Cereus* exploiting multiply charged ions in the region (**1**) between *m*/*z* 700 and 1300 and (**2**) deconvoluted to the molecular mass showing isotopic contributions in insets; and (**C**) direct analysis of 1-propionyl lysergic acid diethylamide (1P-LSD, *m*/*z* 380) spiked into Diet Coke (*m*/*z* 295 (aspartame) and *m*/*z* 195 (caffeine)) by (**1**) direct ESI (inlet tube heated to ∼ 160 °C) and (**2**) vMAI using the probe-based introduction. Vacuum chamber assemblies retrofitted on a Thermo Q-Exactive Focus. (**A**) and (**B**) on 3-port system, and (**C**) 4-port system.

For ionization by SAI, only the inlet tube of the multiport source is necessary, along with a means to introduce the sample in a solvent stream or solvent droplets (particles). In this case, the inlet tube is the ion source and no additional hardware or electronics are necessary.^17,18^ API and vacuum results are generated within seconds of one another. The SAI port can also be used with ESI, APCI, and various ambient ionization methods, provided the requisite sources are available to interface with the inlet tube, which is typically heated similar to when used for API methods. For vMAI, only a means to introduce the matrix/analyte sample into a vacuum is necessary; either a solid probe or the plate source can serve this function.

Both vMAI and SAI produce multiply charged ions, similar to ESI, thereby extending the mass range of API mass spectrometers. Although SAI and ESI ionize proteins, these methods are less suitable for direct characterization of bacteria using bacterial proteins as biomarkers without using time-consuming sample cleanup methods, partly because these methods provide more universal ionization and thus complex mass spectra with high background. vMAI offers a sensitive and straightforward means of bacteria classification and identification. As an example, bacteria were grown on agar plates overnight, and for each, a small portion of the resulting colonies was scraped off and individually lysed with 70% formic acid. Diluting this lysate solution with ethanol provided a solution from which 1 μL can be added to filter paper and dried. Adding 1 μL of a 4:1 mixture of 3-NBN and α-cyano-4-hydroxycinnamic acid (CHCA) in a 4:1 solution of acetonitrile (ACN): water (the matrix solution) to the dried sample on the filter paper provides a substrate ready for introduction into the multiport ionization platform for vMAI MS. The plate-source component of the multiport source was used to insert this sample into the vacuum region of the mass spectrometer, where charged matrix/analyte particles are spontaneously emitted and transferred toward the ion optics of the mass analyzer by a gas flow from higher to lower pressure. In this case, an installed inlet tube upstream from the vacuum source can be configured to supply a low flow of gas, or to cut off gas flow altogether, using special caps that fit over the inlet tube entrance when not in use. As noted previously, mass analysis requires complete matrix removal to release gaseous bare ions, a process facilitated by gas flow.

Figure 1B­(**1**) displays the *m*/*z* region between 700 and 1300 for the bacteria *B. Cereus*, and Figure 1B­(2) displays the deconvoluted molecular ions from the original mass spectrum. Within the data acquisition window are singly charged signals representing ions from, *e.g.*, metabolites and lipids, and multiply charged signals from proteins. The simplicity, speed, and low cost of the vMAI method using the multiport source on common API mass spectrometers are clear advantages for this analytical technology. Interestingly, the proteins detected by vMAI show more overlap with those detected by MALDI than by ESI. All of these methods sample only a small fraction of the proteome, yet produce protein profiles that are sufficiently different and reproducible to classify and identify bacteria, especially when combined with methods such as artificial intelligence (AI) and machine learning (ML).

### Adaptation to Different Mass Spectrometers

The multiport device can potentially be adapted for use with most API mass spectrometers. For the work presented here we intentionally used a mass spectrometer that had been challenging in early studies of *inlet* ionization.^28–31^ In addition to effective ionization at pressures ranging from AP to intermediate vacuum, ion and charged particle transmission from the source region into the ion optics of the analyzer needed to be considered. Although applying extraction and voltages to guide charged particles could be effectively used, it greatly increased the complexity. Gas flow was found to be a better and easier option for transmitting ions, and charged particles, with all of the ionization methods. As noted above, the inlet tube(s) used with API and *inlet* ionization provide their own gas flow when being used, and if located upstream of the port used for *vacuum* ionization, can provide a controllable gas flow to aid ion formation and transfer, including charged particles. However, for vMAI, the use of filter paper for a sample/matrix substrate provided a simple and more effective gas flow, especially considering that filter papers can be selected to provide a reproducible and ideal gas flow.

Implementation of appropriate *inlet* and *vacuum* ionization conditions using the multiport source demonstrated effective performance using either the new or traditional ionization methods. Notably, the mass spectrometer employed in this study allows for instantaneous gas-phase separation via ion mobility. With the multiport source, the ion mobility feature can be used with any of the available ionization methods, including MALDI with, *e.g.*, the 4-port design (Scheme S2). Thus, a one-time retrofit with one of the multiport devices on instruments with rather expensive features, such as ion mobility separation, greatly enhances their utility. For example, the enhanced gas-phase separation capability of ion mobility of multiply *versus* singly charged ions (Figure S1) is used to increase the visibility of multiply charged ion signals amidst the predominant signals from singly charged lipids and other compounds.

Movie S1 showcases the application of the plate source used in vMAI and SAI modes on the 3-port inlet interface. A plate holding an inlet tube slides over a flat flange having a channel to the instrument vacuum region, creating a vacuum seal except when the inlet tube exit is directly over the inlet of the channel. The plate source inlettube provides a ready means of remote sampling using surface SAI (Figure S2), in which a small drop of solvent is dropped onto the surface of fungi, facilitating the extraction of chemical components that were subsequently delivered to the mass spectrometer through an extended fused-silica line, approximately 0.5 m in length, utilizing the vacuum of the instrument for transport and ionization. The extracted material is ionized in situ within the inlet tube plate source of a 3-port interface. The inlet tube did not need to be heated as sufficiently volatile solvents such as methanol or isopropyl alcohol were used for extraction, and these solvents, when exposed to subAP conditions, form charged droplets which are capable of desolvation in subAP without added heat.

Increasing the number of ports beyond three offers additional capabilities while maintaining the ease of use and sensitivity of the 3-port interface (Scheme S1). Notably, ports can differ in quantity, orientations, and dimensions, and they may function to enhance another port and its specific ionization source and methodology. Illustrative examples are provided for 4-port systems utilizing mass spectrometers from different manufacturers (Scheme S2).

A comparison of ESI (Figure 1C1) and vMAI (Figure 1C2), one operating from AP and the other from vacuum conditions, for the direct analysis of 1-propionyl lysergic acid diethylamide (1P-LSD, *m*/*z* 380) spiked into Diet Coke, clearly demonstrates the differences in the two ionization methods, especially relative to the *m*/*z* 380 signal in direct ionization. When employing vMAI 1P-LSD stands out as a much more significant signal. However, both ionization methods detect 1P-LSD in pure solution, but vMAI is more effective at detecting it in Diet Coke than ESI because vMAI is a more selective ionization method. Selectivity in ESI and other ionization methods can be achieved by selection of additives. On the other hand, both ionization methods detected aspartame (*m*/*z* 295), a sweetener present in some diet beverages, but ESI detected it as protonated and Na^+^ adducted. The simplicity and speed of ionization using vMAI, especially with filter paper as the substrate, along with its different ionization selectivity, make this ionization method an excellent option for direct analyses requiring less than 10 s, including inserting and removing the sample from vacuum. When trained in using direct analysis with ESI-based methods and with vMAI, students gravitate to the use of vMAI because of its ease of use.

The extra port of the 4-port system can hold a second tube, which can be used as another inlet, or to control gas flow into the vacuum region in order to achieve improved ionization, ion/charged particle transmission, and/or charged particle desolvation when using vMAI, while not interfering with the inlet tube used for API and *inlet* ionization. Both inlets need controlled gas flow so as not to unduly increase the pressure in the ion source region. Typically, only one inlet is open at a time and the other is capped. As an example, analysis of a protein mixture containing carbonic anhydrase and human transferrin via the heated inlet tube port revealed characteristic ESI ions with multiple-charge-states (Figure S3). With the ESI inlet capped and a controlled gas flow through the second inlet tube aimed directly at a surface for which carbonic anhydrase (CA) (∼29 kDa) was applied with the 3-NBN matrix, multiply charged ions from CA were detected using vMAI from probe sample introduction (Figure S4A). It is postulated that the gas flow directs the charged particles toward the mass analyzer and assists in desolvating the matrix in vMAI under low-pressure conditions, as well as enhancing the rate of sublimation, creating the initial charge separation conditions. Additionally, Figure S4B shows a mixture of drugs ionized by vMAI from a 100% DMSO using probe introduction on the 4-port inlet. This is challenging with alternative ionization methods. In the pharmaceutical industry, drugs are frequently solubilized in DMSO, which poses analytical challenges due to its high viscosity and relatively low volatility. Utilizing the same 4-port assembly, SAI was employed for quantitative analyses (Figure S5) incorporating internal standards with the heated inlet tube port, while the other two ports remained dormant.

Depending on the need, the 4-port interface can offer a variety of options, including a port for a laser beam window. The auxiliary port can be aligned to provide a line of sight to the *vacuum*-ionization port. A laser beam with a focusing lens, possibly using fiber optics, can be aligned to focus on the substrate surface on which a matrix/analyte sample is inserted into a vacuum through the vacuum port. This arrangement allows intermediate pressure MALDI analyses. For high throughput, a substrate holding multiple samples can be loaded into position using the plate source, or, for a less costly option, the sample port can receive a probe device to load a single sample at a time. Substrate movement can be achieved with either configuration to present a fresh sample surface to the laser beam. In either configuration, both MALDI and vMAI are always available. Such a configuration is depicted in Movie S2 for the analysis of oxycodone, a substance significantly contributing to the drug epidemic. Although the use of a laser with MALDI matrices successfully detects a variety of compounds as singly charged ions, its implementation often results in increased chemical background, a byproduct that prompted the development of matrix-free methodologies for low-mass compounds.^32^ In another example, the sample is transported from atmospheric pressure into the vacuum of the 4-port inlet via a probe inserted through an isolation valve to initiate ionization. The chemical background remains sufficiently low relative to the drugs fexofenadine and azithromycin (Figure S6). Perfluorooctanesulfonic acid (PFOS) was also ionized by intermediate pressure MALDI on the 4-port inlet as demonstrated in Figure S7. The incorporation of a laser introduces additional complexity and costs that are not justified for analyzing these compounds, but may be useful for compound types where MALDI is commonly employed, while keeping in mind the mass limit of API mass spectrometers for singly charged ions.

### Specific Examples of Applications

#### Sampling of Surfaces from Plants to Foods

Direct surface analysis is increasingly gaining attention.^1,18,20,33,34^ As shown in Figure 2 and Movie S3, living or dead surfaces can be analyzed rather effectively by surface SAI to obtain different chemical fingerprints of, for example, fresh leaves *versus* flower petals and fresh *versus* aged flower petals. Equally straightforward is the analysis of tissue sections (Figure S8). In addition to ionization efficiency, SAI has the advantage that the instrument vacuum provides the pressure differential to transfer the sample from an AP environment into the inlet-tube ion source, readily allowing remote sampling with or without a laser (Figure S9).

**Figure 2:**
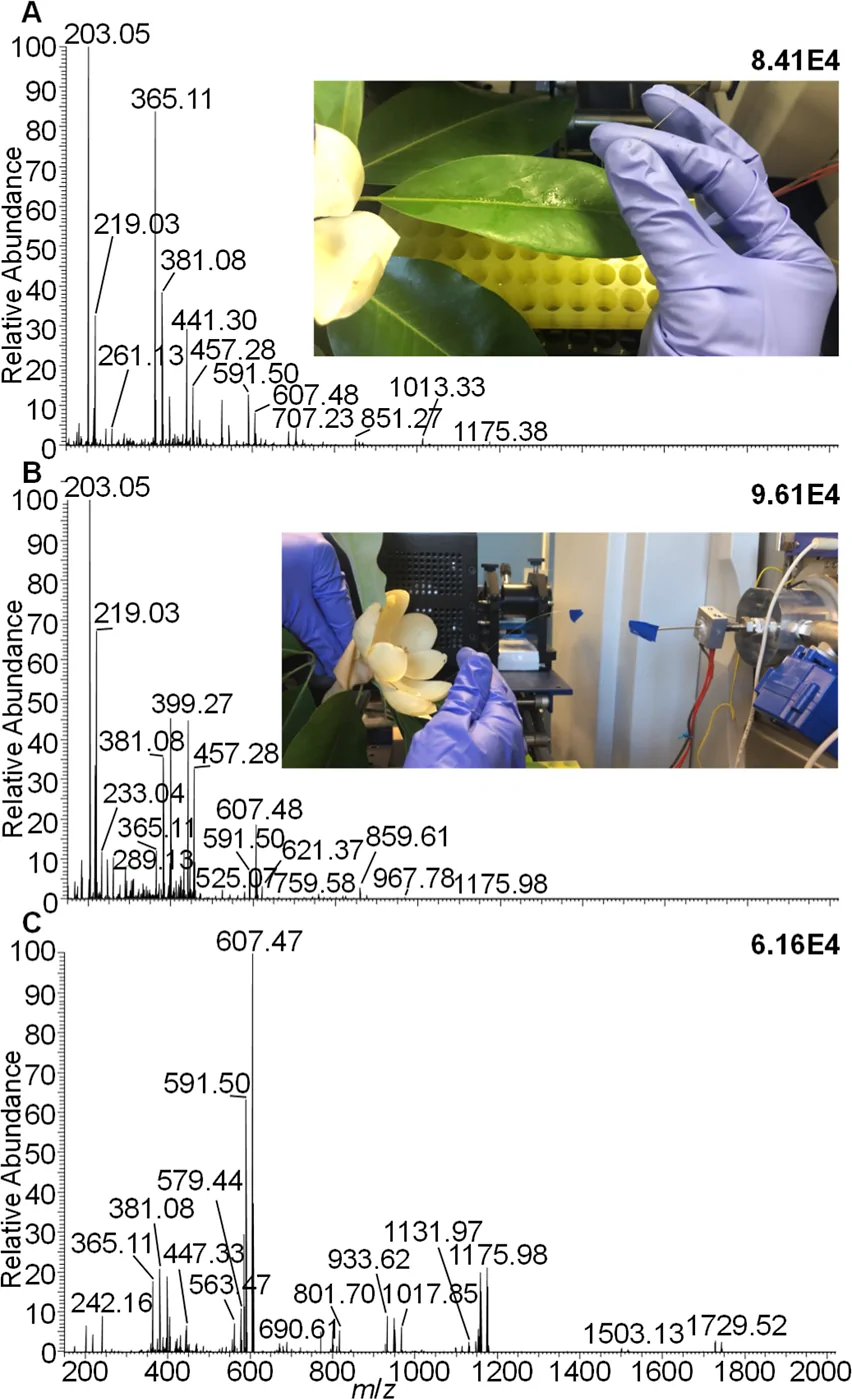
Surface SAI remote sampling of surfaces such as flower components of (A) leaf, (B) fresh flower and (C) dry (old) flower providing unique *m*/*z* fingerprint patterns. Methanol was added (30 s) before being drawn by the pressure differential through an extended fused-silica tube into the MS inlet tube. Photos of the samples provided for illustration purposes. 4-Port vacuum chamber assembly retrofitted on the Thermo Q-Exactive Focus. Inlet tube temperature ∼120 °C.

For this work, the API-only source housing and inlet of a Waters mass spectrometer were replaced with a 4-port inlet featuring a heated inlet tube for API ionization and either a probe or plate source for vMAI. For vMAI on the probe, the sample and matrix are applied to the probe tip; for the plate source, the sample is typically applied to filter paper used as the sample substrate. For remote SAI, an extended inlet tube is used to transfer and ionize the sample. SAI analyses of strawberry surfaces (Movie S4, Figure S10A), mouse brain tissue sections (Movie S5, Figure S10B), and beef (Figure S11) demonstrated that, depending on the solvent used for extraction, different compounds could be detected. Chicken, beef, shrimp, and pork were also analyzed using the vMAI probe source (Figure S12). Petals were analyzed using the vMAI plate source with filter paper within seconds of each other on the same mass spectrometer without signs of instrument contamination or carryover (Figure S13). When spatial resolution is needed, laser ablation can be included (Figure S14), as previously reported using a Thermo mass spectrometer.^1^ By incorporating an ESI emitter, ionization by ESI is readily achieved, but with extra effort and often carry-over due to the nature of the ESI method. Although MALDI is possible with the 4-port design, the extra expense and difficulty would warrant its use only for analyses that require, for example, spatial resolution.

#### Forever Chemicals Fueling Environmental and Health Concerns

PFOS analysis is much more sensitive when using either SAI or ESI than when using MALDI or vMAI. SAI performs more effectively than ESI in 100% aqueous samples, the solvent in which it is frequently accumulated and ingested by wildlife and humans, provided sufficient heat is applied to the inlet tube. As concentrations of more volatile solvents, such as methanol, increase, the sensitivity of ESI measurements increases more than that of SAI measurements, making ESI preferable. Therefore, for direct PFOS analyses, having both ESI and SAI readily available provides enhanced sensitivity across water, organic, and mixed solvent systems. Furthermore, SAI operates efficiently without heat when utilizing a volatile solvent composition such as low-content aqueous methanol. PFOS can also be enriched by bubbling, *e.g.*, nitrogen gas, through water containing PFOS, and collecting the droplets formed by the bursting bubbles directly into an extended inlet tube when employing SAI. The ascending bubbles are enriched in PFOS as documented in Movies S6 and S7. The distance between the inlet tube and the solvent surface significantly affects the sampling process, with more droplets collected when the tube opening is closer to the surface, but not sampling the bulk solution (Figures 3, S15). The concentration enhancement achieved through the PFOS sampling process is illustrated in Figure S16. The 4-port interface with two inlet tubes, always installed, would allow the user to acquire PFOS samples using both ESI and SAI to achieve optimal results without requiring any physical changes.

**Figure 3:**
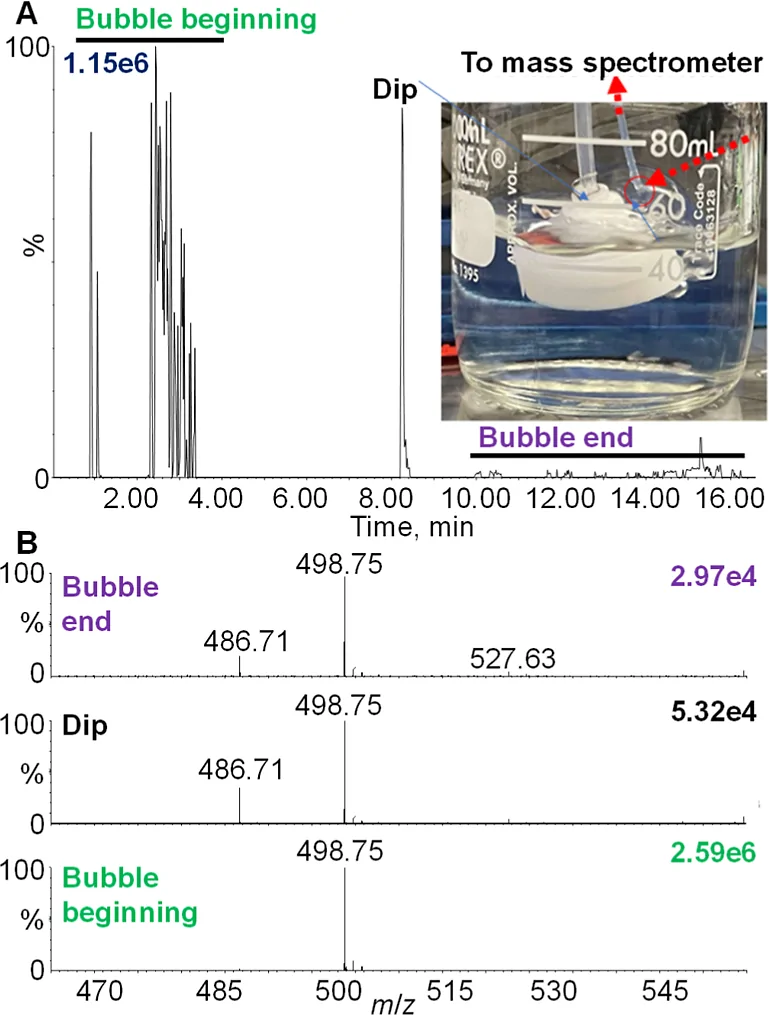
PFOS in 100% water sampled using bubble chemistry and direct ionization by remote sampling for SAI of the ascending enriched droplets from bubble bursting: (A) Chronogram and (B) mass spectra at 3 different times. Ion abundance differences are related to PFOS concentration in the sampled solution. 4-Port vacuum chamber assembly retrofitted on the Waters SYNAPT G2S. Inlet tube temperature ∼150 °C. Refer to Movies S6 and S7.

In addition to diluted PFOS samples that were concentrated and directly sampled using surface SAI, the soil of basil plants was wetted with contaminated PFOS water. Interestingly, PFOS was detected across the entire plant surface (Figure S17) by simply adding a drop of water to the plant material and using remote SAI to draw the sample into the inlet tube. More research will be needed to determine whether PFOS contamination in the soil enters the plant through the plant roots or through the air.

#### Drugs Fueling an Epidemic

While ESI and SAI, along with APCI, are suitable methods for drug analysis, direct vMAI analyses on filter paper offer several advantages. For example, the filter or chromatography paper provides a substrate for a quick, simple cleanup step offering an extremely easy-to-use analysis approach without carryover, even with high-concentration samples. It also provides a means of storing a sample for later analysis, low detection and quantification limits, and almost no instrument contamination because only volatile samples and the extremely small amount of nonvolatile analyte leaving the substrate surface in matrix particles remain in the mass spectrometer after removal of the filter paper. The volatile compounds, including the matrix, are pumped from the instrument. Without a high-energy source, such as a laser or an electric discharge, reactive species, such as radicals, are not formed, further contributing to cleanliness. Because of these advantages, especially the ease of use, having vMAI always available will lead users to select it as the method of choice, for their own projects.

As an example, a comprehensive analysis of urine samples collected from a volunteer suffering from a back injury and prescribed oxycodone was conducted over 44 h using vMAI with filter paper (Figure S18). Notably, the data were obtained with an instrument that had not been recently calibrated. In the absence of MS/MS, the interpretation of these results would have been misleading. At 5 h postingestion, mass-selected fragmentation accurately identified oxycodone directly from the urine without any sample cleanup by utilizing its distinctive mass spectral fingerprints at *m*/*z* 298, 256, and 241. Other drugs, purchased from reputable vendors, such as fentanyl, which is a significantly more lethal street drug, were also readily detected in synthetic urine at concentrations orders of magnitude below its LD_50_. The MS and MS/MS fragmentation of fentanyl, morphine, and cocaine show the expected transitions in Figures S19 and S20. Quantification of hydrocodone in a mixture is shown using hydrocodone-d6, with *R* ^2^ = 0.991 (Figure S21).

Accumulative results indicate that vMAI-MS and MS/MS are particularly effective for analyzing hazardous drugs without requiring prior sample cleanup, especially using disposable filter paper as a sample substrate, thereby enhancing the safety of the operator. The filter paper can be precoated with the matrix, eliminating the need for the user to add it. In this case, urine is added either directly or after a 1:10 dilution onto the precoated filter paper and briefly dried before being placed on the plate source for insertion into the vacuum, where ionization occurs spontaneously.

In a simulated scenario of in-field analyses, fentanyl was deposited on a surface within a fume hood and analyzed in accordance with the methodology illustrated in Figure 4 and Movie S8. The use of accurate mass measurements and MS/MS ensured the reliable identification of the drug directly from the filter paper used to wipe the contaminated surface, which also contained typical contaminants associated with such environments. The operator’s safety was maintained, as only a matrix solution needed to be dropped onto the filter paper before quick insertion into the mass spectrometer’s vacuum for ionization at subAP. The instrument was also safeguarded, as the “clean” unwiped surface of the filter paper was exposed to the subAP of the mass spectrometer. In this case, the ions that are detected originate from material that passes through the filter paper, possibly as charged particles and ions, thus facilitating an initial cleanup.

**Figure 4:**
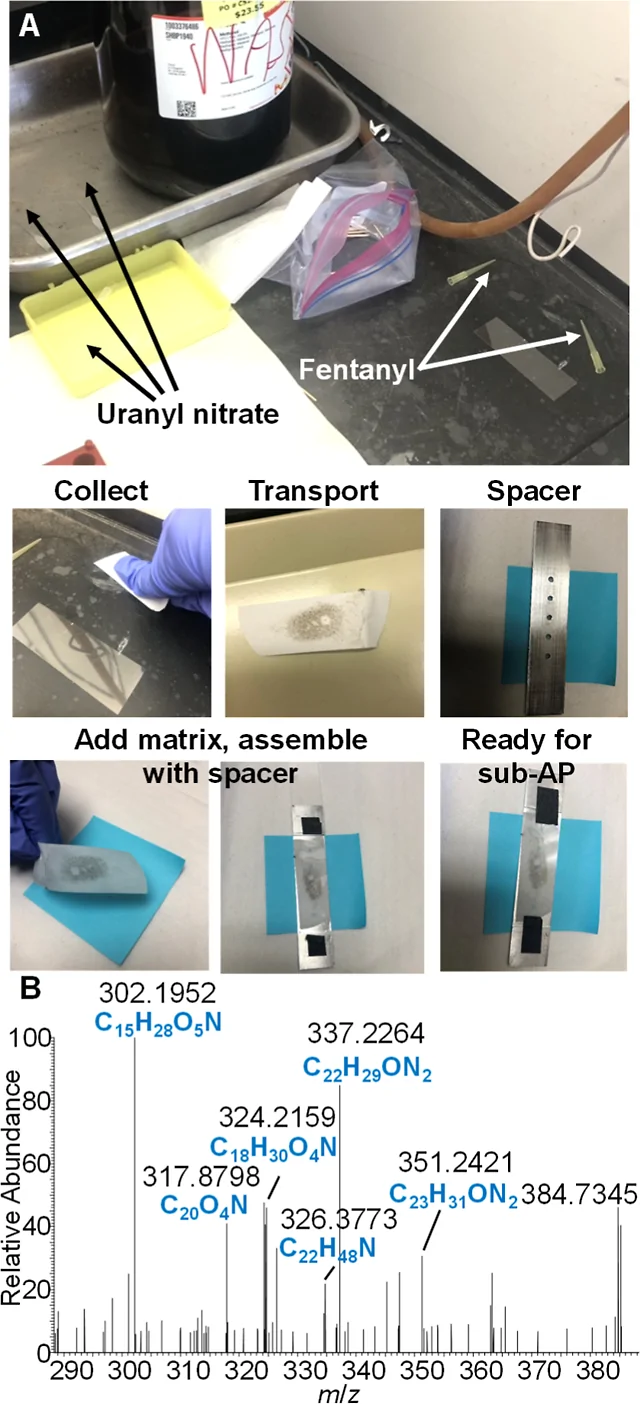
(A) Photographs of 10 nanomole of fentanyl deposited in a fume hood, collected by wiping fentanyl from a countertop with filter paper and adding 3-NBN solution to the visibly “dirty” area of the filter paper, letting it dry and use the “clean” side of the filter paper to face the spacer plate and therewith the sub-AP of the vacuum chamber device and (B) mass spectrum of fentanyl readily identified by the accurate mass; also refer to Movie S8 and Supporting Information Figure 3-Port plate-based vacuum chamber assembly retrofitted on the Thermo Q-Exactive Focus. Uranyl nitrate mass spectra shown in Figure 5

#### Uranium-Containing Samples for Nuclear Monitoring

Concerns regarding uranium compounds in the environment are significant. In this context, we deposited 5 μL droplets of a 10 nM solution of depleted uranyl nitrate in 2% nitric acid on a plastic surface within a fume hood and subsequently sampled the contaminated site after several days. This was achieved by adding methanol to areas where the uranyl nitrate solutions were deposited to partially transfer the sample to a glass microscope slide, which was used as the sample substrate with the plate source after addition of a vMAI matrix solution and dried. Ionization was in negative-ion mode, and MS and MS/MS analyses yielded results in less than 30 s (Figure 5). The sampling method for a contaminated site is flexible and can be tailored to meet the specific requirements of a field sampling initiative. For example, samples can be collected and transported to the mass spectrometer, where ionization and analysis occur without requiring any cleanup protocols. Less than 1 ng of uranyl nitrate was detected by full-acquisition mass spectral analysis.

**Figure 5:**
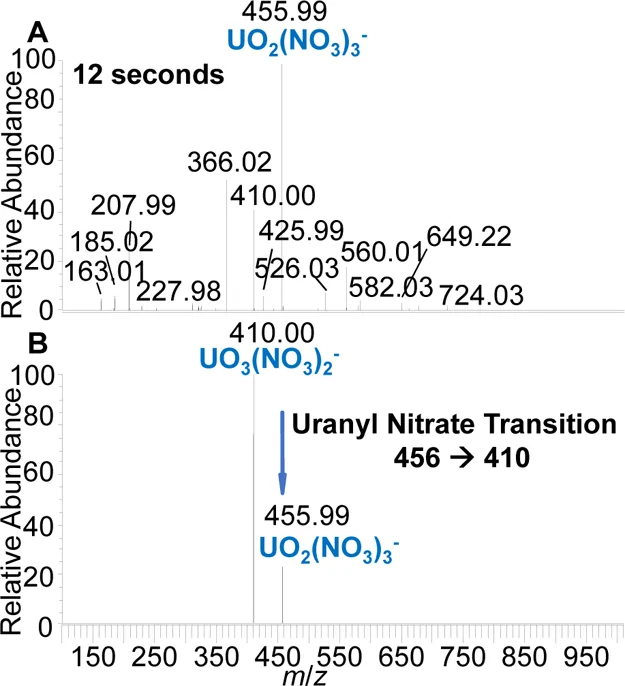
Negative ion vMAI (A) MS and (B) MS/MS of 5 μL of 10 nM of depleted uranyl nitrate solution deposited on a plastic surface in the fume hood, dried, and allowed to sit (age) for 5 days. The dried sample was partially recovered in 20 μL of methanol using a pipet tip and added to 20 μL of a saturated solution of the 3-NBN matrix in 3:1 ACN:H_2_O and 1 μL was applied to a glass microscope slide, allowed to air-dry and acquired using the 3-port plate source assembly retrofitted on the Thermo Q-Exactive Focus.

## Conclusion

The developed vacuum chamber significantly enhances direct analysis and simplifies MS, which may be attributed to several key factors: (1) high ion transmission of the target molecule(s), (2) incorporation of ionization methods that reduce chemical background, (3) heightened sensitivity through selection of best ionization method, (4) robust performance, (5) simplicity of design, (6) operational flexibility, and (7) cost-effectiveness. This innovative approach—utilizing AP, *inlet,* and *vacuum* ionization conditions—holds substantial potential across various application domains, including homeland security, nuclear monitoring, environmental assessment, epidemic and pandemic response, food safety, and cancer research, among others. The seamless integration of these capabilities promises to unveil critical insights into both natural and anthropogenic impacts. Sample support mechanisms, such as paper for specimen collection related to, for example, health, environmental, drug-related, or nuclear concerns, can be efficiently shipped and analyzed in a laboratory environment with high safety and speed. Regarding remote sampling, the implementation of SAI may facilitate this process by enabling real-time in situ analysis on-site. The results obtained from this system could represent a foundational step toward unprecedented advancements in analytical measurement capabilities.

Future developments are anticipated to focus on high-throughput and automated analysis to manage the increasing number of samples, portability for field applications, and real-time data analysis/interpretation/distribution. Furthermore, the usability of field measurement tools must be demonstrated to empower novice users, enabling them to perform analyses on samples of interest in an affordable manner. In an era increasingly characterized by artificial intelligence, the strategic direction is clear, and a path forward to Smart Analytical Infrastructure is evident.

## Supplementary Material



























## Notes

Movie S1: Movie S1_Inlet tube plate_Waters instrument (MP4)
Movie S2: Movie S2_Oxycodone_MALDI in reflection geometry_Waters instrument (MOV)
Movie S3: Movie S3_Flower_Surface SAI Waters instrument (MOV)
Movie S4: Movie S4_Strawberry_Surface SAI Waters instrument (MP4)
Movie S5: Movie S5_Tissue_Surface SAI Waters instrument (MP4)
Movie S6: Movie S6_Bubble chemistry gas cylinder_SAI_Waters instrument (MOV)
Movie S7: Movie S7_Small defined volume bubble chemistry_Thermo instrument (MOV)
Movie S8: Movie S8_Fentanyl recovered from surface_Thermo instrument (MOV)
Movie S9: Movie S9_Surface SAI_larger tube_Thermo instrument (MOV)
Movie S10: Movie S10_Surface SAI_smaller tube_Thermo instrument (MP4)
Movie S11: Movie S11_Surface SAI_double_undergrad_oxy_Waters instrument (MOV)
Movie S12: Movie S12_Surface SAI_double_undergrad_fentanyl_Waters instrument (MOV)
Background on Ionization: brief summary of ionization sources and methods; brief summary on sampling devices and methods; brief summary of the multiple sources on same mass spectrometer; brief summary of the vacuum chamber assembly device; methods; more details to multiport operating features; more details to surface pen based on the concept of the surface reader device; materials section; applications; Supporting Information Scheme S1:3-Port vacuum chamber on thermo mass spectrometer; Scheme S2:4-Port vacuum chamber on thermo and waters mass spectrometer; Scheme S3: various sampling devices (Focus on vMAI); Scheme S4: surface reader devices (Focus on SAI); Figure S1: Fungus, Surface SAI, IMS-MS (Waters); Figure S2: band-aid, surface analyses by vMAI-MS (Thermo); Figure S3: proteins (∼80 kDa), ESI (Thermo); Figure S4: proteins (∼30 kDa), drugs in 100% DMSO, vMAI, (Thermo); Figure S5: quantification of Drugs, SAI (Thermo); Figure S6: drugs, transmission geometry vMALDI (Thermo); Figure S7: PFAS, transmission geometry vMALDI (Thermo); Figure S8:4 mouse brain tissue sections, surface SAI (Thermo); Figure S9:4 blood spots, surface SAI (Thermo); Figure S10: strawberry, tissue, surface SAI (Waters); Figure S11: meat, T junction, surface SAI (Waters); Figure S12:4 different meats, surface analyses, vMAI (Waters); Figure S13: Flower, petals, vMAI (Waters); Figure S14: tissue, laser ablation, liquid junction, surface SAI (Waters); Figure S15: PFOS, water sampling, bubble chemistry (Waters); Figure S16: PFOS, small volume sampling, bubble chemistry (Thermo); Figure S17: PFOS in soil, detected on leaves, surface SAI (Waters); Figure S18: prescription drug, oxycodone in patient urine (Waters); Figure S19: fentanyl, morphine, vMAI, MS/MS (Thermo); Figure S20: cocaine spiked to urine, vMAI, MS/MS (Thermo); Figure S21: hydrocodone, quantification, vMAI (Thermo); Figure S22: drugs, SAI surface reader concept, variation I,II (Thermo); Figure S23:drugs, SAI surface reader concept, variation IV (Waters) (PDF)
