# Supplementary material for: Multi-Port Vacuum Assembly Device Designed for Simplified Point-of-Need Chemical and Biological Analyses Using Mass Spectrometry
Source: Anal Chem. 2026 Aug 22;98(35):25635–45. doi: 10.1021/acs.analchem.6c02029 (PMC13564039; doi:10.1021/acs.analchem.6c02029)
Supplement: Supplementary file 1 [file ac6c02029_si_013.pdf]

Supplementary Information

Multi-Port Vacuum Assembly Device Designed for Simplified Point-of-Need Chemical and Biological Analyses Using Mass Spectrometry

Sarah Trimpin,*[1,2] Ellen D. Inutan,[2,3] Hope Coffinberger,[2,4] Frank S. Yenchick,[1,5] Dushan Kovacevic,[1,5] Sullivan Stimac,[1] John W. Tomsho,[4] Ken Mackie,[6] Milan Pophristic,[2,4] Charles N. McEwen*[2,4]

[1] Department of Chemistry, Wayne State University, Detroit, MI, 48202, USA;
[2] Research & Development, MSTM, Newark, DE, 19711, USA;
[3] Department of Chemistry, Mindanao State University - Iligan Institute of Technology, Iligan City, 9200, Philippines;
[4] Department of Chemistry & Biochemistry, Saint Joseph's University, Philadelphia, PA, 19104, USA;
[5] Singidunum, LLC, Farmington Hills, MI, 48331, USA;
[6] Department of Psychological and Brain Sciences, Indiana University, Bloomington, 47405, Indiana

* Corresponding authors: sarah.trimpin@wayne.edu; cmcewen@sju.edu

Table of Contents

Movie S1: Movie S1_Inlet tube plate_Waters instrument

Movie S2: Movie S2_Oxycodone_MALDI in reflection geometry_Waters instrument

Movie S3: Movie S3_Flower_Surface SAI Waters instrument

Movie S4: Movie S4_Strawberry_Surface SAI Waters instrument

Movie S5: Movie S5_Tissue_Surface SAI Waters instrument

Movie S6: Movie S6_Bubble chemistry gas cylinder_SAI_Waters instrument

Movie S7: Movie S7_Small defined volume bubble chemistry_Thermo instrument

Movie S8: Movie S8_Fentanyl recovered from surface_Thermo instrument

Movie S9: Movie S9_Surface SAI_larger tube_Thermo instrument

Movie S10: Movie S10_Surface SAI_smaller tube_Thermo instrument

Movie S11: Movie S11_Surface SAI_double_undergrad_oxy_Waters instrument

Movie S12: Movie S12_Surface SAI_double_undergrad_fentanyl_Waters instrument

1. **Background on Ionization** *Page S4 ff.*
   - *Brief Summary of Ionization Sources and Methods* *Page S4-S5*
   - *Brief Summary on Sampling Devices and Methods* *Page S6*
   - *Brief Summary of the Multiple Sources on Same Mass Spectrometer* *Page S7*
   - *Brief Summary of the Vacuum Chamber Assembly Device* *Page S7*
2. **Methods** *Page S8 ff.*
   - *More Details to Multiport Operating Features* *Page S8*
   - *More Details to Surface Pen Based on the Concept of the Surface Reader Device* *Page S10-S11*
3. **Materials Section** *Page S11*
4. **Applications** *Page S12*
5. **Supporting Data** *Page S13 ff.*
   - *Scheme S1: 3-Port Vacuum Chamber on Thermo Mass Spectrometer* *Page S13*
   - *Scheme S2: 4-Port Vacuum Chamber on Thermo and Waters Mass Spectrometer* *Page S14*

Scheme S3:	*Various Sampling Devices (Focus on vMAI)*	*Page S15*
Scheme S4:	*Surface Reader Devices (Focus on SAI)*	*Page S16-S17*
Figure S1:	*Fungus, Surface SAI, IMS-MS (Waters)*	*Page S18*
Figure S2:	*Band-Aid, surface analyses by vMAI-MS (Thermo)*	*Page S19*
Figure S3:	*Proteins (~80 kDa), ESI (Thermo)*	*Page S20*
Figure S4:	*Proteins (~30 kDa), drugs in 100% DMSO, vMAI, (Thermo)*	*Page S21*
Figure S5:	*Quantification of Drugs, SAI (Thermo)*	*Page S22*
Figure S6:	*Drugs, transmission geometry vMALDI (Thermo)*	*Page S23*
Figure S7:	*PFAS, transmission geometry vMALDI (Thermo)*	*Page S24*
Figure S8:	*4 Mouse brain tissue sections, Surface SAI (Thermo)*	*Page S25*
Figure S9:	*4 Blood spots, Surface SAI (Thermo)*	*Page S26*
Figure S10:	*Strawberry, tissue, Surface SAI (Waters)*	*Page S27*
Figure S11:	*Meat, T junction, Surface SAI (Waters)*	*Page S28*
Figure S12:	*4 Different meats, surface analyses, vMAI (Waters)*	*Page S29*
Figure S13:	*Flower, petals, vMAI (Waters)*	*Page S30*
Figure S14:	*Tissue, laser ablation, liquid junction, Surface SAI (Waters)*	*Page S31*
Figure S15:	*PFOS, water sampling, bubble chemistry (Waters)*	*Page S32*
Figure S16:	*PFOS, small volume sampling, bubble chemistry (Thermo)*	*Page S33*
Figure S17:	*PFOS in soil, detected on leaves, surface SAI (Waters)*	*Page S34*
Figure S18:	*Prescription drug, oxycodone in patient urine (Waters)*	*Page S35*
Figure S19:	*Fentanyl, morphine, vMAI, MS/MS (Thermo)*	*Page S36*
Figure S20:	*Cocaine spiked to urine, vMAI, MS/MS (Thermo)*	*Page S37*
Figure S21:	*Hydrocodone, quantification, vMAI (Thermo)*	*Page S38*
Figure S22:	*Drugs, SAI Surface Reader Concept, Variation I,II (Thermo)*	*Page S39*
Figure S23:	*Drugs, SAI Surface Reader Concept, Variation IV (Waters)*	*Page S40*

6. Supporting References *Page S41 ff.*

1. Background on Ionization

The background information comprises ionization sources, sampling devices, and methods,[1,2] which are important for understanding the significance of the multiport vacuum chamber assembly for use in mass spectrometry (MS).

Brief Summary of Ionization Sources and Methods

Ionization methods can be categorized by whether ionization occurs at or near atmospheric pressure (AP) or in transition into or directly at sub-AP (vacuum). For nonvolatile and large molecules, a matrix is required for 'soft' ionization with minimal fragmentation: a liquid in electrospray ionization (ESI) and typically a solid in matrix-assisted laser desorption/ionization (MALDI). These ionization methods generate charged droplets or solid particles as intermediate forms that must shed neutral matrix molecules to release the gaseous analyte ions, which are then separated by the mass analyzer based on their mass-to-charge (*m*/*z*) ratio. Available ionization methods include atmospheric pressure ionization (API) techniques such as ESI (*e.g.*, nanoESI, desorption-ESI (DESI), and matrix-assisted laser desorption-ESI (MALDESI)), AP chemical ionization (APCI), AP photoionization (APPI), as well as laser desorption/ionization (LDI), direct analysis in real time (DART), atmospheric solids analysis probe (ASAP), and paper spray. Inlet ionization methods include laserspray ionization (LSI), matrix assisted ionization (MAI), and solvent assisted ionization (SAI), among others. *Vacuum* ionization methods include chemical ionization (CI) and electron ionization (EI), used exclusively for lower-molecular-weight vaporizable compounds, MALDI, and vacuum (v) MAI.

MALDI is a widely used ionization method for MS, particularly on high-vacuum time-of-flight (ToF) instruments. It involves co-crystallizing a matrix with the analyte, which is introduced into the vacuum and ablated using a laser. The matrix, in significant excess, helps generate gas-phase ions from the analytes. Sample preparation techniques such as "dried droplet" and "thin layer" are commonly used to achieve effective ionization. While MALDI can yield high spatial resolution, it faces limitations, including a chemical background, a predominance of singly-charged ions, and challenges in analyzing high-molecular-weight compounds due to broad signal widths. Common MALDI matrices include compounds like alpha-cyano-4-hydroxycinnamic acid (CHCA)[3] and sinapinic acid, which are optimized for stability and absorption at specific laser wavelengths. Matrix compositions can vary, and automated systems are available to assist with sample preparation.

LSI is a laser-based method that generates ESI-like multiply-charged ions from surfaces, utilizing matrices like 2,5-dihydroxybenzoic acid (2,5-DHB)[4] and 2,5-dihydroxyacetophenone (2,5-

DHAP),[5] often producing less chemical background than MALDI. It typically requires a heated inlet tube (≥ 450 °C for some matrices). Vacuum LSI (vLSI) can use matrices such as 2-nitrophloroglucinol (2-NPG)[6] at lower temperatures, operating from AP and high-vacuum conditions. Vacuum matrix-assisted ionization (vMAI), does not require a laser and can desolvate the matrix-analyte sample without additional heat. It converts compounds into "bare" gas-phase ions through charged matrix particles, also producing multiply-charged ions. Specific matrices for vMAI include 3-nitrobenzonitrile (3-NBN)[7] and coumarin,[8] known for high volatility. vMAI can ionize proteins at least as large as bovine serum albumin (66 kDa) without laser ablation[9] and has applications in analyzing various biological samples and synthetic materials. Combinations of matrix compounds have resulted in improvements of, *e.g.*, protein analyses using 3-NBN/CHCA binary matrix.[10] This is not unlike mixed solvents used in ESI[11,12,] and SAI.[13-15] The addition of heat or an inlet tube is optional but not always necessary.[16-19]

These newer discoveries in ionization have already made a significant impact on analytical measurement technologies and fundamental understanding, and highlight the evolving landscape of improvements in fields such as aerosol chemistry, drug analysis, and cancer research. There have been varied reports of the use by others in diverse fields, as exemplified by select refs.[20-40] Developments to further enhance the newer ionization methods include improvements in sampling devices, and showcasing both laser-less and laser-assisted techniques.[41,42]

It is essential to note that no ionization source or method can effectively ionize every molecular composition, as each has unique strengths and weaknesses in addressing diverse components within a complex sample. Also, not every mass analyzer and ionization source/method can be used together. Mass spectrometers are typically designed for either API or *vacuum* ionization, with vacuum ionization options more limited. A disadvantage of ionization in vacuum is the difficulty and time required to introduce samples from AP into the vacuum. Because EI and CI ionize gaseous molecules, these methods are frequently interfaced with gas chromatography (GC), where the effluent is directly introduced into a vacuum where ionization occurs. The potential of vMAI was initially hindered by the time required to insert a subliming vMAI matrix into the instrument vacuum chamber with the sample. A plate-based vacuum source that operates directly from AP and can insert a sample into vacuum in a couple of seconds, with the ease of sample insertion in ambient ionization methods, solved this issue, making vMAI an exceptional new direct ionization method. The plate source also facilitates various ionization methods (ESI, SAI, vMAI, MALDI),[10,43,44] significantly advancing sample introduction over other sources; details are included below.

Brief Summary on Sampling Devices and Methods

Improvements in ionization techniques for various sampling devices and methods from AP include using gases, heat, voltages, and collisions to enhance analyte ionization. The super-AP ionization method, developed to enhance sensitivity in ESI, operates at pressures above AP.[45] *Vacuum* ionization enables larger apertures for efficient ion transfer, reducing rim losses and improving sensitivity, as seen in MALDI-ToF mass spectrometers. Introducing liquid or solid samples directly into the vacuum near the analyzer inlet resolves many issues associated with API methods. The vMAI technique allows for the sublimation of the matrix, retaining unionized nonvolatile compounds on the vMAI probe tip and resulting in consistent gaseous analyte ion emission. Sublimation conditions can be adjusted to optimize ion production over varying timescales. The technique is suitable for a wide range of instruments and analytes. Additionally, moving the sample relative to a laser, gas, or solvent stream impact enables MS imaging, which produces detailed surface chemical information. Remote analyses can be conducted using variable-diameter sampling lines, with lasers aiding in material transfer rather than directly ionizing samples. Overall, these innovations improve the efficiency and effectiveness of MS analyses.

Liquid extraction methods, which have a long history,[46,47] have recently been combined with MS through techniques such as DESI[48] and LESA.[49] These methods typically involve a solvent to extract analytes from surfaces, which are then ionized using various techniques, including ESI and *inlet* ionization methods such as SAI or Voltage SAI (VSAI)[50] (or sometimes referred to as ESI inlet (ESII)). Additionally, other sample removal techniques (*e.g.*, for tissues and food products) have also evolved to couple with mass spectrometers, utilizing force to volatilize analytes and ionizing them afterward. A significant limitation of these analyses is the reliance on a single sampling device or ionization method, as different analytes may ionize more readily with specific sources and techniques. Multi-ionization source capabilities began with a combination of ESI and APCI, previously requiring separate sources. Current sources can be exchanged to switch between various ionization methods (*e.g.*, nanoESI, ASAP, DART). This flexibility allows for cost-effective and comprehensive analysis on a single mass spectrometer. However, certain methods, like AP-MALDI and DART, are ineffective for larger molecules due to ionization limitations and mass range restrictions. Changing ionization sources can be time-consuming and may require venting the mass spectrometer, which poses operational challenges and costs time. Ideally, procedures that allow source interchangeability without breaking the vacuum are preferable to minimize downtime and complexity.

Brief Summary of the Multiple Sources on the Same Mass Spectrometer

Mass spectrometers that switch between API methods and vacuum MALDI are costly and, when source changes are required, time-consuming. Interchangeable ionization sources and methods have been developed to enhance the utility of MS. Previously, switching between API and *vacuum* ionization required venting the instrument. Notably, one exception enables ESI and vacuum MALDI to be permanent options, although this dual-source approach is specific to a single instrument manufacturer and mass spectrometer. API methods such as ESI and APCI enable relatively quick transitions without breaking the vacuum. Intermediate-pressure ionization source interchanges, as in MALDI, are rare and still typically costly, and they typically require breaking the vacuum.

The newer ionization methods led to a recent development in vacuum ion source technology, termed the 'plate source' which theoretically can be interfaced with any API mass spectrometer to achieve vacuum (v) MALDI at intermediate pressure as well as vMAI in which abundant analyte ions from volatile and nonvolatile, high- and low-mass compounds are produced through a matrix sublimation process directly operated from AP through an innovative valve plate interlocks.[10,43,44] Advantages of this source include the speed and simplicity of analysis, cleanliness, relatively low cost, ionization from filter paper or microscope glass-slide substrates, and comparable sensitivity to API and vacuum MALDI. It was shown that this relatively simple source plate is multi-functional based on replacement operations of the sliding plate. Unfortunately, at this stage of development, the instrument can be vented by novice users via the source configuration.

Brief Summary of the Vacuum Chamber Assembly Device

The technology pertains to a vacuum chamber assembly for a gaseous ion/charged-particle transfer region, used in conjunction with analyzers such as mass spectrometers and related instruments. The term "charged particles" refers to gas-phase droplets, particles, or clusters, while "ions" are bare analyte molecules in the gas phase with a certain number of charges. The vacuum chamber assembly device typically features multiple ports (3-6) with one being the port that interfaces with the mass spectrometer, and others that facilitate the transfer of ions and charged particles from AP or intermediate pressure vacuum to a sub-AP region within the analyzer. The Ports enable various ionization methods (*e.g.*, AP ionization [API]) and enhance ion generation and transfer. The use of different sources, methods, and sampling devices and strategies is typically as seamless as a mouse click, but can be adjusted if needed (not a fixed configuration). The vacuum chamber can also be viewed as a cleaning device. It

improves the cleanliness of sources and methods that are more commonly “dirty” in operation. Easy switching of components, for example, by blocking off conduits associated with a port/source.

2. Methods

More Details to Multiport Operating Features

Alternate inlet tubes can be attached to other ports, which are usually blocked, but can be switched open for optimal air-low. For simultaneous operation of both tubes, a compromise on sensitivity may be necessary, often requiring smaller diameters or longer tubes. A key feature is the ability to change tubes without venting by using a probe rod or a shutter device controlled from AP. Low air-flow inlets can enhance ionization in adjacent ports, such as an adjacent vacuum ionization source. The multi-port vacuum chamber assembly simplifies production while maintaining low-cost, size, and weight, enabling maximum utility in analysis without requiring the selection of an ionization source. The technology is compatible with various API mass spectrometers, enabling rapid or simultaneous access to multiple ionization methods, including API (ESI and APCI, for example), inlet ionization (SAI and MAI, for example), and vacuum ionization techniques (vMALDI and vMAI, for example). Users can easily switch between these methods with a software click, often without changing any physical components.

Multiple ports may be used for various vMAI probes, vMAI plates, or SAI surface-sampling devices, among other applications, to enhance ionization, transfer gas-phase ions, or aid matrix removal from charged particles. These ports can also serve as valves to prevent high pressure from affecting the analyzer device during maintenance. Probes may provide movable collision surfaces and may be subject to applied voltages and heat, both conventional means altering ionization outcomes. A port may feature a ball valve that allows a probe to pass through when open. The probe can be positioned within the port for optimal ionization. In this setup, a first tube connects the port to the analyzer device via a ball valve, preventing gas flow when the valve is closed. The second tube connects to a fitting that seals the probe, maintaining operational pressure during use. The probe may carry a sample or incorporate capillary tubing to enhance ionization through gas flow. Additional features might include laser-beam transmission into the chamber, while gas flow at the port can aid ionization and ensure efficient transfer of charged particles. This setup maximizes the functioning of the vacuum system with minimal gas load.

One port of the multi-port vacuum chamber assembly can interface with a plate device, which features a flat surface on the high-pressure side and is linked to a low-pressure region

within the vacuum conduit. By placing a flat plate over this surface, the higher-pressure region is sealed, preventing gas from entering the conduit. This setup allows the plate to function as an ion source and, when properly aligned, acts as a valve plate to block gas flow. The valve plate typically has no channels, while a spacer plate may include multiple channels or grooves. When the spacer plate covers the conduit, it blocks gas flow unless aligned with the opening. The plate assembly, comprising the spacer plate and a sample holder, can slide over the port surface, maintaining vacuum integrity while exposing the channels to the sub-AP of the vacuum chamber. Samples can be placed on the sample plate to align with the spacer plate channels, allowing for controlled exposure to the vacuum. Alternatively, a paper substrate, such as filter paper, can be used for sample collection, providing stable ion signals across various biological materials. This arrangement is adaptable to multiple samples, enabling efficient analysis with advanced techniques such as MS/MS, thereby enhancing specificity and sensitivity.

Selecting the appropriate paper and backing (*e.g.*, polymer film) is crucial for controlling gas flow and crystallization, both of which affect reproducibility and quantification. Plastic sheets with sticky sides and holes can act as spacers by sandwiching the paper sample. Polystyrene is a good choice because it has no ionizable background making it suitable for use in sampling devices for illicit drug detection. Filter papers, including those from Whatman, are versatile but should be matched to specific applications; for example, dilute nitric acid may be incompatible with certain papers. Uranyl nitrate can be analyzed using >2% nitric acid in solvents like acetone, water, or ethanol. High-end applications may benefit from specialized life sciences filter papers, particularly for accurate mass detection in protein analysis. A plate separating the sample from the port is called a spacer plate, and the design supports customization, including automation. In vMAI, ionization occurs when the matrix: analyte sample is exposed to sub-AP conditions. Examples of various introduction approaches are depicted in **Scheme S3**. A laser beam can also ionize samples via MALDI or LSI. In MALDI mode, a lens focuses the beam onto the sample, and smaller apertures can maintain vacuum integrity. To extend sampling, the laser can strike off-center, allowing access to new surfaces via rotation or plate movement. Glass slides can facilitate transmission-geometry laser ablation, requiring fewer laser shots to achieve good analyte abundance. With adequate laser power, ionization can occur without a matrix as in laser desorption/ionization (LDI). Applying a voltage (20 V to 1000 V) to the substrate or spacer plate increases ion abundance, particularly for larger molecules such as proteins, whereas smaller molecules may fragment “in-source”. For direct MS, mass-selected MS/MS improves sensitivity and specificity. The multi-port vacuum chamber may include features for focusing and ion extraction to facilitate the transfer of charged species into the analyzer.

In practice, the most accessible port on the analyzer device is used for ionization sources and methods that require more physical space, often located at the second end of the conduit, opposite the mass analyzer entrance port. Sometimes, a port's functionality depends on another; for instance, vMALDI requires the laser introduction port to be positioned opposite the vMALDI port. This design allows easy adaptation to different configurations, and extra blank ports enable future exploration of new setups (flexibility). The multi-port vacuum chamber assembly offers several advantages for mass spectrometers and related instruments, enabling users to select from a wider range of ionization methods at a lower cost than traditional methods. This arrangement facilitates repurposing instruments for various applications, including newer ionization technologies, such as vMAI, MAI, SAI, VSAI, and vLSI. Key benefits include: no need for additional pumping stages, rapid sample exposure (2-3 seconds), capability for sequential mass spectral acquisitions, compatibility with existing mass spectrometers, low contamination risk, ease of cleaning, low cost, and support for multiple ionization methods like APCI, APPI, ESI, and MALDI without instrument venting. Additionally, reflection geometry laser alignment is essential for samples that the laser should not penetrate through, making it suitable for various applications, including *in vivo* diagnostics and imaging.

More Details to Surface Pen Based on the Concept of the Surface Reader Device

The multiport vacuum chamber supports advanced surface analysis (**Scheme S4**) by enabling multiple expansions of the Surface Reader Pen (or simply Surface Pen), leveraging prior surface analysis knowledge and successful implementation on primarily Thermo mass spectrometers. This hardware expansion of the Surface Pen concept, first publicly disclosed at ASMS in 2016,[51] is based on the concept of the Surface Reader, which was first publicly disclosed at ASMS in 2011.[52,53] Several aspects are illustrated in photos and in **Movie S5** and **S8-12**, and are briefly described below. Remote analyses with and without a laser, from solid or liquid samples, are now readily available, unlike some of the previous demonstrations.[53,54-57] These different ionization sources, methods, and sampling strategies are now nearly instantaneously available individually and as a bulk improvement. In a brief selection, Surface Reader analyses based on **Variations I** and **II** were operated on a Thermo Q-Exactive Focus mass spectrometer, and **Variations III** and **IV** on a Waters SYNAPT G2S. This serves as excellent proof that the multiport vacuum chamber system greatly expands the utility of any mass spectrometer (*e.g.*, compare with refs. 58,59). **Variations II** and **III** rely heavily on 3D printing, allowing faster, lower-cost prototyping and enabling rapid iteration before finalizing a production design. **Variations I**

and **IV** developments are geared toward rapid use of existing equipment for more immediate use in the healthcare sector.

In greater detail, the development of Variation I constitutes a minimalist probe. Development of **Variation II**, is a more advanced, self-contained Surface Pen with dual solvent reservoirs (one for water and one for an additional solvent). It features built-in micro-pumps for precise droplet formation on the sample surface before the return line transfers the analyte to the mass spectrometer. This represents a significant step toward portable, independent operation. More recent developments include the current internal mechanisms and body design, as well as the Surface Pen body **2B**, which is smaller, lighter, and ergonomically optimized for single-handed use. **Variation III** is a functional, snap-fit two-piece Surface Pen assembly with 375 μm cutouts for two fused-silica lines: one connected to a syringe pump delivering solvent to the surface, and the other serving as the return line to the mass spectrometer. **Variation IV**, covers the concept of re-functioning ("re-purposing"), disposable FDA-approved equipment (obtained from both Corewell Health, Binsons, and other local and online medical health care providers) as a transport mechanism between the surface to be sampled and a mass spectrometer for mass analyses. As in other probes, the solvent needs to be deposited onto a surface one way or another, continuous or discontinuous, and one end of the conduit is exposed to AP, and the other is "connected" through fluid communication with the sub-AP of the mass spectrometer for delivery to the inlet tube, preferably heated. The researcher and operator is a first-year undergraduate student who, within <5 minutes, gets the new probe device to work on their first try (**Variation IV**).

3. Materials Section

Mouse brain tissue: Briefly, mice were anesthetized with isoflurane overdose followed by perfusion of 20 ml of ice-cold phosphate-buffered saline, as previously described.[60] Brains were rapidly removed and frozen. Mouse brain tissue sections were cut with a cryostat (Leica) and mounted on microscopy glass slides. Tissue sections were cut to 10 or 20 μm and stored at −80 °C prior to shipment to SJU and WSU, respectively, for analysis. All animal procedures were approved by the Indiana University Bloomington Institutional Animal Use and Care Committee.

The mouse brain tissue sections were analyzed by surface SAI with and without the use of a laser. The solvent drop was either deposited onto the tissue section or a droplet junction was formed between two closely aligned tubes, as in previous procedures.[60] Depending on the solvent and solvent combination, the droplet extracts chemicals from the tissue, and the extract is transported via suction from the mass spectrometer's vacuum pumps towards the mass analyzer. Ionization occurs in the inlet tube, and depending on the solvent or solvent composition used, one

may prefer to add heat to the tube to increase the abundance of gaseous analyte ions and improve analytical performance. Depending on the mass spectrometer's polarity setting, ions are detected in positive- or negative-ion mode.

Bacteria: Briefly, the studied BSL-1 bacteria include *Escherichia coli* (*E. coli*; K12, DH5α, BL21(DE3)), *Serratia marcescens* (*S. marcescens*), *Providencia alcalifaciens* (*P. alcalifaciens*), *M. smegmatis*, *S. aureus*, *S. epidermidis*, *B. cereus*, *B. subtilis*, *S. salivarius*, and *K. aerogenes*. Organisms were obtained from ATCC and stored at -80 °C. Cultures were grown on LB agar at 37 °C for 16-36 hours depending on the organisms. Bacteria were gently scraped from the agar surface and placed in a microcentrifuge tube. Formic acid (88%) was used to lyse the bacteria prior to dilution with 1:1 ethanol:water before 1 µL of this solution was applied to four positions on Whatman Grade 52 filter paper, with one position reserved for a blank.

Safety: All procedures were conducted in accordance with BSL-1 guidelines per CDC and NIH recommendations. Personal protective equipment (gloves, safety glasses, lab coats) was worn during experiments in a BSL-1 facility. Agar plates were stored at -4 °C after wrapping in paraffin.

Mass Spectral Acquisition: The filter paper is placed in the plate source cartridge, creating an airtight seal with the mass spectrometer vacuum. The spacer plate sequentially exposes channels, enabling ionization of the samples. This process typically generates four replicate mass spectra and one blank in under two minutes. For complex bacterial samples, the acquisition time is set to 1.5 to 2 minutes for each to improve the ion abundance.

4. Applications

Applications at the point of need include analyses in biological, clinical, forensic, and agricultural fields, as well as drug detection, environmental sampling, and microbial identification, among others. The technology enables advanced sample analysis, allowing precise, timely measurements with or without additional energy inputs and with greater ease. It combines traditional and innovative ionization techniques to enable effective molecular analysis across diverse applications on a single mass spectrometer. Note that the combination of IMS with direct ionization is advantageous for complex samples for which liquid chromatography (LC) is impractical. Mass spectra can provide analytically useful sensitivity, even at ambient temperatures, for inlet or vacuum ionization.

5. Supporting Data

Scheme S1. Photographs of prototypes of a 3-port vacuum chamber assembly: (**A**) port is used for API and inlet ionization sources/methods, sampling and auxiliary devices, and a port for vMAI operation and auxiliary devices using a probe device having an automated ball valve. The photograph in (**B**) shows a third port with a tube that allows gas to flow into the conduit, enhancing vMAI ionization of the sample on the probe. Both (**A**) and **B** (**1**) are installed on a Thermo Fisher Q-Exactive Focus and **B** (**2**) on a Waters SYNAPT G2S mass spectrometer. (**A**) is configured with a probe device and (**B**) with plate source devices. **B** (**1**) is configured for transmission geometry vMALDI, and **B** (**2**) for vMAI using filter paper as a sample substrate.

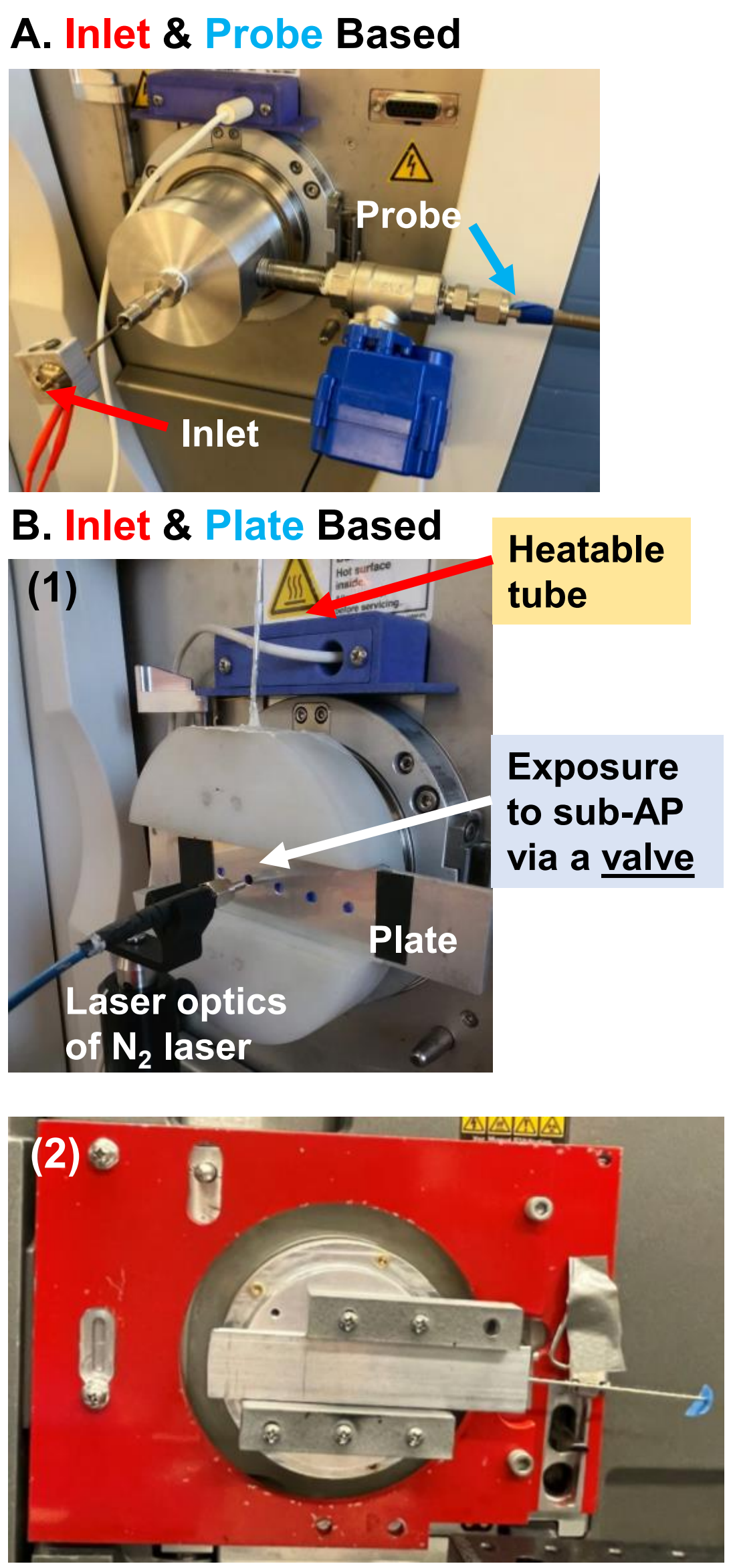

Scheme S2. Photographs of prototypes of 4-port vacuum chamber assembly devices installed on (**A**) Thermo Fisher Q-Exactive Focus and (**B**) Waters SYNAPT G2S instruments, with inset display bottom left; here, both are using a probe device and an inlet that can be heated. The Thermo 4-port configuration has a 2nd heatable inlet tube, whereas the Waters configuration uses a plate source as the 4th port. (**B**) Laser-based 4-port configuration, here with the laser in reflection geometry, with the sample being ablated from the probe tip and the laser beam transmitted through a glass plate [here (**1**) AP inlet, (**2**) probe source inlet, (**3**) plate source inlet]. A movie (**Movie S2**) is included in the analysis of oxycodone, based on the reflection-geometry operation shown in (**B**). These are only a few of the numerous analytically useful combinations of multiport arrangements.

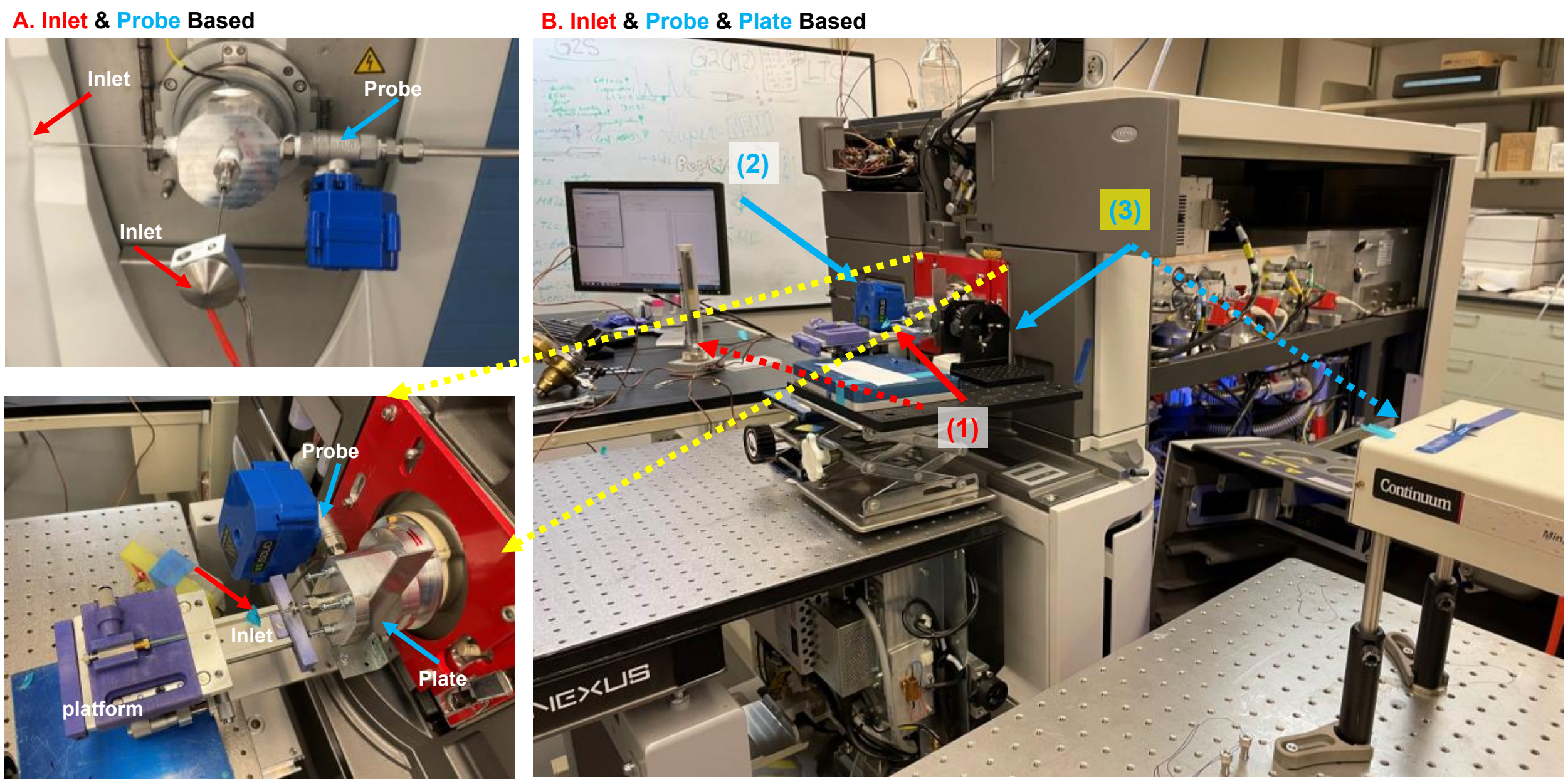

Scheme S3. Photographs of various sample supports and sampling devices used for the various ionization techniques (vMALDI, vMAI, ESI, and SAI). (**A**) Spacer plates with and without filter paper. (**B**) Spacer plate with filter paper in guide rails of flange separating AP and the instrument vacuum. (**C1**) Well plated and accessories. (**C2**) Towards 2D spacer plate movement. (**D**) Blood spot sampling (**E1-5**) Early versions of the plate source and spacer plates).

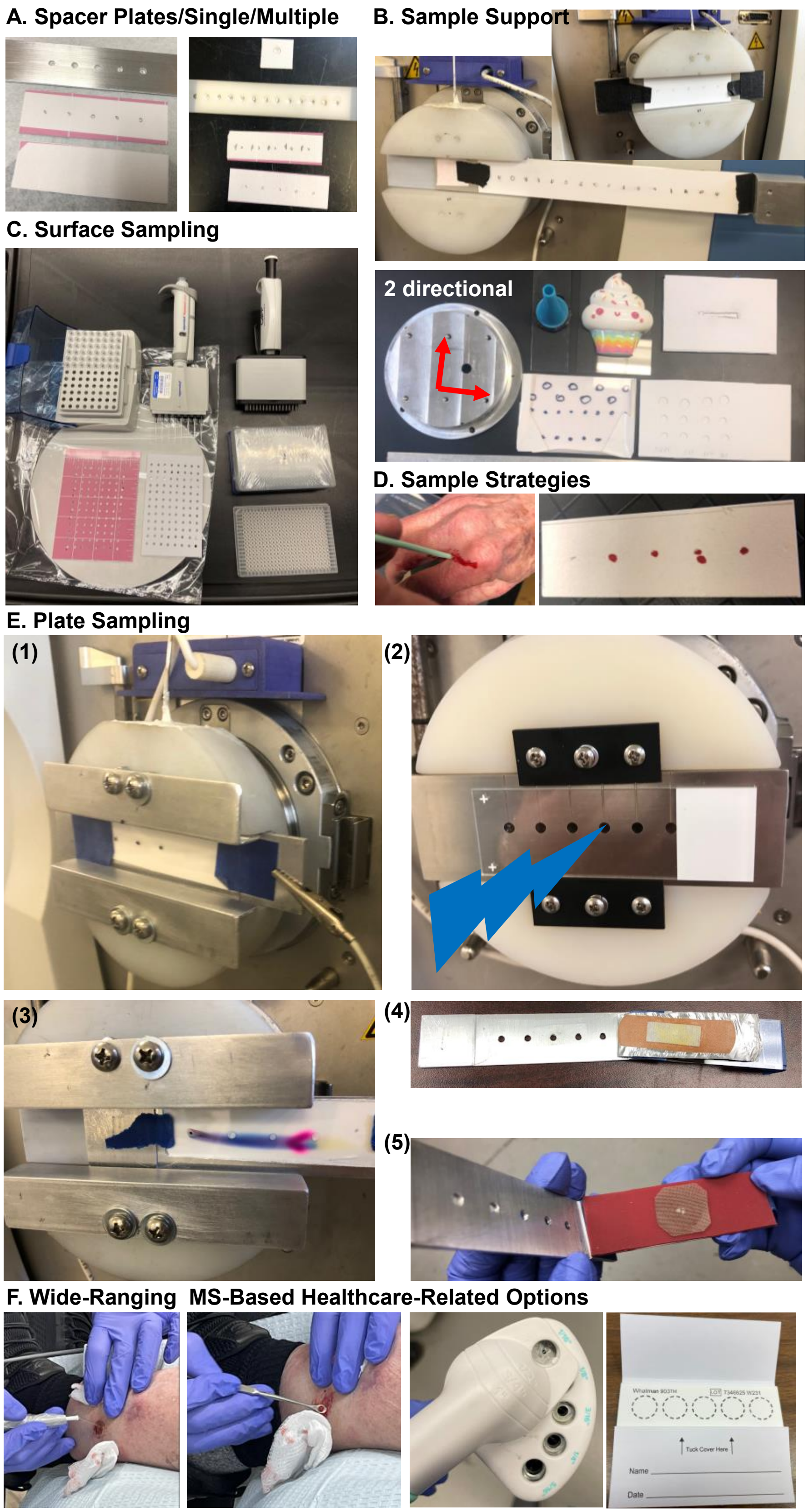
A. Spacer Plates/Single/Multiple
B. Sample Support
2 directional
C. Surface Sampling
D. Sample Strategies
E. Plate Sampling
(1)
(2)
(3)
(4)
(5)
F. Wide-Ranging MS-Based Healthcare-Related Options
Name
Date

Scheme S4: Variations of the Surface Pen using the concept of the SAI Surface Reader. **Note 1**: ***Variation I***: In **Variation I**, a solvent is either pumped onto the surface or a liquid dropped onto a surface and aspirated by the vacuum of the mass spectrometer into the inlet of the mass spectrometer, where ionization occurs on the fly, and depending on the solvent used, heat is preferred or necessary. This concept is most closely related to the early work presented by the McEwen and Trimpin groups.[4,5,13-16] Importantly, this was done on the 4-port system, which replaced the commercial ESI source on a Thermo mass spectrometer. ***Variation II***: **Variation II** self contains solvent vials, micropumps, mixing chamber, isolation valves, and tip. Electronics board and computer control are external. Body shown alongside **Surface Pen 2A** fitted with micropumps for testing. **The Surface Pen 2B, shown with cover,** is smaller, has thinner walls, is lighter, and is more ergonomic. ***Variation III***: In **Surface Pen III**, a solvent is either pumped onto the surface or a liquid dropped onto a surface and aspirated by the vacuum of the mass spectrometer into the inlet of the mass spectrometer, where ionization occurs on the fly, and depending on the solvent used, heat is preferred or necessary. Importantly, this is performed using a mass spectrometer that initially performed poorly with SAI because the Waters Z-spray source requires an inlet tube retrofit.[14,15] ***Variation IV***: In **Surface Pen IV**, similar to the other Pens. Analyte dissolved in a solvent or solvent mixture is drawn by the vacuum of the mass spectrometer into the inlet of the mass spectrometer where ionization occurs on the fly and ionization occurs in the inlet tube, which may be heated, depending on the solvent used. Importantly, this is done with a disposable healthcare product repurposed that costs in the order of ~$2. Further, the research involved a first-year undergraduate student and on a mass spectrometer (SYNAPT series, Waters) retrofitted with an inlet tube.[14,15] **Note 2**: ***for operation of Variation II***: (**1**) Unscrew caps A and B to fill with desired solvent. Cap A serves as the reservoir for water that is deposited onto the sample surface. Cap B is the additional solvent reservoir. This can be filled with water to clean the return line, left empty to purge the return line (air), or filled with methanol, ethanol, isopropyl alcohol, *etc.*, to mix with the collected surface sample. (**2**) C is the return line to the MS. It is downstream of the isolation valve, which can be opened and closed. Movies (**Movie S9-S12**) are included.

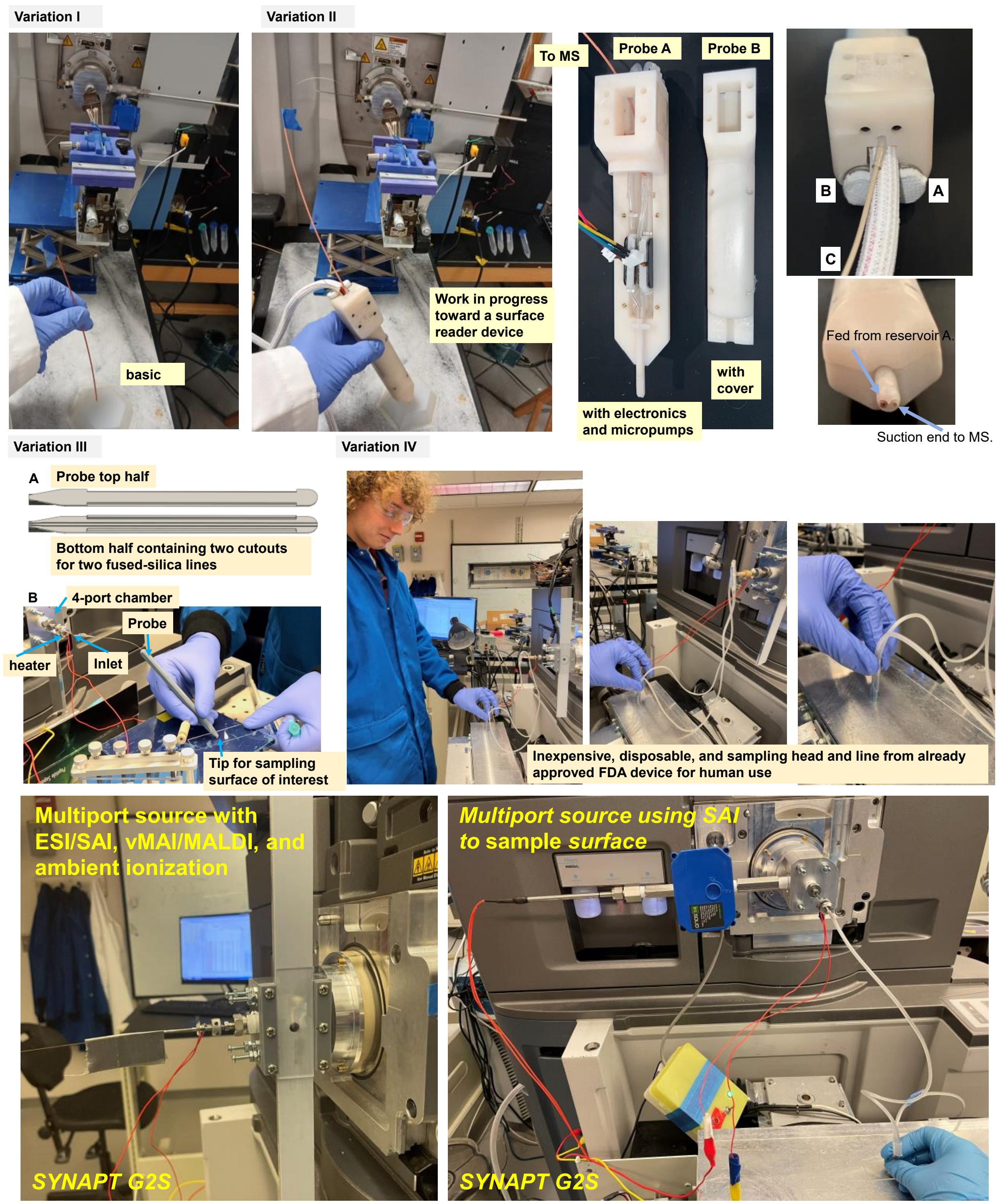
Variation I
Variation II
To MS
Probe A
Probe B
B
A
C
basic
Work in progress toward a surface reader device
Fed from reservoir A.
with cover
with electronics and micropumps
Suction end to MS.
Variation III
Variation IV
A
Probe top half
Bottom half containing two cutouts for two fused-silica lines
B
4-port chamber
Probe
heater
Inlet
Tip for sampling surface of interest
Inexpensive, disposable, and sampling head and line from already approved FDA device for human use
Multiport source with ESI/SAI, vMAI/MALDI, and ambient ionization
SYNAPT G2S
Multiport source using SAI to sample surface
SYNAPT G2S

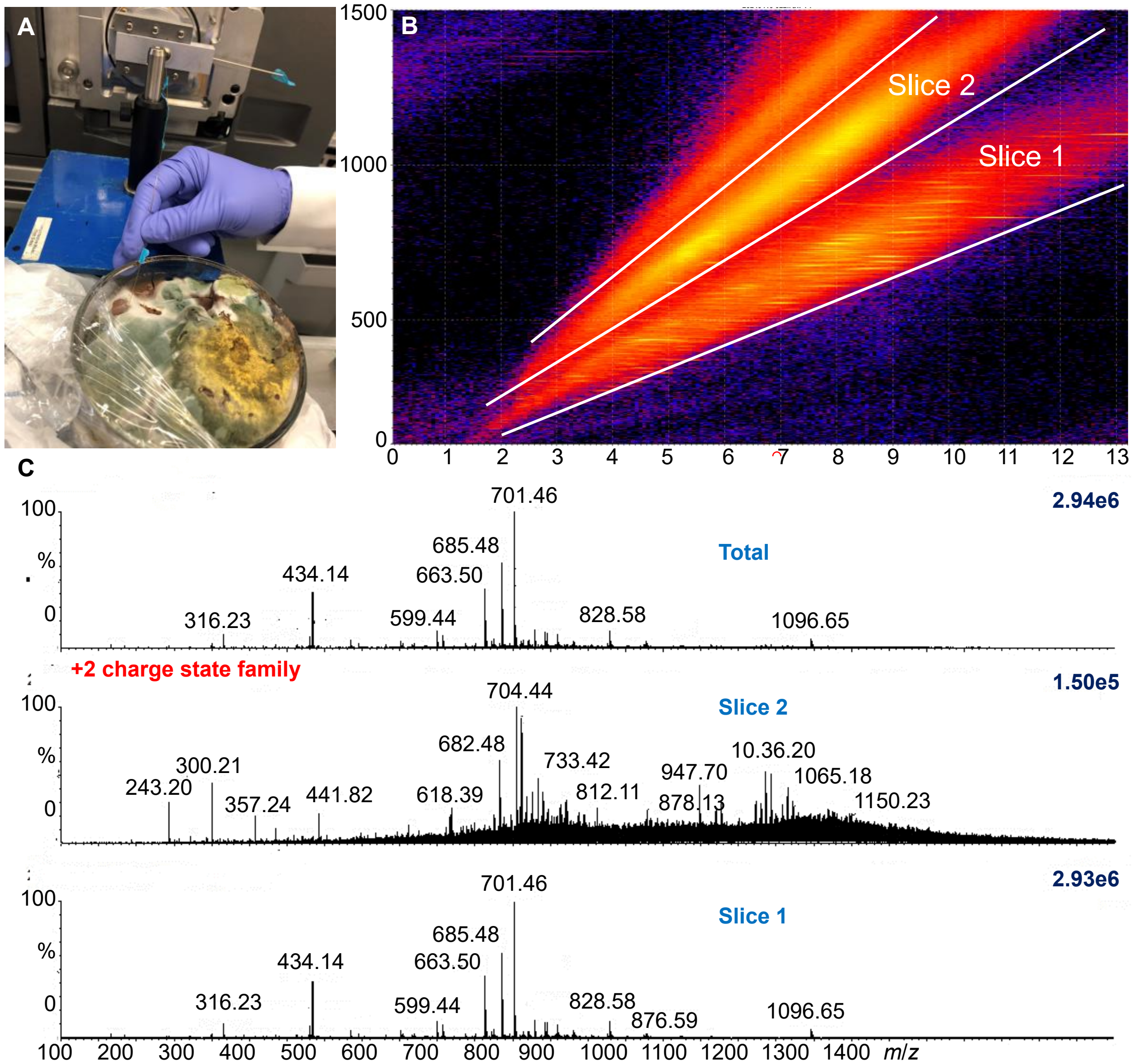

Figure S1: Surface SAI of a rotting food sample using IMS-MS acquisitions: (**A**) photograph in which a drop of solvent was placed onto the surface of fungal mold. Sliding the inlet tube plate of the plate source brings the droplet on the surface, at a remote distance, into fluid communication with the mass spectrometer vacuum. The liquid is drawn into the inlet tube, where ionization occurs; (B) 2-D plot of nearly instantaneously separated ions by IMS, followed by TOF-MS and ion detection. 2-D plot display of ion-mobility separation (drift time) of charges detected using Surface SAI-IMS-MS on fungal mold. Slice 1 constitutes the typical singly-charged ions and slice 2 higher charge states, mainly +2; (**C**) the total and extracted mass spectra of the fungal mold that successfully ionized in situ ('on the fly'). No heat was applied to the inlet tube when using volatile solvents such as methanol or isopropyl alcohol. Data acquired on a 3-port vacuum chamber assembly retrofitted on a Waters SYNAPT G2S mass spectrometer.

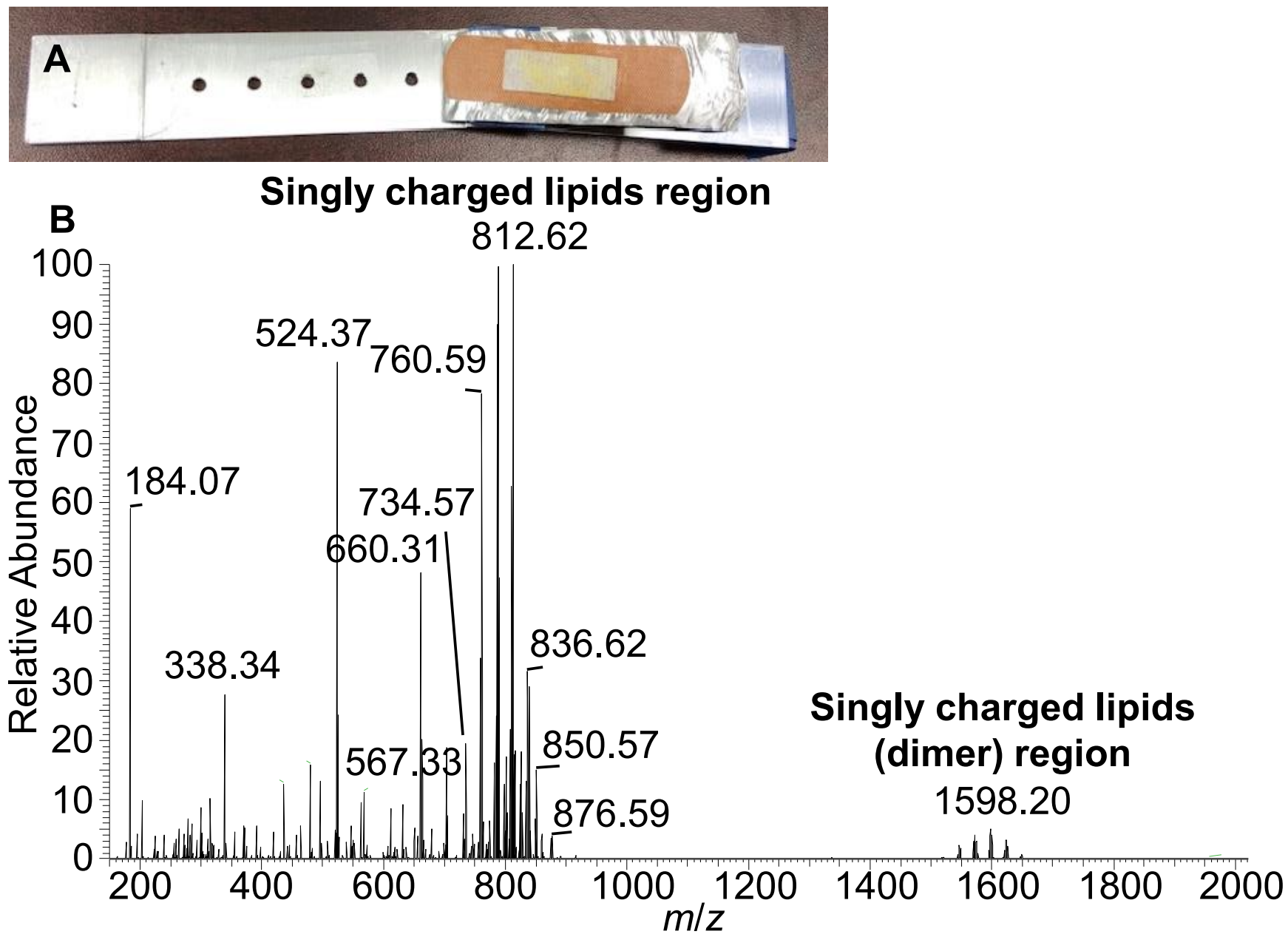

Figure S2: (**A**) Samples were spotted on a band-aid adhesive strip, and 3-NBN matrix solution was added, dried. and assembled such that the gauze side of the adhesive strip was in contact with the openings of the spacer plate. The vacuum integrity of the mass spectrometer was ensured using a self-adhesive metal foil over the adhesive strip to prevent too much air-flow through the gause. (**B**) vMAI mass spectra of blood spot using the approach in (A).

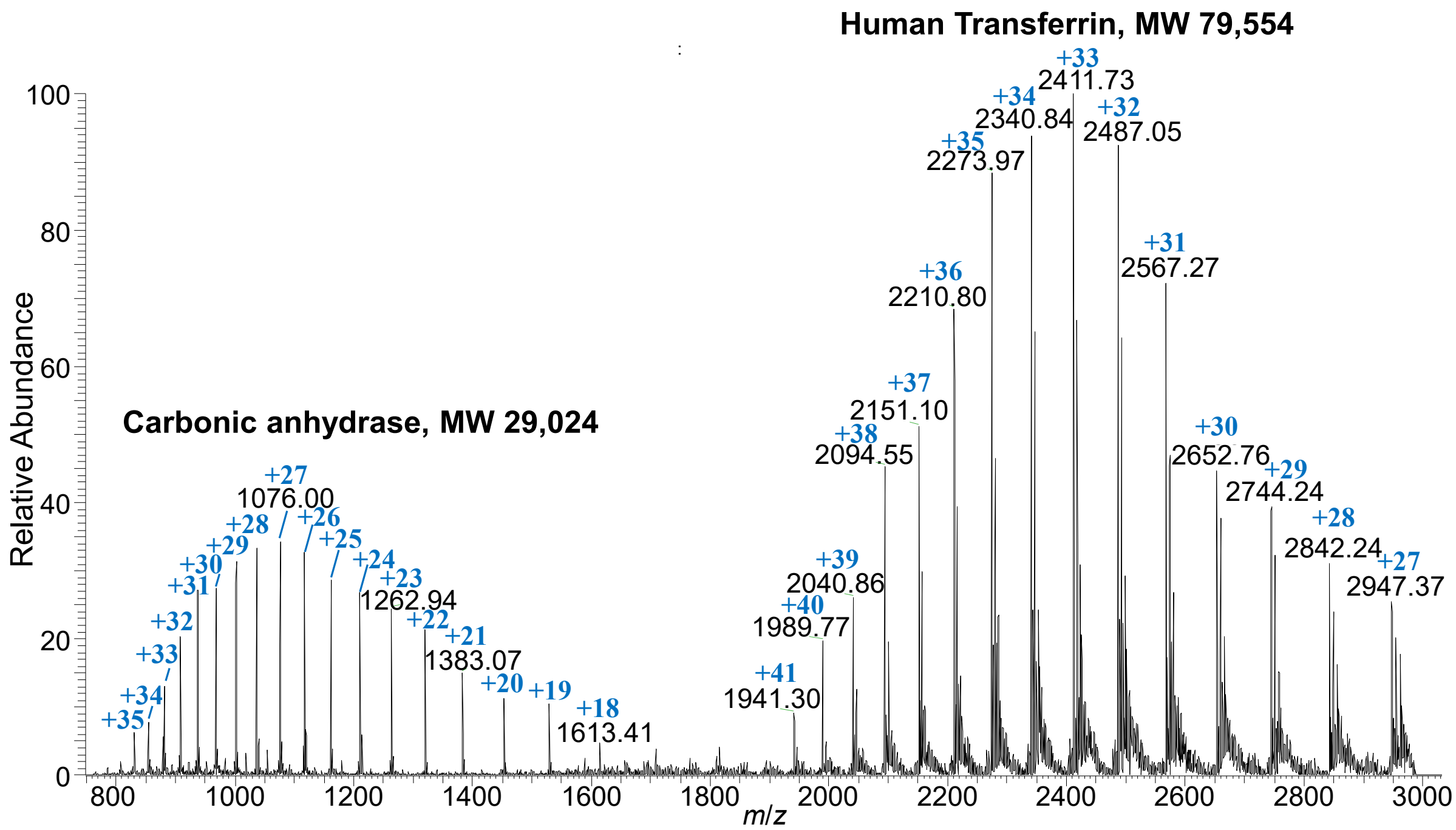

Figure S3: A representative example of an ESI mass spectrum of a mixture of 10 micromolar each of carbonic anhydrase (MW *ca.* 29,025) and human transferrin (MW *ca.* 79,550) obtained using the prototype 4-port vacuum assembly device (**Scheme S2A**) on Thermo Q-Exactive Focus mass spectrometer. The heater device retrofitted onto the inlet tube was at *ca.* 300 °C.

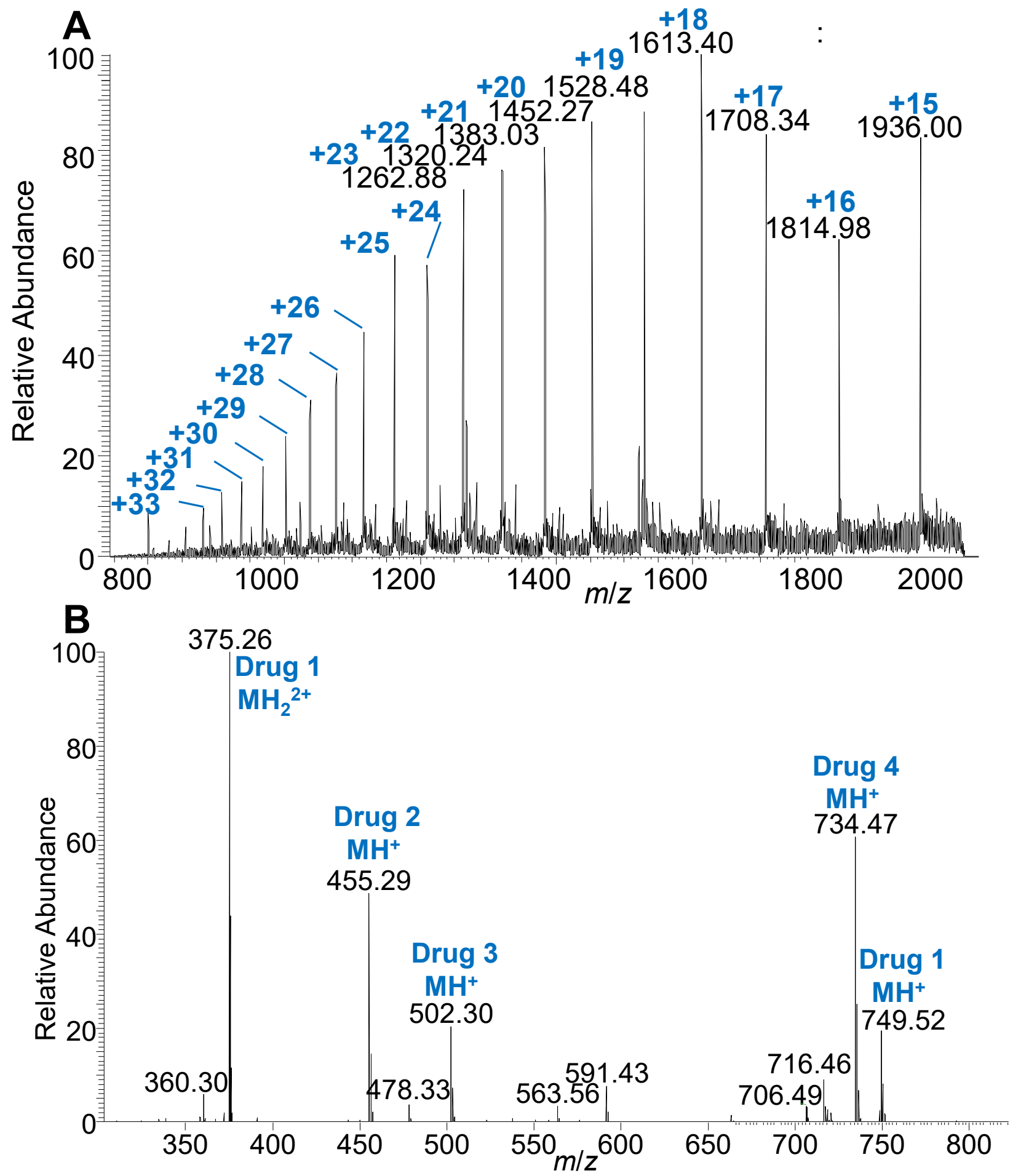

Figure S4: vMAI mass spectra of (**A**) carbonic anhydrase in 3-NBN/CHCA matrix[10] solution added to the end of a probe tip, dried and inserted into vacuum, and (**B**) 1 pmol per drug mixture (Drug 1 azithromycin, Drug 2 verapamil, Drug 3 fexofenadine, Drug 4 erythromycin) in 100% DMSO. One (1 µL) of the sample was added in solution to 3-NBN matrix solution (1 µL) and dried before insertion into the vacuum of the mass spectrometer. Data acquired on the 4-port vacuum chamber assembly device with the vMAI probe source (**Scheme S2A**) on a Thermo Q-Exactive instrument.

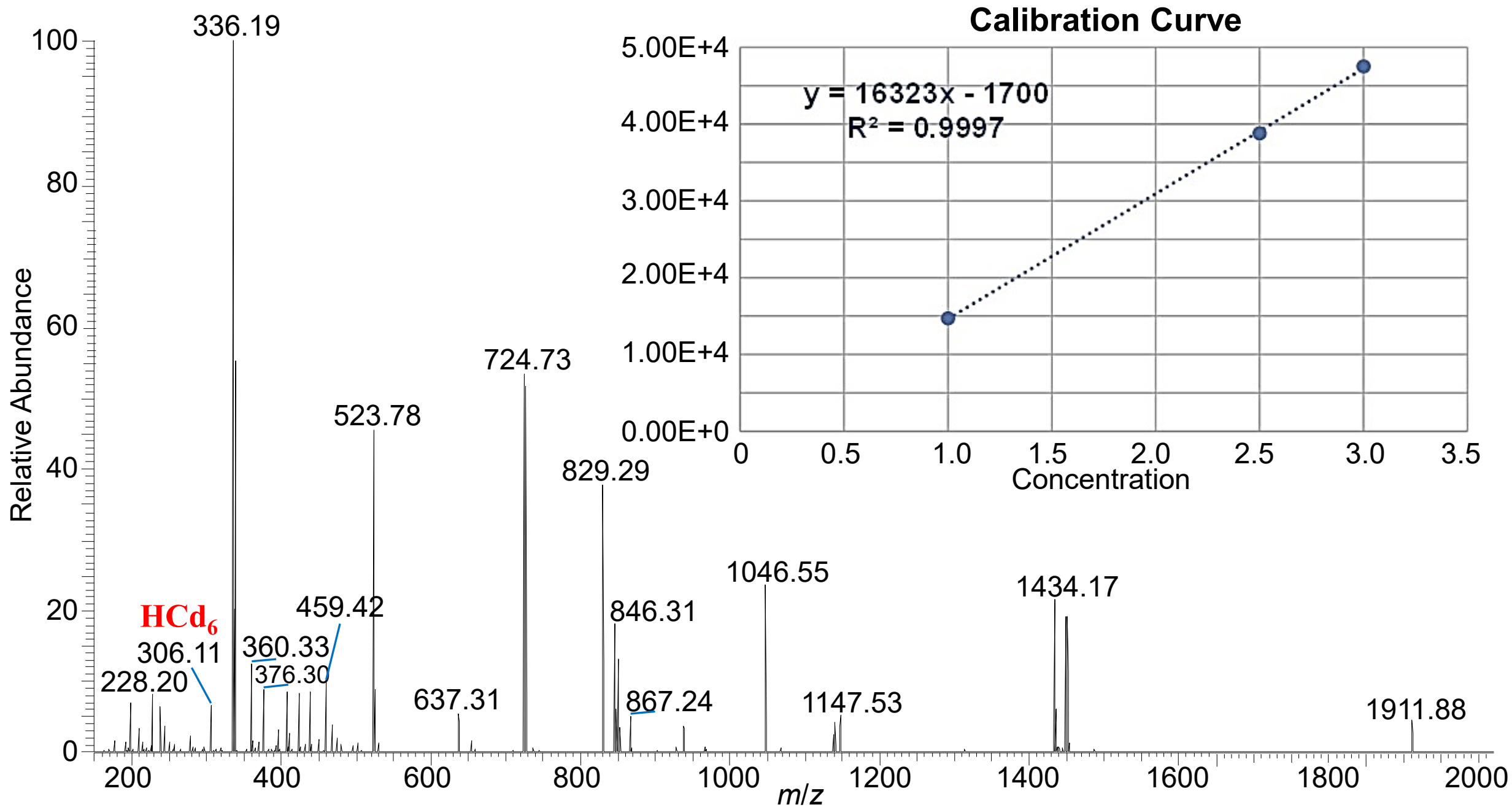

Figure S5: Quantification of hydrocodone spiked into a mixture (hydroxychloroquine, angiotensin II, vancomycin, maltopentaose, bovine insulin) using hydrocodone-d6 as an internal standard. The inset shows a calibration curve using 1µL of micromolar solutions per analysis. Data acquired using SAI on a 4-Port source with the additional port used for directed gas flow on a Thermo Q-Exactive Focus mass spectrometer.

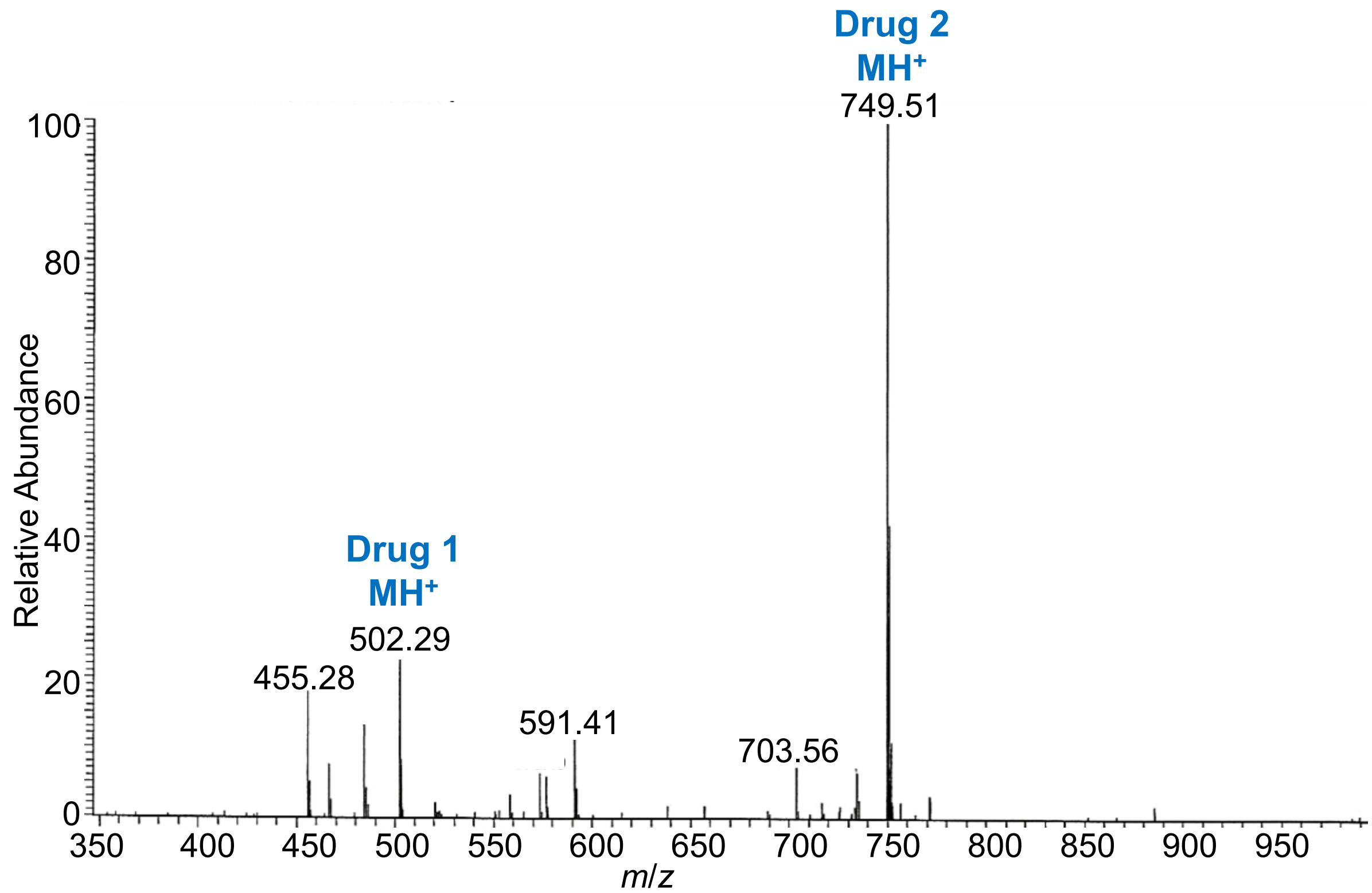

Figure S6: A mass spectrum of the drugs fexofenadine (*m/z* 502) and azithromycin (*m/z* 749) using a proof-of-principle multiport vacuum chamber assembly schematically shown in **Scheme S1B** to obtain the transmission geometry vacuum MALDI spectrum using 2,5-DHAP[5] as a matrix on a Thermo Q-Exactive Focus mass spectrometer. A nitrogen laser with fiber optics and a focusing lens was employed.

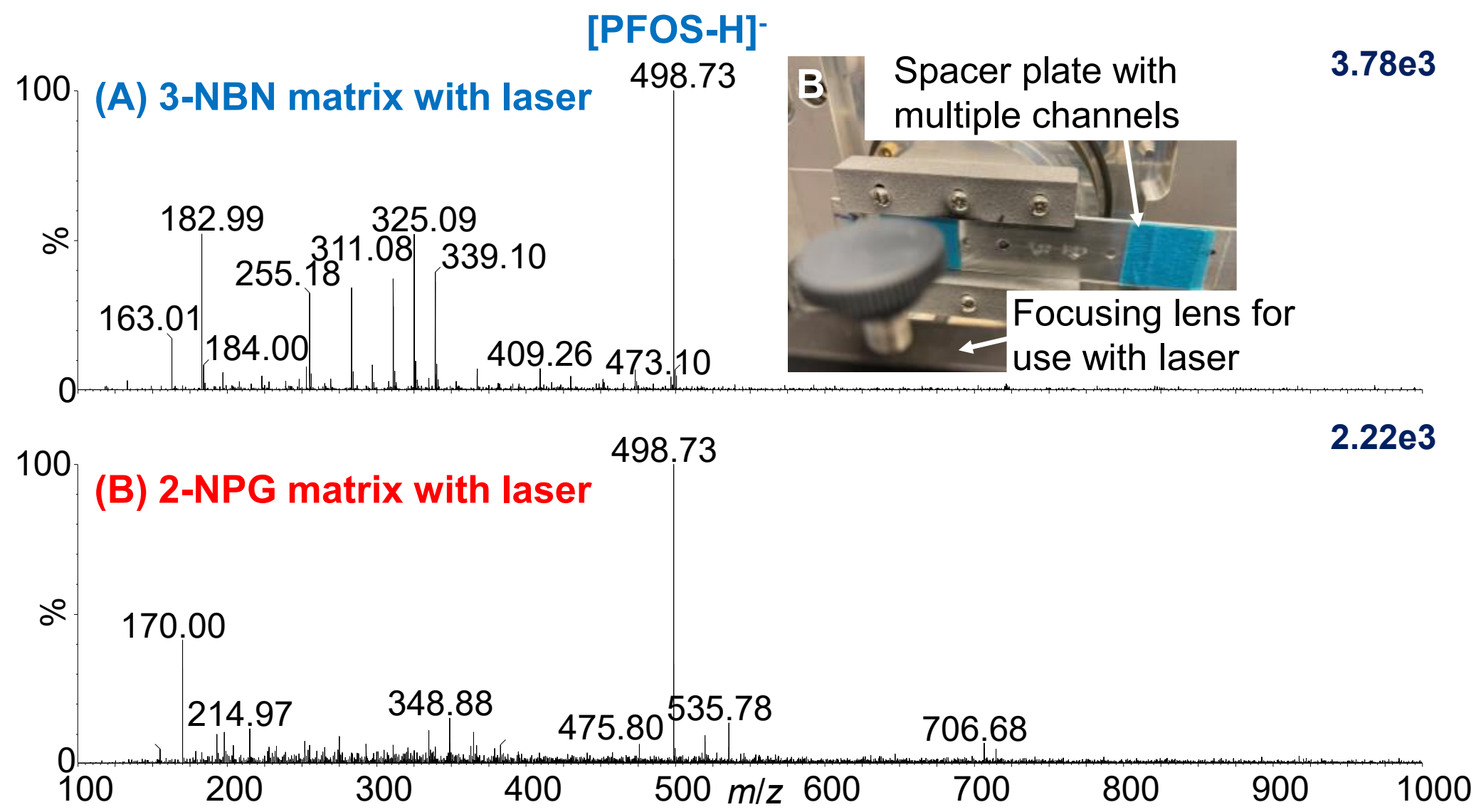

Figure S7: Vacuum MALDI of perfluorooctanesulfonic acid (PFOS) in negative mode on the 3-port vacuum chamber assembly using matrices (**A**) 3-NBN[7] and (**B**) 2-NPG,[6] respectively, using a Nd:YAG laser (355 nm) to ablate the sample in transmission geometry through a glass microscope slide into a 3-port vacuum chamber assembly (the inlet tube for additional gas flow is hidden behind the spacer plate in the photo above). The data were acquired on a Waters SYNAPT G2S mass spectrometer.

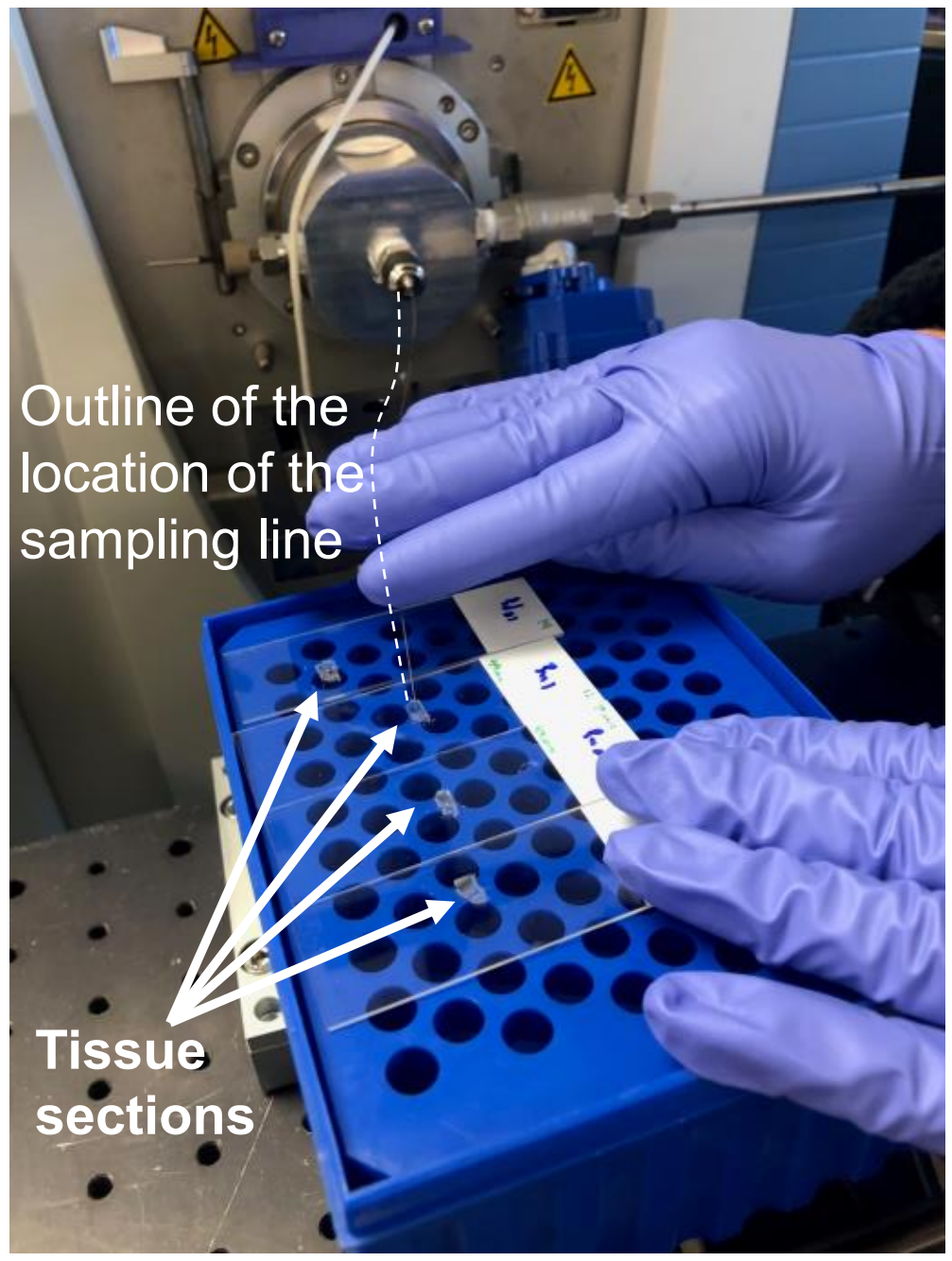

Figure S8: Analyses of four consecutive tissue surfaces demonstrating acquisitions in rapid succession by surface SAI using 4-port vacuum chamber assembly on Thermo Q-Exactive Focus instrument.

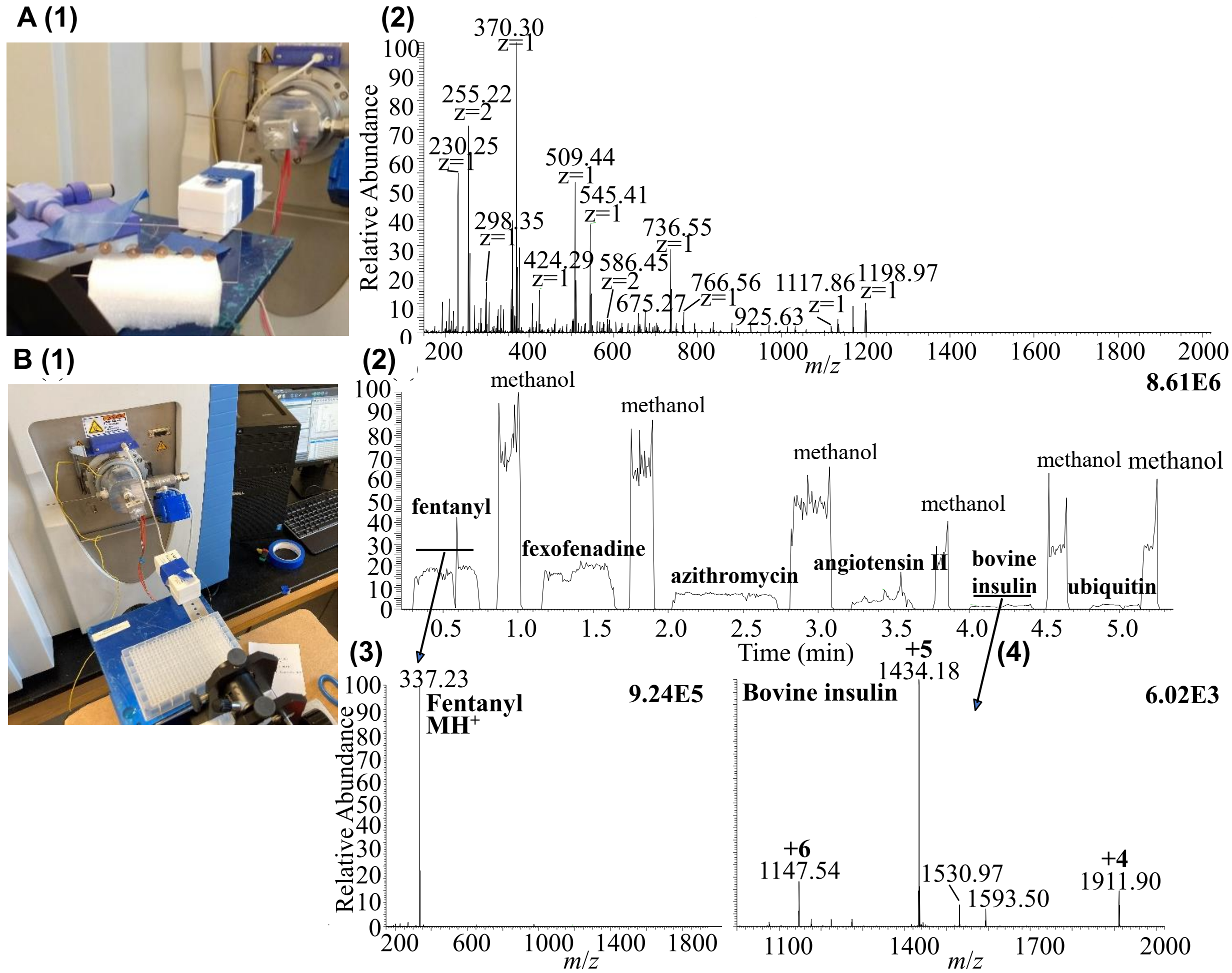

Figure S9: Experimental setups for remote sampling using SAI interfaced to the 4-port vacuum chamber assembly. Liquid junction (**A**) blood spot analyses using MeOH as matrix. (**1**) photographs and (**2**) mass spectra acquired from remote surface. (**B**) Sample in solution drawn from well plates, here 96: (**1**) photograph of setup, (**2**) total ion chronogram, (**3**) mass spectra of fentanyl, (**4**) bovine insulin. The data was acquired on 4-port vacuum chamber assembly on a Thermo Q-Exactive Focus instrument.

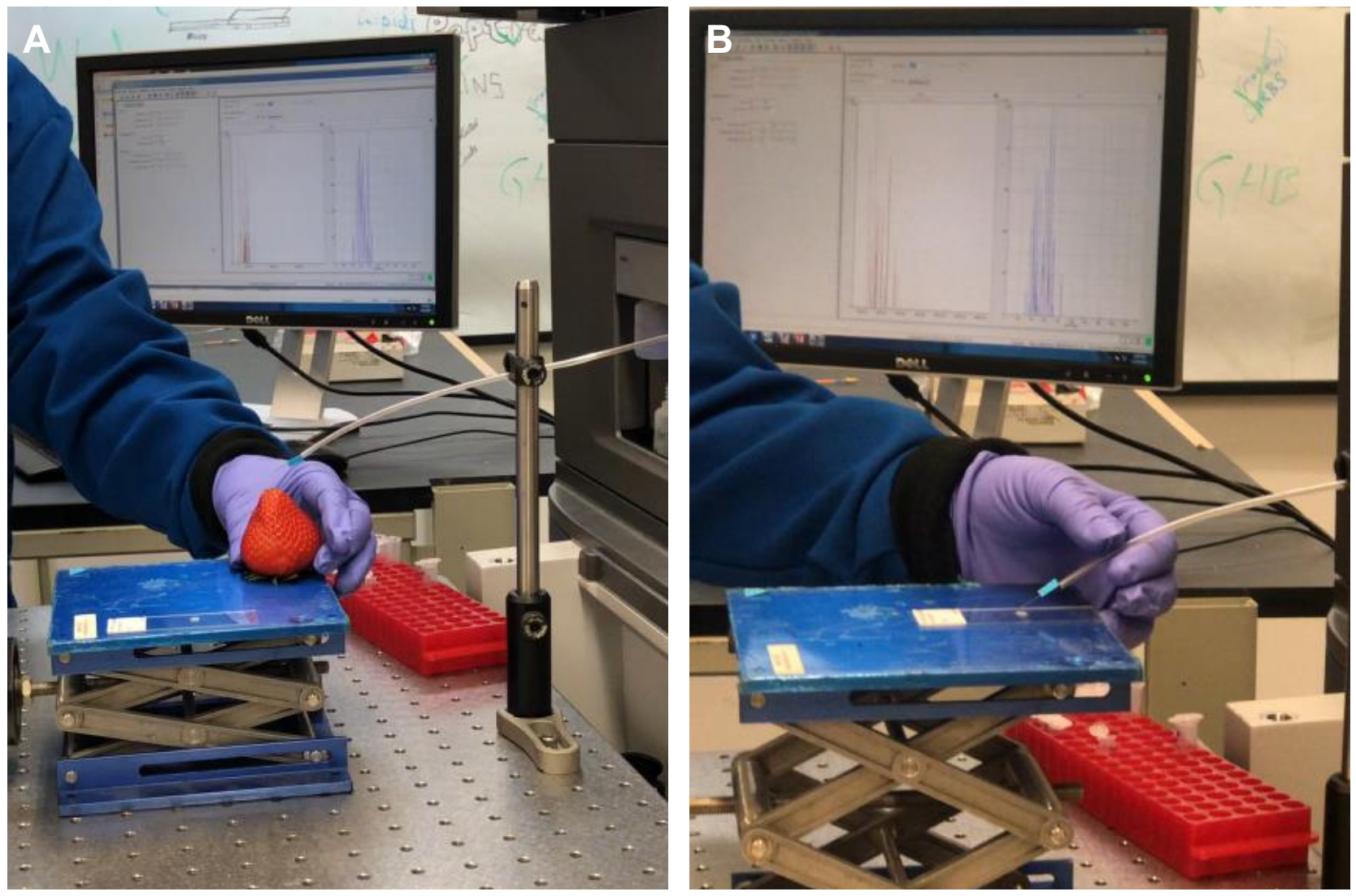

Figure S10: Illustrations of surface SAI with (**A**) a drop of water being transferred from a strawberry into the inlet tube of a 4-port vacuum chamber for ionization, and (**B**) a drop of water on a mouse brain tissue being transferred into the inlet tube. Data acquired on 4-port chamber retrofitted on a Waters SYNAPT G2S mass spectrometer. Movies **S4** and **S5** are included to demonstrate the process of surface sampling based on SAI. Compare with previous work. [1]

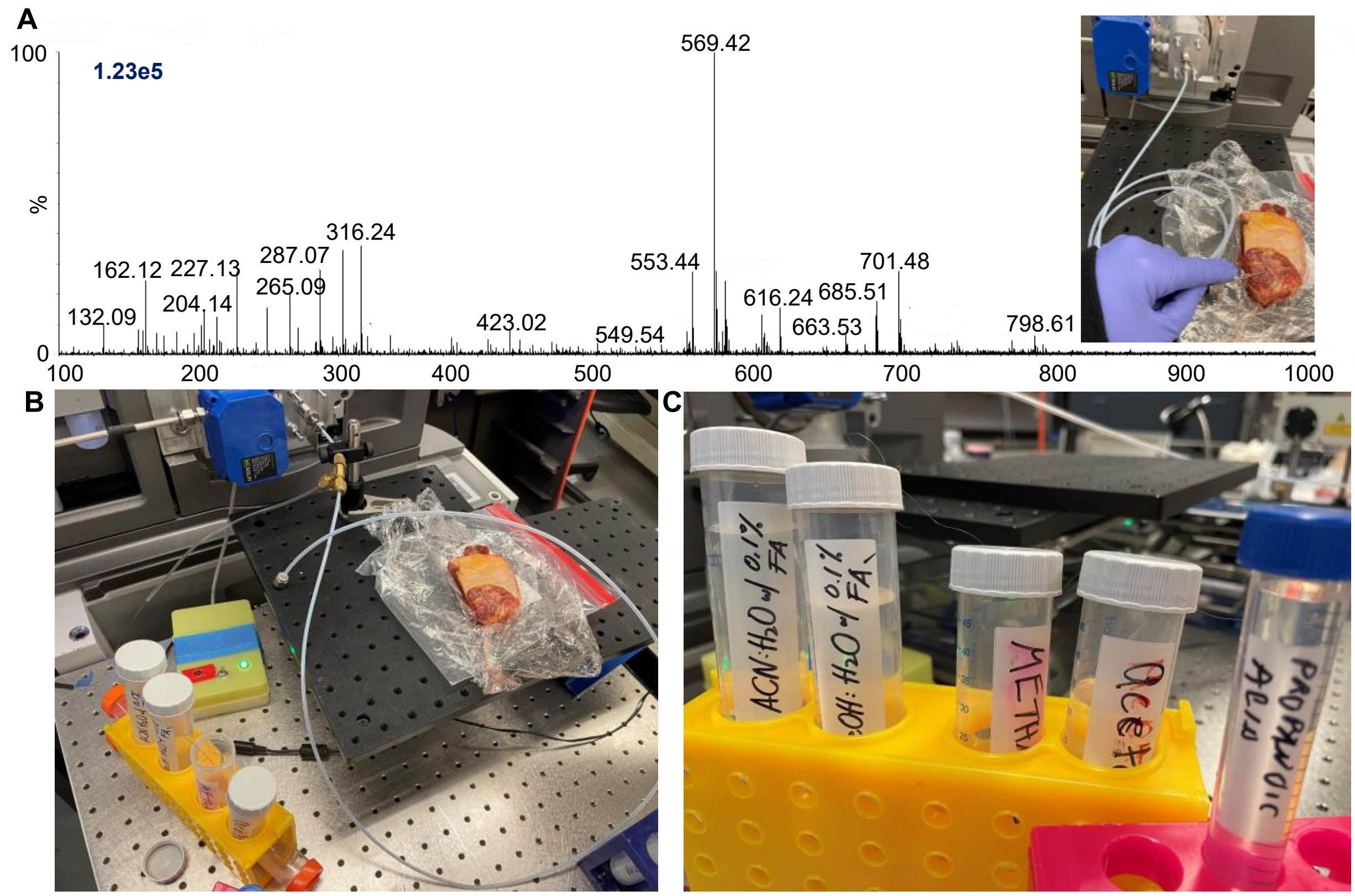

Figure S11: Surface SAI of beef using a 4-port vacuum chamber. (**A**) Methanol placed on a section of beef is transferred into the inlet tube by the pressure differential resulting in the mass spectrum shown. (**B**) Close-up of beef and tubing showing the T-junction[61,62] for solvent mixing, and (**C**) some of the different solvents used to infuse via the T-junction. Data acquired with a Waters SYNAPT G2S mass spectrometer.

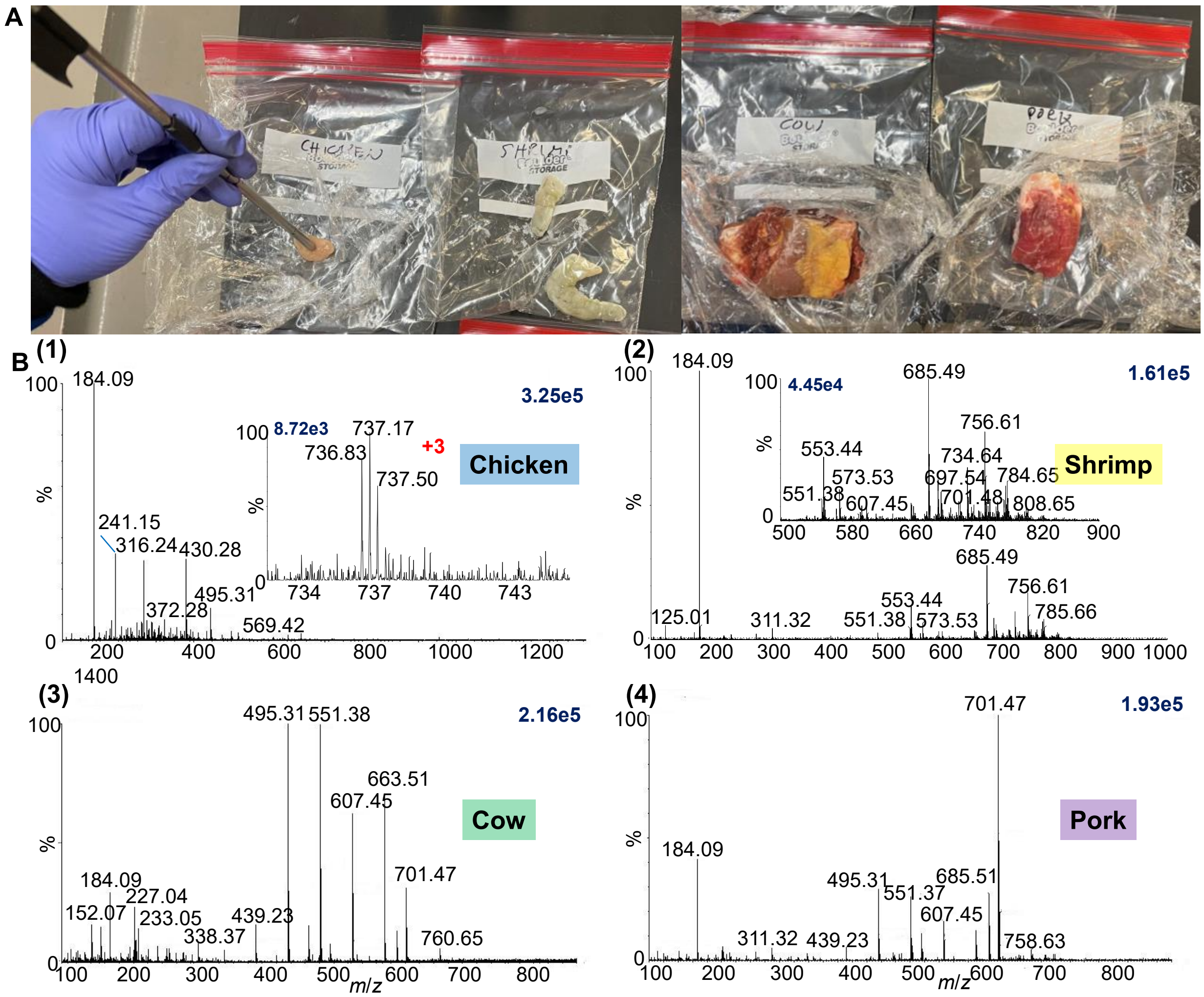

Figure S12: (**A**) Photographs of the meats for vMAI probe data acquisition from different meats (**1**) chicken, (**2**) shrimp, (**3**) cow (beef), and (**4**) pig (pork), (**B**) the mass spectra obtained for the respective food items. The probe tip was touched against each meat and 3-NBN matrix solution added before insertion into the vacuum inside of the 4-port vacuum chamber assembly that was retrofitted on a Waters SYNAPT G2S mass spectrometer.

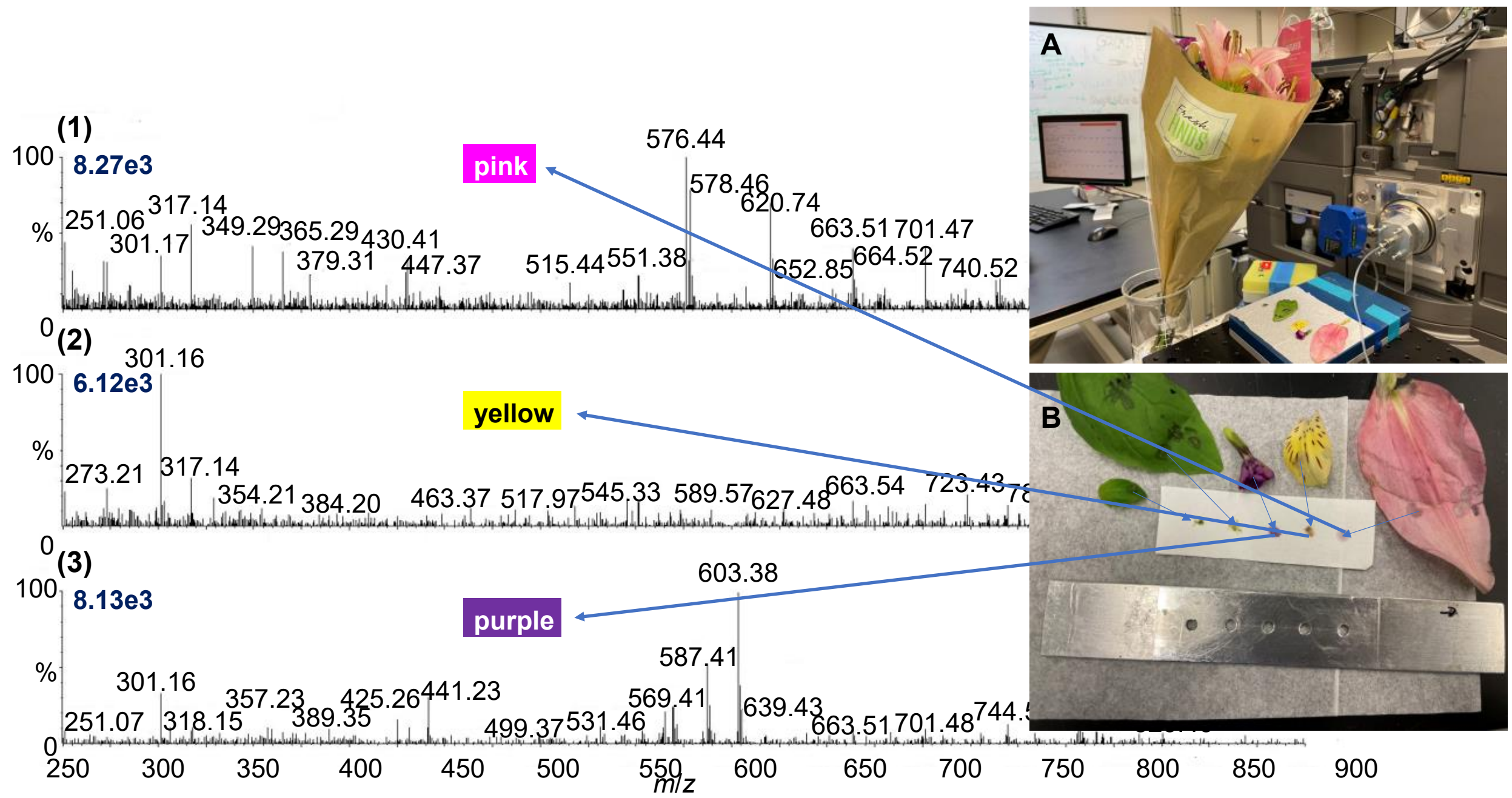

Figure S13: vMAI sampling and analyses of flower petals of different colors. Methanol was added for 30 sec before 1 μL is placed onto filter paper to align with holes in metal spacer plate followed by overlay of 1 μL of 3-NBN matrix solution. Upon brief drying filter paper was inserted into 4-port plate source for rapid vMAI analysis. (**A**) Photographs of hardware technology and (**B**) sampling approach. Mass spectral differences (**1**) to (**3**) are likely related to different flower species rather than color. Photos of the samples provided for illustration purposes. Data acquired on 4-port chamber retrofitted on a Waters SYNAPT G2S.

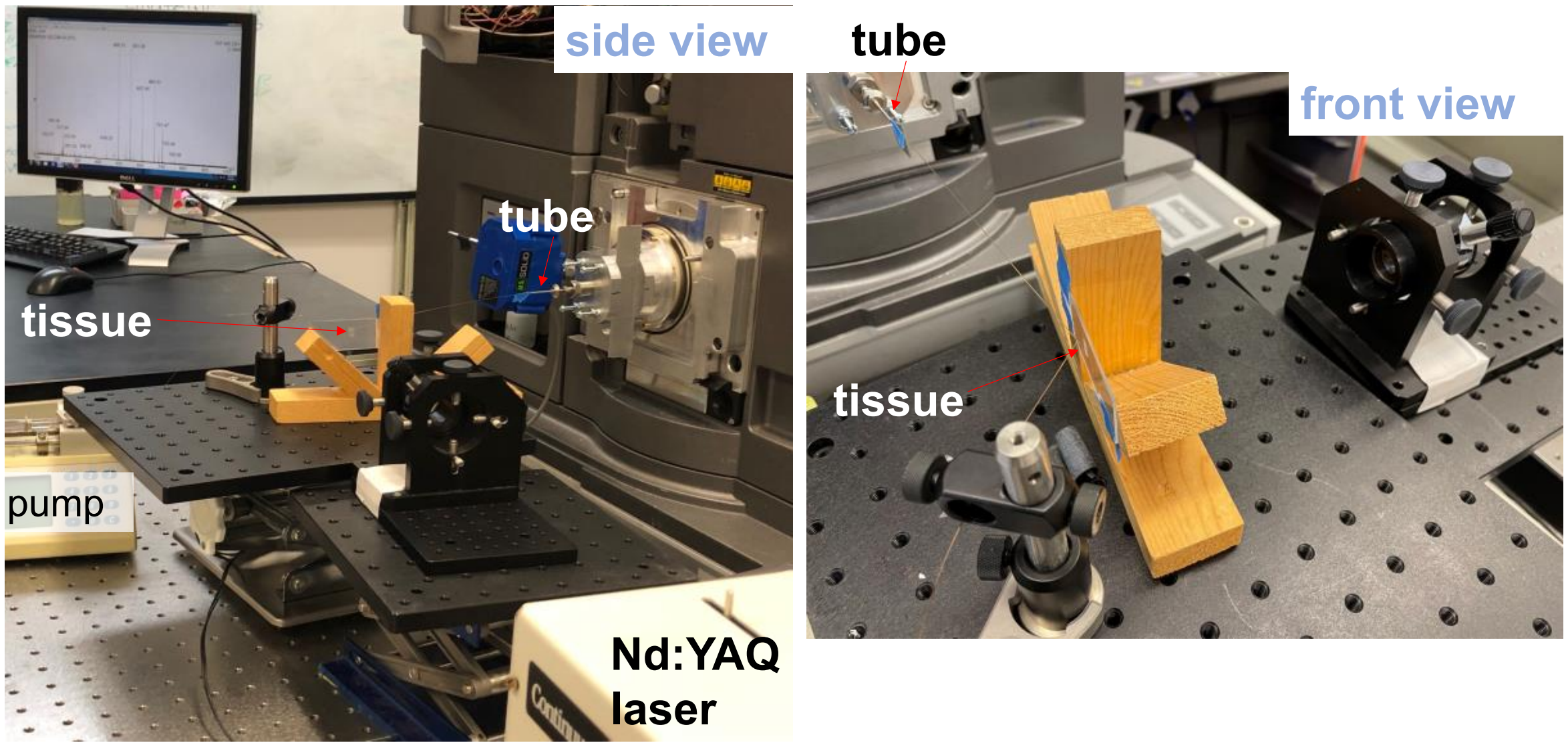

Figure S14: Photographs of hardware technology and surface sampling approach based on laser ablation SAI: capture of ablated material in liquid junction from laser ablation of tissue affixed to glass microscope glass slide. Liquid junction is constantly sampled using fused-silica tubing inserted into the sub-AP of the inlet tube where SAI occurs. Data acquired on 4-port chamber retrofitted on a Waters SYNAPT G2S mass spectrometer.

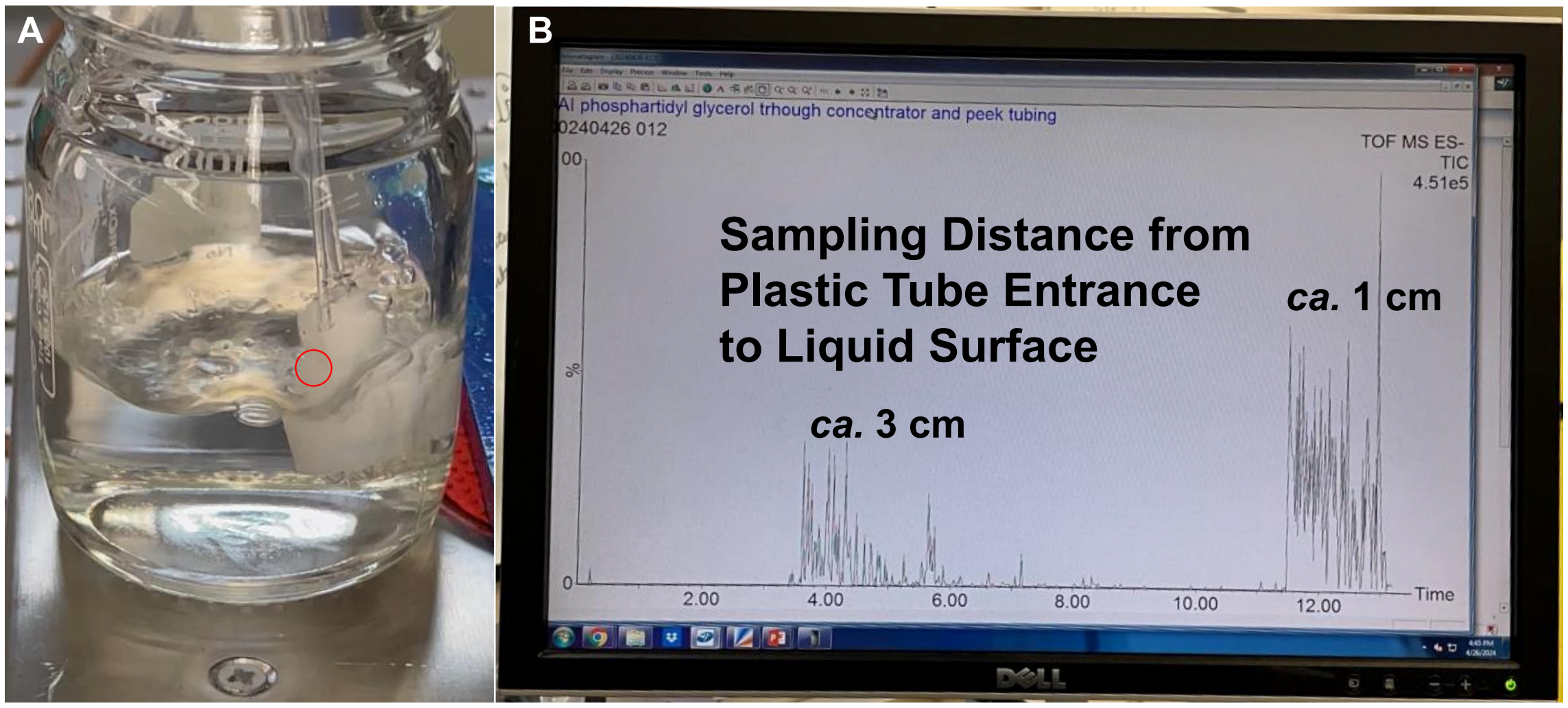

Figure S15: Direct analyses of solutions on the fly containing surface active substances such as per- and polyfluorinated alkyl Substances (PFAS). Perfluorooctanesulfonic acid (PFOS) enrichment using bubble chemistry and SAI-MS. (**A**) The distance of the inlet tube above the solvent surface affects the sampling process, as more droplets are collected when the tube opening is closer to the surface (**B**). Improvements in concentration through bubble chemistry are observed and MS sensitivities increase with the sampling tube closer (~1 cm of the liquid surface) relative to further away (~3 cm), as examples. A gas cylinder was hooked up to provide the bubbles, instead of electrochemical cells; bubble chemistry has been used previously to enrich droplets electrochemical aerosol formation that were collected on a substrate such as a glass plate for several minutes (typically 10 min) before collection and transfer to a mass spectrometer to perform ESI-MS.[63] The solvent is typically drawn into the mass spectrometer within a few seconds giving analytical useful results. In case of the solvent being water, the greater improvements are observed as compared to ESI. Data acquired on 4-port chamber retrofitted on a Waters SYNAPT G2S. In case of water being the solvent, the inlet tube needed to be heated, here, a Variac (typically held around 14 V). In case of other solvents, *e.g.*, methanol, no heat is needed. **Movie S6** is included to display the process of sampling and analyses.

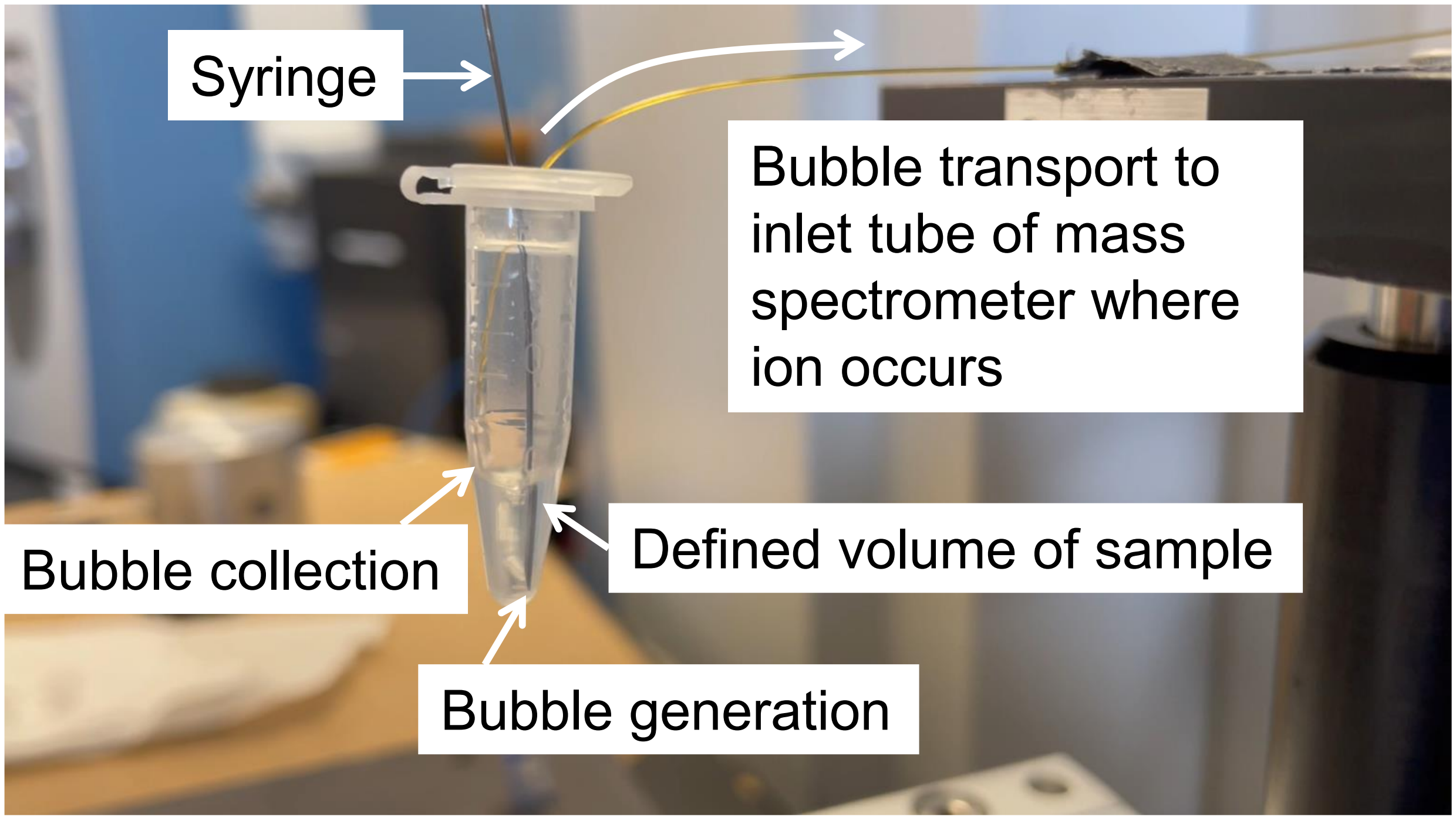

Figure S16: Miniaturized SAI-MS based on bubble chemistry enrichment, similar to the approach presented in **Figure S15**. Data was acquired on a Thermo Q_Exactive Focus. Improvements include (i) bubble generation through blowing air from a syringe into (ii) a specific and small volume of PFOS containing solvent. In case of the solvent being water, largest improvements are observed relative to ESI-MS. Without optimization ~1 ppb was readily detected in full scan MS mode. **Movie S7** is included to display the process of sampling and analyses.

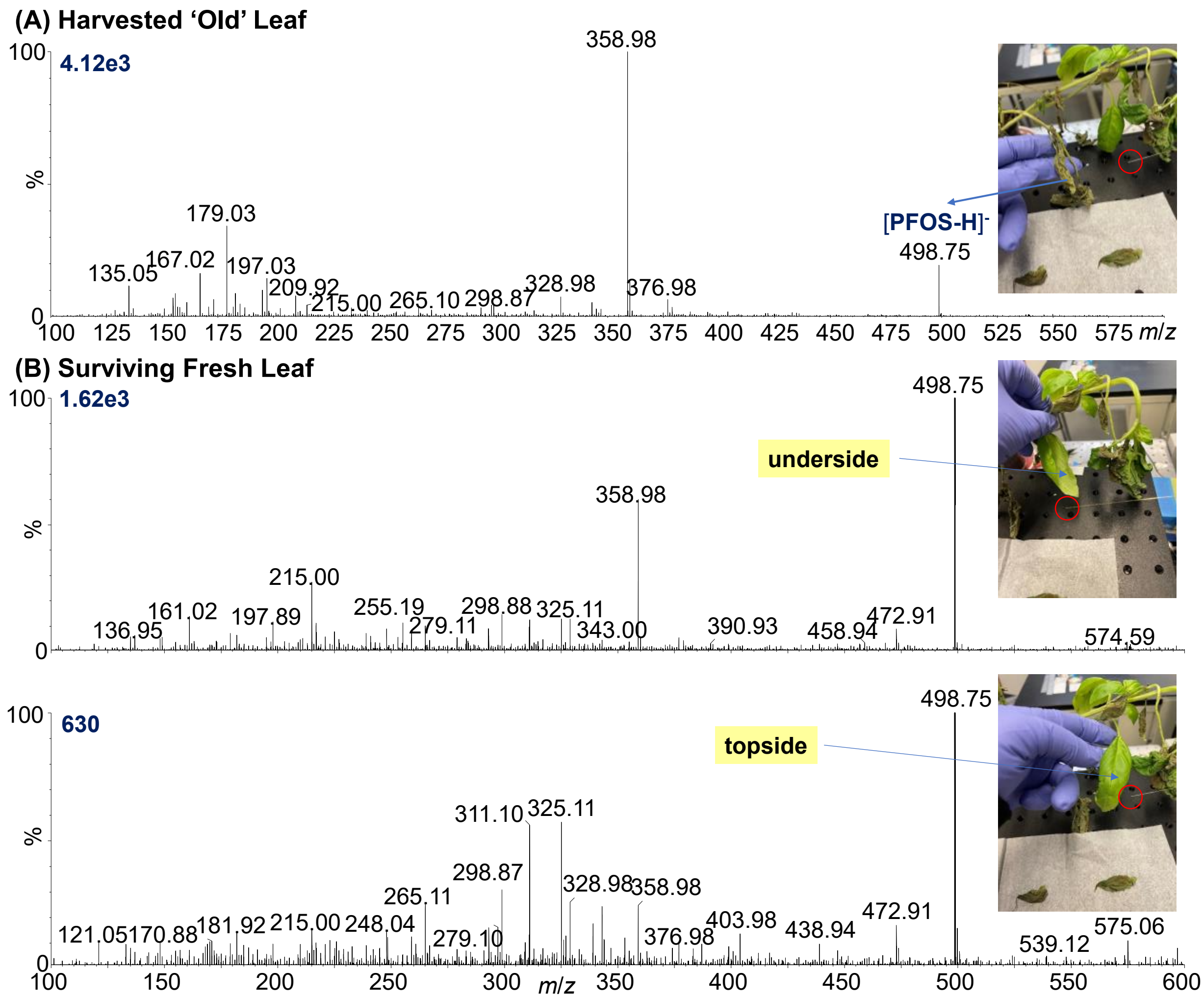

Figure S17: Surface SAI: remote sampling using fused silica tubing inserted into a 4-port inlet tube. (**A**) Sampling of dead leaf 2 days after removal from plant and 7 days after 1 ppm PFOS solution of water was added to growing plant, and (**B**) sampling of living leaf 7 days after PFOS solution was added to living plant. Methanol was added (30 sec) to leaves before being drawn by the mass spectrometer vacuum through the fused-silica tube into the inlet tube. Photos of the samples are provided for illustration purposes. Data was acquired on 4-port vacuum chamber assembly retrofitted on a Waters SYNAPT G2S.

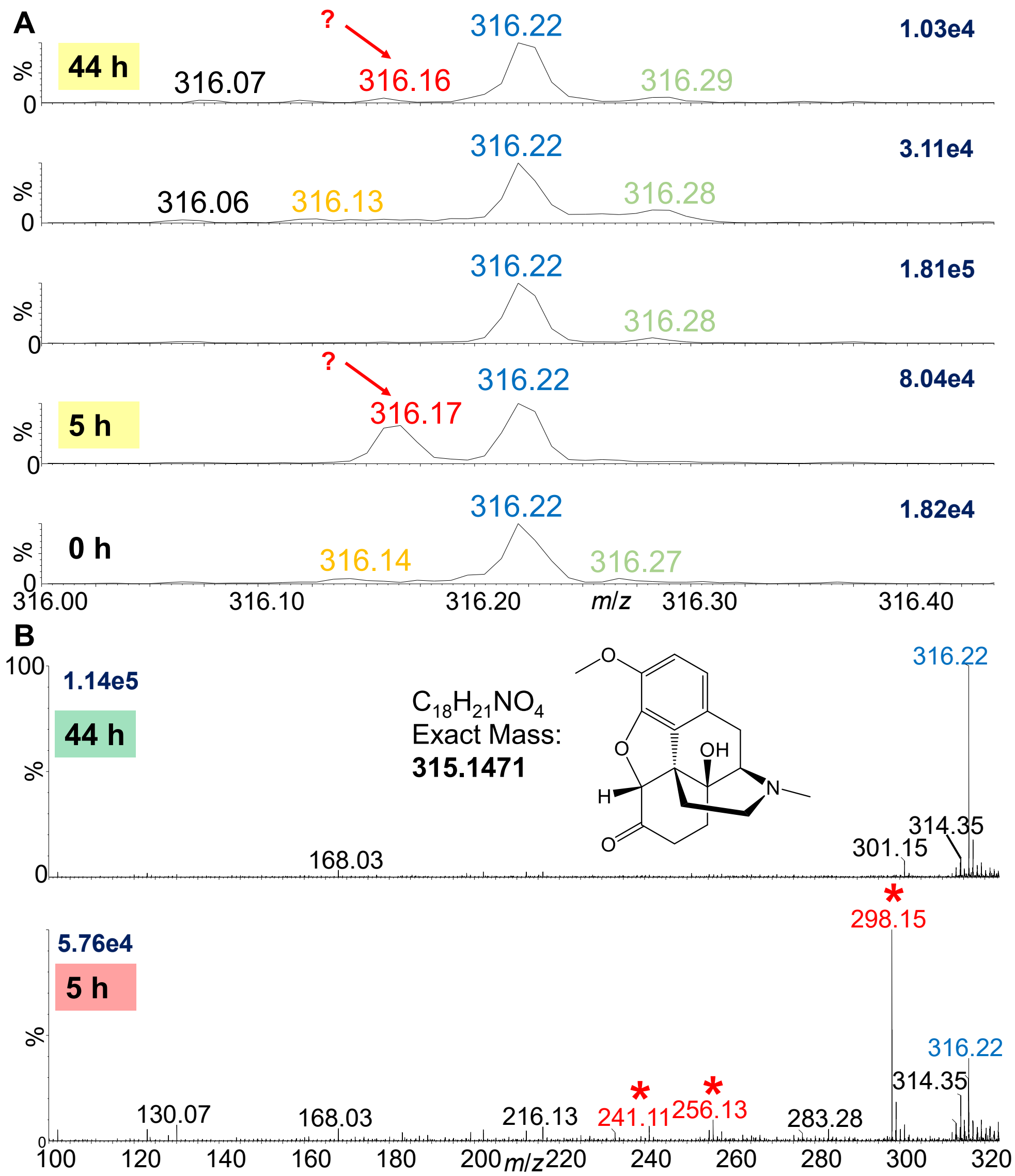

Figure S18: vMAI of oxycodone in urine from a volunteer with back injury taking oxycodone: (**A**) Mass spectra of molecular ion region of oxycodone in urine analysis from 0 – 44 h. time period after taking the medicine, and (**B**) MS/MS fragmentation spectra obtained after 5 h and 44 h. Data was acquired using a 3-port/plate source with filter paper substrate and 3-NBN matrix on a Waters SYNAPT G2S mass spectrometer using an old calibration. MS/MS confirms that the ion displayed at *m*/*z* 316.17 at 5 h is from oxycodone, but not the ion at *m*/*z* 316.16 at 44 h. Oxycodone fragment ions are denoted by *, and are taken from transitions described previously ref. [64].

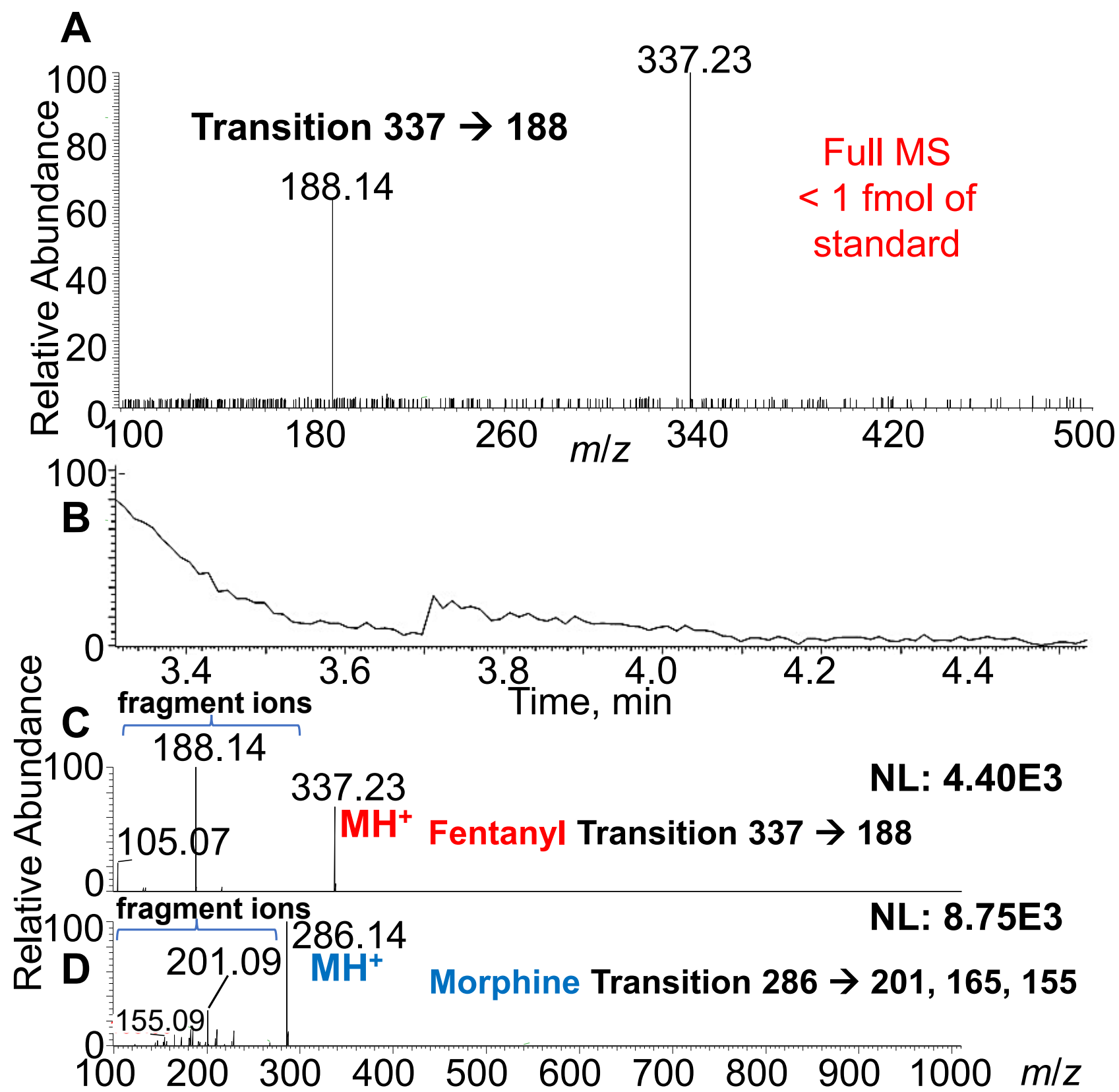

Figure S19: Drugs of concern, fentanyl and morphine, from filter paper using 3-NBN as matrix with analytes applied in sub-picomole amounts. (**A**) Mass spectrum, (**B**) chronogram of ion current, (**C**) MS/MS fragmentation of fentanyl. (**D**) MS/MS fragmentation of morphine. For both illicit drug compounds, the expected fragment ions (compound/ structure specific 'transitions') are observed. Data acquired on 4-port vacuum chamber assembly, using vMAI with filter paper and 3-NBN matrix, retrofitted on a Thermo Q-Exactive Focus mass spectrometer and compared to refs. 65 and 66. **Movies S8** is included to display the process of sampling and analyses.

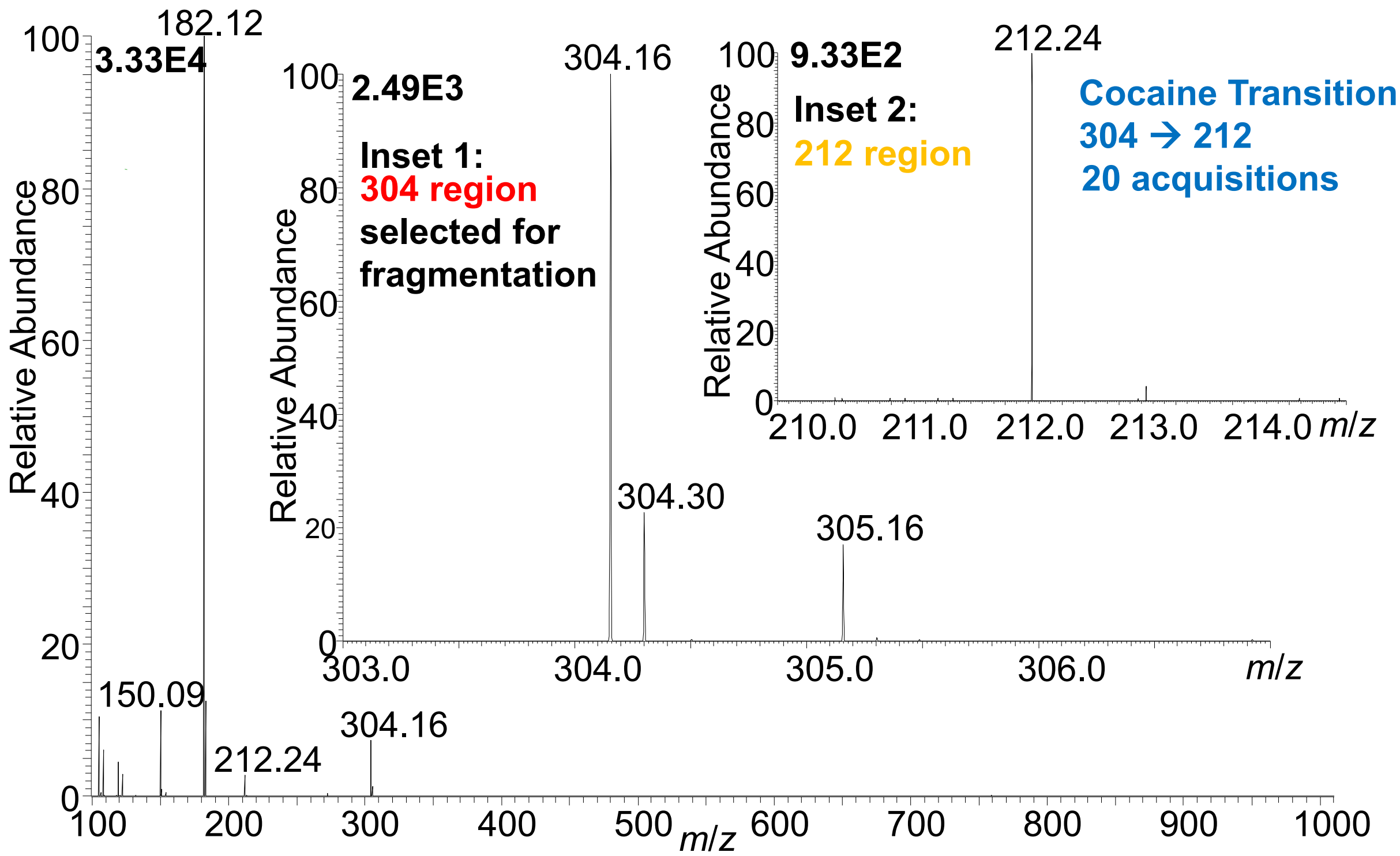

Figure S20. vMAI MS/MS mass spectrum from cocaine spiked in urine and added to filter paper together with a 3-NBN matrix solution. **Inset 1** shows the area of the molecular ion selected for MS/MS and **Inset 2** depicts the area of the distinctive fragment ion at *m*/*z* 212 identifying the presence of cocaine in this complex sample without cleanup. Data acquired on 4-port vacuum chamber assembly retrofitted on a Thermo Q-Exactive Focus instrument. For information on fragment ions and their transitions, refer to ref. 67.

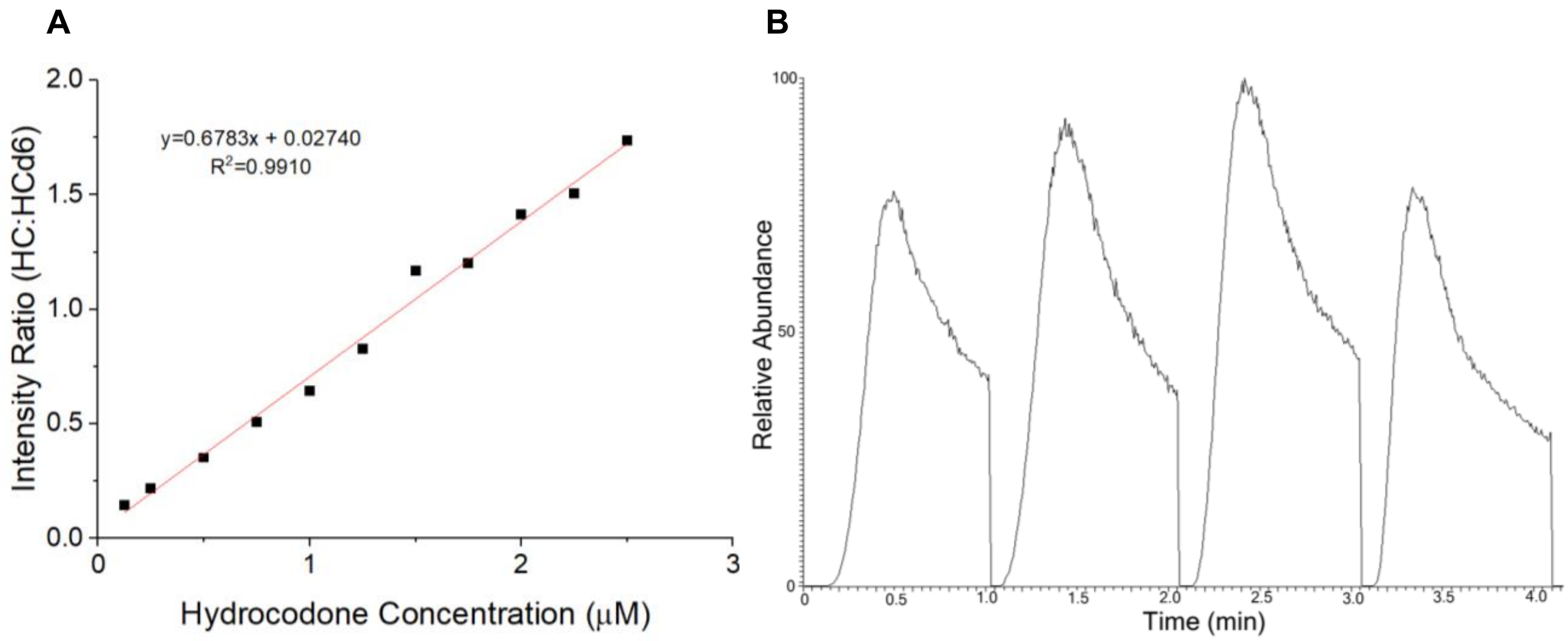

Figure S21. vMAI analyses: (**A**) hydrocodone quantification in a mixture with solutions of hydrocodone ranging from 125 nM to 2.5 μM and (**B**) TIC of the replicate hydrocodone samples. Quantification was accomplished using four replicates at each concentration of hydrocodone with hydrocodone-d6 (1.5 μM) used as an internal standard. 3-NBN matrix solution (1 μL) was mixed into hydrocodone solutions (1 μL) and a microliter of the resulting solution was added to the filter paper. Data was acquired using the 3-port vacuum chamber assembly, equipped with the plate source, retrofitted on a Thermo Q-Exactive Focus mass spectrometer.

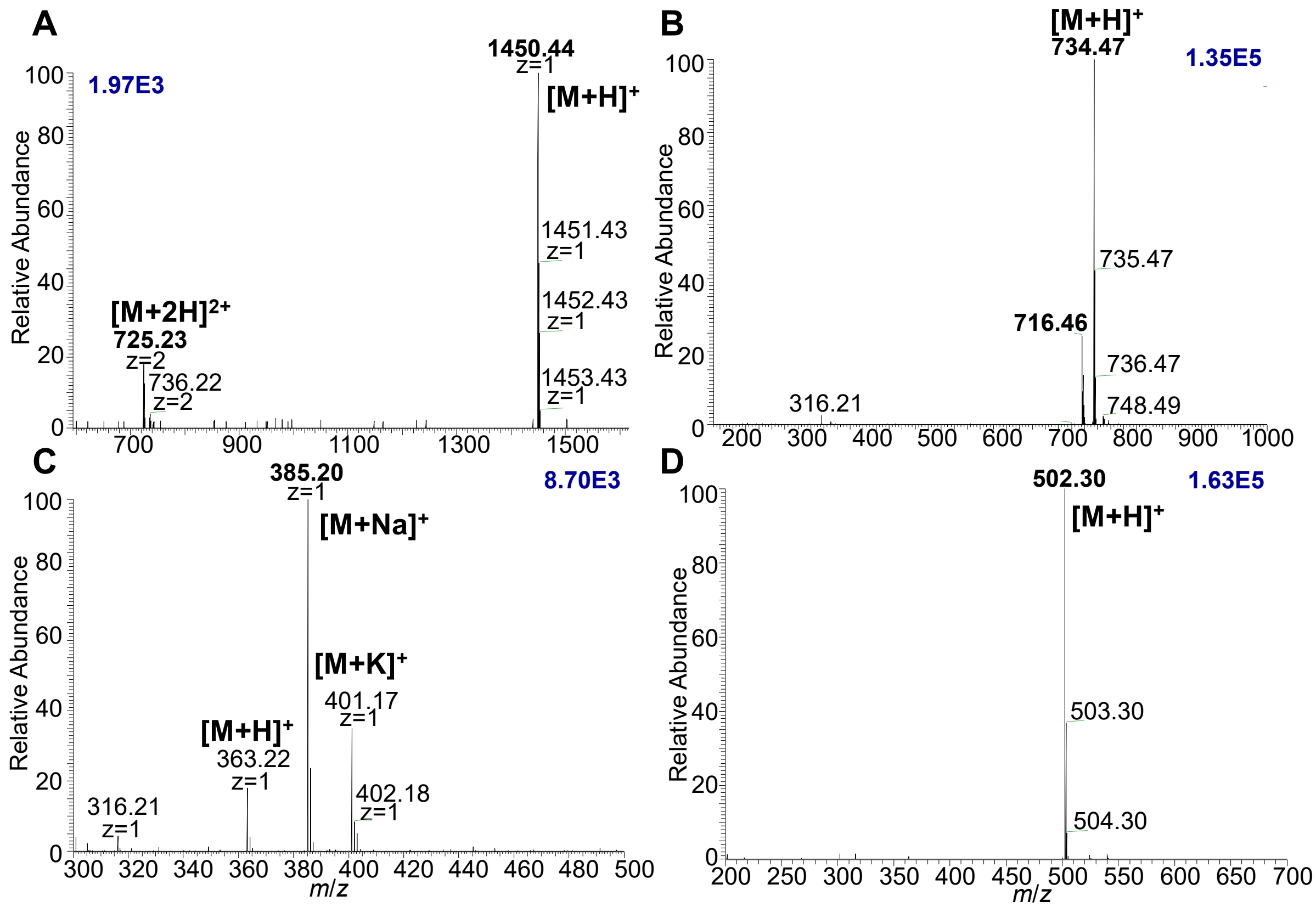

Figure S22: Surface Pen using the principles of SAI to analyze prescription drugs directly from surfaces: remote sampling using peek tubing and connecting it with the inlet tube on a 4-port vacuum chamber assembly. Mass spectra (**A**) vancomycin, (**B**) erythromycin (**C**) hydrocortisone, and (**D**) fexofenadine. Ten microliters of an unknown concentration were deposited on a plastic surface, briefly dried, and solvent was added to re-dissolve the deposit. The tube end was moved close to the liquid surface, so that the mass spectrometer draws the liquid containing the drug into the inlet tube, where ionization occurs. A Variac heater was applied to heat (~100 ºC) the inlet tube for sample ionization, as described in ref. 13. Photographs of the sampling (Variations I and II) are provided for illustration purposes (**Scheme S4**). Data was acquired on 4-port vacuum chamber assembly retrofitted on a Thermo Q-Exactive Focus. Variation III is similar but used in **Scheme S4** on a Waters instrument.

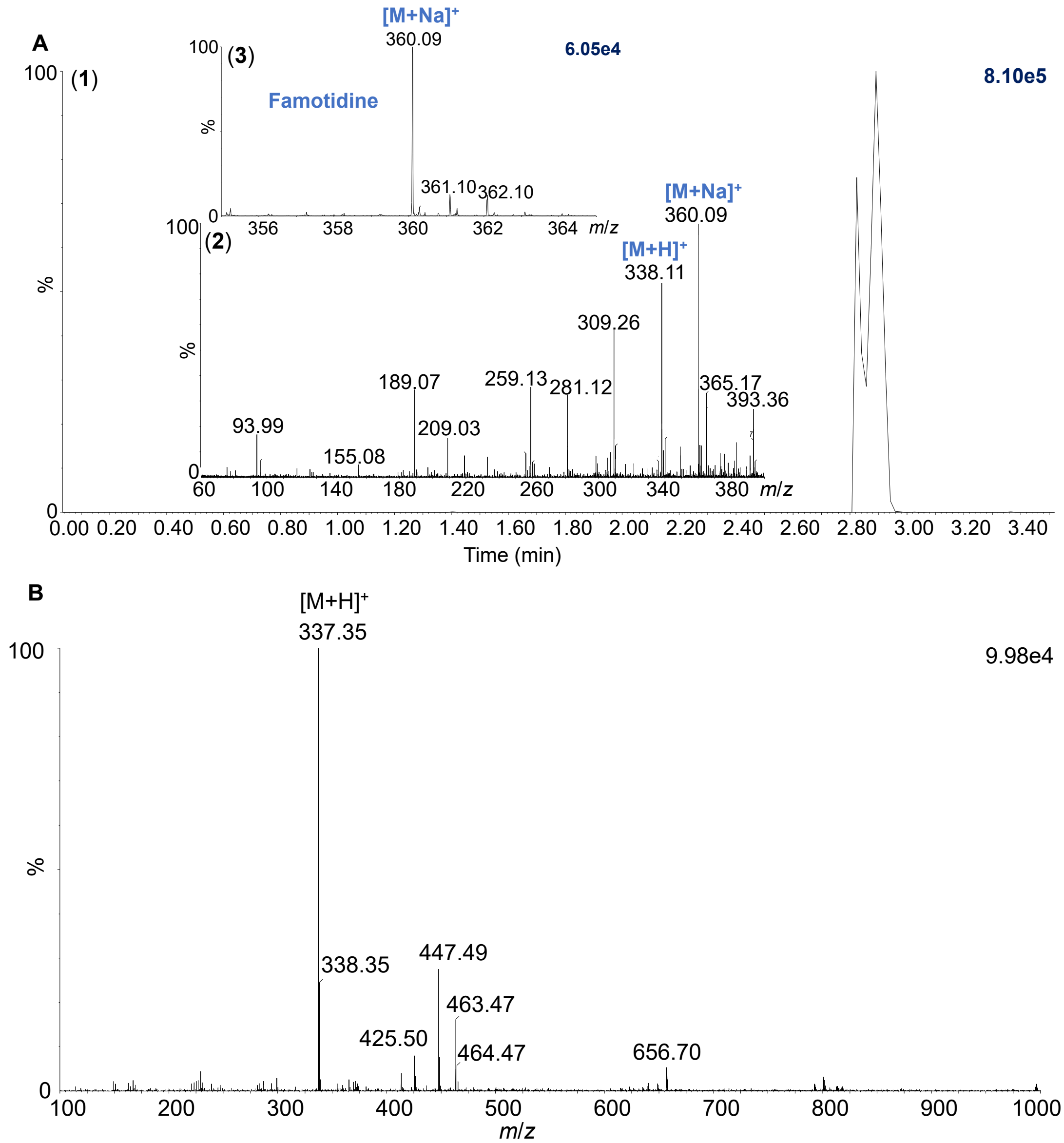

Figure S23: Surface SAI of drugs: (**A**) famotidine, a prescription drug: remote sampling, repurposing FDA-approved tubing and connecting it to the inlet tube on a 4-port vacuum chamber assembly. (**1**) total ion chronogram, mass spectra of famotidine (**2**) full and (**3**) inset. Ten microliters of an unknown concentration were deposited on a metal surface, briefly dried, and methanol was added to re-dissolve the deposit. (**B**) fentanyl, 10 µL of purchased sample was deposited onto a glass plate and solvent (*e.g.*, methanol, water, or mixtures) was added for extraction, pick up, and transport through the second associated tube lining. The tube head was moved over liquid on the surface so that the mass spectrometer draws the liquid containing the drug into the inlet port of the mass spectrometer, where ionization occurs. A Variac heater (14 V) was applied to heat the inlet tube for sample ionization, as described in, *e.g.*, ref. 13. Data were acquired on 4-port vacuum chamber assembly retrofitted on a Waters SYNAPT G2S. Photographs of the sampling (Variation IV) are provided for illustration purposes (**Scheme S4**). **Movies S11** (oxycodone, acquired by a first-year undergraduate student) and **S12** (fentanyl, acquired by a senior expert) are included.

6. Supporting References

1. Trimpin, S.; Yenchick, F. S.; Lee, C.; Hoang, K.; Pophristic, M.; Karki, S.; Marshall, D. D.; Lu, I-C.; Lutomski, C. A.; El-Baba, T. J.; et al. New Processes for Ionizing Nonvolatile Compounds in Mass Spectrometry: The Road of Discovery to Current State-of-the-Art. *J. Am. Soc. Mass Spectrom.* **2024**, *35*, 2753–2784.
2. McEwen, C. N., Larsen, B. S. Fifty Years of Desorption Ionization of Nonvolatile Compounds. *Int. J. Mass Spectrom.* **2015**, *377*, 515–531.
3. Beavis, R. C.; Chaudhary, T.; Chait, B. T. α-Cyano-4-hydroxycinnamic Acid as a Matrix for Matrix-Assisted Laser Desorption Mass Spectrometry. *Organic Mass Spectrometry* **1992**, *27*, 156–158.
4. Trimpin, S.; Inutan, E. D.; Herath, T. N.; McEwen, C. N. Laserspray Ionization, a New Atmospheric Pressure MALDI Method for Producing Highly Charged Gas-phase Ions of Peptides and Proteins Directly from Solid Solutions. *Molecular & Cellular Proteomics* **2010**, *9*, 362–367.
5. McEwen, C. N.; Larsen B. S.; Trimpin S. Laserspray Ionization on a Commercial Atmospheric Pressure-MALDI Mass Spectrometer Ion Source: Selecting Singly or Multiply Charged Ions. *Anal. Chem.* **2010**, *82*, 4998–5001.
6. Trimpin, S.; Ren, Y.; Wang, B., Lietz, C. B.; Richards, A. L.; Marshall, D. D.; Inutan, E. D. Extending the Laserspray Ionization Concept to Produce Highly Charged Ions at High Vacuum on a Time-of-Flight Mass Analyzer. *Anal. Chem.* **2011**, *83*, 5469–5475.
7. Trimpin, S.; Inutan, E. D. Matrix Assisted Ionization in Vacuum, a Sensitive and Widely Applicable Ionization Method for Mass Spectrometry. *J. Am. Soc. Mass Spectrom.* **2013**, *24*, 722–732.
8. Trimpin S.; Lutomski, C. A.; El-Baba, T. J.; Woodall, D. W.; Foley, C. D.; Manly, C. D.; Wang, B.; Liu, C.-W.; Harless, B. M.; Kumar, R.; Imperial, L. F.; Inutan, E. D. Magic Matrices for Ionization in Mass Spectrometry. *Int. J. Mass Spectrom.* **2015**, *377*, 532–545.
9. Inutan, E. D.; Trimpin, S. Matrix Assisted Ionization Vacuum (MAIV), a New Ionization Method for Biological Materials Analysis using Mass Spectrometry. *Molecular & Cellular Proteomics* **2013**, *12*, 792–796.
10. McEwen, C. N.; Inutan, E. D.; Moreno-Pedraza, A.; Lu,I-C.; Hoang, K.; Pophristic, M.; Trimpin S. Sublimation Driven Ionization for Use in Mass Spectrometry: Mechanistic Implications. *J. Am. Soc. Mass Spectrom.* **2021**, *32*, 114–123.
11. Mann, M. The Ever Expanding Scope of Electrospray Mass Spectrometry—a 30 Year Journey. *Nature Communications* **2019**, *10*, 3744.
12. Li, J.; Taraszka, J. A.; Counterman, A. E.; Clemmer, D. E. Influence of Solvent Composition and Capillary Temperature on the Conformations of Electrosprayed Ions: Unfolding of Compact Ubiquitin Conformers from Pseudonative and Denatured Solutions. *Int. J. Mass. Spectrom.* **1999**, *185–187*, 37–47.
13. Pagnotti, V. S.; Chubatyi, N. D.; McEwen, C. N. Solvent Assisted Inlet Ionization: An Ultrasensitive New Liquid Introduction Ionization Method for Mass Spectrometry. *Anal. Chem.* **2011**, *83*, 3981–3985.
14. Pagnotti, V. S.; Inutan, E. D.; Marshall, D. D.; McEwen, C. N.; Trimpin, S. Inlet Ionization: A New Highly Sensitive Approach for Liquid Chromatography/Mass Spectrometry of Small and Large Molecules. *Anal. Chem.* **2011**, *83*, 7591–7594.
15. Wang, B.; Trimpin, S. High-Throughput Solvent Assisted Ionization *Inlet* for Use in Mass Spectrometry. *Anal. Chem.* **2014**, *86*, 1000–1006.
16. Inutan, E. D.; Jarois, D. R.; Lietz, C. B.; El-Baba, T. J.; Elia, E. A.; Karki, S.; Sampat, AAS; Foley, C. D.; Clemmer, D. E.; Trimpin, S. Comparison of Gaseous Ubiquitin Ion Structures Obtained from a Solid and Solution Matrix using Ion Mobility Spectrometry/Mass Spectrometry. *Rapid Commun. Mass Spectrom.* **2021**, *35*, e8793.
17. McEwen, C. N. Pagnotti, V. S.; Inutan, E. D.; Trimpin, S. New Paradigm in Ionization: Multiply Charged Ion Formation from a Solid Matrix Without a Laser or Voltage. *Anal. Chem.* **2010**, *82*, 9164–9168.

18. Pagnotti, V. S.; Chakrabarty, S.; Wang, B.; Trimpin, S.; McEwen, C. N. Gas-phase Ions Produced by Freezing Water or Methanol for Analysis using Mass Spectrometry. *Anal. Chem.* **2014**, *86*, 7343–7350.
19. Inutan, E. D.; Wang, B.; Trimpin, S. Commercial Intermediate Pressure MALDI Ion Mobility Spectrometry Mass Spectrometer Capable of Producing Highly Charged Laserspray Ionization Ions. Anal. Chem. **2011**, *83*, 678–684.
20. McPartlan, M. S.; Chen, C. J.; Harper, C. C.; Miller, Z. M.; Robles, J.; Avadhani, V. S.; Pedder, R. E.; Metzler, L. J.; Williams, E. R. Atmospheric Sampling Mass Spectrometers Activate and Ionize Neutral Water Microdroplets to MeV Energies and up to 200,000+ Charges: Implications for Water Stability and Unusual Chemistry in Microdroplets. *ACS Central Science* Article ASAP, DOI: 10.1021/acscentsci.5c01518
21. Harrison, J.; Risby, K. M.; Miles, B. E. A.; Hilditch, T. G.; Walker, J. S.; Bzdek, B. R. The Role of Aerosol Liquid Water in Droplet-Assisted Ionization Mass Spectrometry. *Anal. Chem.* **2025**, *97*, 19918–19925.
22. Walker, J. S.; Bzdek, B. R. Rapid and Sensitive Chemical Analysis of Individual Picolitre Droplets by Mass Spectrometry. *Anal. Chem*. **2025**, *97*, 854–861.
23. Consta, S.; Wingen, L. M.; Qin, Y.; Perraud, V.; Finlayson-Pitts, B. J. Modeling the Ionization Mechanism of Amorphous Solid Particles Without an External Energy Source Coupled to Mass Spectrometry. *Phys. Chem. Chem. Phys.* **2024**, *26*, 28220–28233.
24. Qin, Y.; Perraud, V.; Finlayson-Pitts, B. J.; Wingen, L. M. Peroxides on the Surface of Organic Aerosol Particles Using Matrix-Assisted Ionization in Vacuum (MAIV) Mass Spectrometry. *Environ. Sci. Technol*. **2023**, *57*, 14260–14268.
25. Qin, Y.; Wingen, L. M.; Finlayson-Pitts, B. J. Toward a Molecular Understanding of the Surface Composition of Atmospherically Relevant Organic Particles. *Proc. Natl. Acad. Sci. U.S.A.* **2022**, *119*, e2209134119.
26. Limbach, M. N.; Do, T. D. Solvent-Free Nuclear Magnetic Resonance Spectroscopy of Charged Molecules. *J. Phys. Chem. A* **2023**, *127*, 9149–9157.
27. Hofmeister, A. M. Linear Dependence of Sublimation Enthalpy on Young's Elastic Modulus: Implications for Thermodynamics of Solids. *Materials* **2025**, *18*, 3535.
28. Bertrand, E.; Gabelica, V. Thermometer Ions, Internal Energies, and In-Source Fragmentation in Ambient Ionization. *Mass Spectrom. Rev*. **2026**, *45*, 260–281.
29. Smith, B. L.; Hankinson, T.; Maher, S. Portable Instrumentation for Ambient Ionization and Miniature Mass Spectrometers. *Annual Rev. Anal. Chem*. **2024**, *17*, 69–102.
30. Zhai, Y.; Fu, X.; Xu, W. Miniature Mass Spectrometers and Their Potential for Clinical Point-of-Care Analysis. *Mass Spectrom. Rev*. **2024**, *43*, 1172–1191.
31. Zhang, J.; Rector, J.; Lin, J. Q.; Young, J. H.; Sans, M.; Katta, N.; Giese, N.; Yu, W.; Nagi, C.; Suliburk, J.; *et al.* Nondestructive Tissue Analysis for Ex Vivo and In Vivo Cancer Diagnosis using a Handheld Mass Spectrometry System. *Sci Transl Med.* **2017**, *9*, eaan3968.
32. Chen, M.; Huang, Q.; Li, Y.; Li, B.; Zhang, Z.; Zhang, L.; Li, C.; Ma, Q. Ambient Ionization Mass Spectrometry for Rapid Detection of Biological Warfare Agents and Their Simulants. *Microchemical J*. **2023**, *194*, 109275.
33. Guo, X.; Shang, Y.; Lv, Y.; Bai, H.; Ma, Q. Suspect Screening of Fentanyl Analogs using Matrix-Assisted Ionization and a Miniature Mass Spectrometer with a Custom Expandable Mass Spectral Library. *Anal. Chem*. **2021**, *93*, 10152–10159.
34. Jiang, S.; Apsokardu, M. J.; Liu, Y. R.; Wang, C. Y.; Huang, T.; Johnston, M. V. Ion Formation Mechanism of Cortisone Molecules and Clusters in Charged Nanodroplets. *Phys Chem Chem Phys* **2022**, *24*, 28047–28054.
35. Apsokardu, M. J.; Krasnomowitz, J. M.; Jiang, S.; Johnston, M. V. Ion Formation from Rapidly Heated Aqueous Droplets by Droplet-Assisted Ionization. *J. Phys. Chem. A* **2020**, *124*, 7313–7321.
36. Horan, A. J.; Apsokardu, M. J.; Johnston, M. V. Droplet Assisted Inlet Ionization for Online Analysis of Aairborne Nanoparticles. *Anal. Chem.* **2017**, *89*, 1059–1062.
37. Mannion, D. R.; Mannion, J. M.; Kuhne, W. W.; Wellons, M. Matrix-Assisted Ionization of Molecular Uranium Species. *J. Am. Soc. Mass Spectrom.* **2021**, 32, 8–13.

38. Meher, A. K.; Green, Z. A. Analytical Chemistry—Recent Innovations. *Analytica* **2025**, *6*, 10.
39. Chakrabarty, S.; Shelver, W. L.; Smith, D. J. Electrospray Ionization Inlet Tandem Mass Spectrometry: A Hyphenated Method for the Sensitive Determination of Chemicals in Animal Tissues and Body Fluids. *J. Am. Soc. Mass Spectrom.* **2021**, *32*, 14–20.
40. Halvorsen, T. G.; Levernæs, M. C. S.; Rosting, C. Matrix-Assisted Ionization and Tandem Mass Spectrometry Capabilities in Protein Biomarker Characterization—An Initial Study Using the Small Cell Lung Cancer Biomarker Progastrin Releasing Peptide as a Model Compound. *J. Am. Soc. Mass Spectrom.* **2021**, *32*, 611–614.
41. Trimpin, S. A Tutorial: Laserspray Ionization and Related Laser-Based Ionization Methods for Use in Mass Spectrometry. *Mass Spectrom. Rev.* **2023**, *42*, 2234–2267.
42. Trimpin, S.; Inutan, E. D.; Pagnotti, V. S.; Karki, S.; Marshall, D. D.; Hoang, K.; Wang, B.; Lietz, C. B.; Richards, A. L.; Yenchick, F. S.; Lee, C.; Lu, I-C.; Fenner, M.; Madarshahian, S.; Saylor, S.; Chubatyi, N. D.; Zimmerman, T.; Moreno-Pedraza, A.; Wang, T.; Adeniji-Adele, A.; Meher, A. K.; Madagedara, H.; Owczarzak, Z.; Musavi, A.; Hendrickson, T. L.; Peacock, P. M.; Tomsho, J. W.; Larsen, B. S.; Prokai, L.; Shulaev, V.; Pophristic, M.; McEwen, C. N. Direct Sub-Atmospheric Pressure Ionization Mass Spectrometry: *Evaporation/Sublimation*-Driven Ionization is Amazing, Fundamentally, and Practically. *J. Mass Spectrom.* **2024**, *59*, e5018.
43. Hoang K.; Trimpin S.; McEwen C. N.; Pophristic M. A Combination MAI and MALDI Vacuum Source Operational from Atmospheric Pressure for Fast, Robust, and Sensitive Analyses. *J. Am. Soc. Mass Spectrom.* **2021**, *32*, 124–132.
44. Trimpin S.; Inutan E.; Coffinberger H.; Hoang K.; Yenchick F.; Wager-Miller J.; Pophristic M.; Mackie K.; McEwen C. N. Instrumentation Development, Improvement, Simplification, and Miniaturization: The Multifunctional Plate Source for Use in Mass Spectrometry. *J. Eur. J. Mass Spectrom.* **2023**, *29*, 276–291.
45. Chen, L. C.; Rahman, M. M.; Hiraoka, K. Super-Atmospheric Pressure Ion Sources: Application and Coupling to API Mass Spectrometer. *Mass Spectrom (Tokyo)*. **2014**, *3(Spec Iss)*:S0024.
46. Das, S.; Bhatia, R. Liquid Extraction Surface Analysis-Mass Spectrometry: An Advanced and Environment-Friendly Analytical Tool in Modern Analysis. *Anal. Science J.* **2022**, *45*, 2746–2765.
47. Lin, M.; Blevins, M. S.; Sans, M.; Brodbelt, J. S.; Eberlin, L. S. Deeper Understanding of Solvent-Based Ambient Ionization Mass Spectrometry: Are Molecular Profiles Primarily Dictated by Extraction Mechanisms? *Anal. Chem.* **2022**, *94*, 14734–14744.
48. Takáts, Z.; Wiseman, J. M.; Gologan, B.; Cooks, R. G. Mass Spectrometry Sampling Under Ambient Conditions with Desorption Electrospray Ionization. *Science* **2004**, *306*, 471–473.
49. Van Berkel, G. J.; Kertesz, V. Application of a Liquid Extraction Based Sealing Surface Sampling Probe for Mass Spectrometric Analysis of Dried Blood Spots and Mouse Whole-body Thin Tissue Sections. *Anal. Chem.* **2009**, *81*, 9146–9152.
50. Pagnotti, V. S.; Chakrabarty, S.; Harron, A. F.; McEwen, C. N. Increasing the Sensitivity of Liquid Introduction Mass Spectrometry by Combining Electrospray Ionization and Solvent Assisted Inlet Ionization. *Anal. Chem.* **2012**, *84*, 6828–6832.
51. Trimpin, S.; Rauschenbach, S.; Kern, K.; Lu, I-C.; Inutan, E. D.; Fischer, J. L.; Thawoos, S.; Devereux, Z. J.; Zhang, W.-J.; Zhang, W.; Dillenburger, M.; Raeder, H.-J.; Muellen, K.; Hoang,K.; Sidorenko, A.; Pophristic, M.; McEwen, C. N. Fundamental Studies of Inlet Ionization with Sample Introduction at Atmospheric Pressure. ASMS 2016, San Antonio, TX, ThOD pm 02:50; 283128.
52. Pagnotti, V., McEwen, C. N. Inlet Ionization: An Investigation into the Mechanism That Produces Ions from an Instrument-Inlet Absent of Lasers, Heat or Voltages. ASMS 2011, WOE am 09:50; 263680.
53. Trimpin, S.; *et al.* Direct Sub-Atmospheric Pressure Ionization Mass Spectrometry: Evaporation/Sublimation-Driven Ionization is Amazing, Fundamentally, and Practically. *J. Mass Spectrom.* **2024**, *59*, e5018.
54. Trimpin, S. "Magic" Ionization Mass Spectrometry. *J. Am. Soc. Mass Spectrom.* **2016**, *27*, 4–21.
55. Lu, I-C.; Elia, E. A.; Zhang, W.-J.; Pophristic, M.; Inutan, E. D.; McEwen, C. N.; Trimpin, S. Development of an Easily Adaptable, High Sensitivity Source for Inlet Ionization. *Anal. Methods* **2017**, *9*, 4971–4978.

56. Cao, Y.; Lee, C.; Davis, E. T. J.; Si, W.; Wang, F.; Trimpin, S.; Luo, L. 1000-Fold Preconcentration of Per-and Polyfluorinated Alkyl Substances Within 10 Minutes via Electrochemical Aerosol Formation. *Anal. Chem.* **2019**, *91*, 14352–14358.
57. Trimpin, S.; Marshall, D. D.; Karki, S.; Madarshahian, S.; Hoang, K.; Meher, A. K.; Pophristic, M.; Richards, A. L.; Lietz, C. B.; Fischer, J. L.; Elia, E. A.; Wang, B.; Pagnotti, V. S.; Lutomski, C. A.; El-Baba, T. J.; Lu, I-C.; Wager-Miller, J.; Mackie, K.; McEwen, C. N.; Inutan, E. D. An Overview of Biological Applications and Fundamentals of New Inlet and Vacuum Ionization Technologies. *Rapid Commun. Mass Spectrom.* **2021**, *35*, e8829.
58. Pagnotti, V. S.; Inutan, E. D.; Marshall, D. D.; McEwen, C. N.; Trimpin, S. Inlet Ionization: A New Highly Sensitive Approach for Liquid Chromatography/Mass Spectrometry of Small and Large Molecules. *Anal. Chem.* **2011**, *83*, 7591–7594.
59. Inutan, E. D.; Jarois, D. R.; Lietz, C. B.; El-Baba, T. J.; Elia, E. A.; Karki, S.; Sampat, AAS; Foley, C. D.; Clemmer, D. E.; Trimpin, S. Comparison of Gaseous Ubiquitin Ion Structures Obtained from a Solid and Solution Matrix using Ion Mobility Spectrometry/Mass Spectrometry. *Rapid Commun. Mass Spectrom.* **2021**, *35*, e8793.
60. Wang, B.; Dearring, C. L.; Wager-Miller, J.; Mackie, K.; Trimpin, S. Drug Detection and Quantification Directly from Tissue using Novel Ionization Methods for Mass Spectrometry. *Eur. J. Mass Spectrom.* **2015**, *21*, 201–210.
61. Trimpin, S.; Wang, B. Book Chapter, Chapter 17: Inlet and Vacuum Ionization from Ambient Conditions, in Series: New Developments in Mass Spectrometry, Ambient Ionization Mass Spectrometry. Royal Society of Mass Spectrometry. **2014**. https://doi.org/10.1039/9781782628026-00423
62. Marshall, D. D.; Inutan, E. D.; Wang, B.; Liu, C.-W.; Thawoos, S.; Wager-Miller, J.; Mackie, K.; Trimpin, S. A Broad-based Study on Hyphenating New Ionization Technologies with MS/MS for PTMs and Tissue Characterization. *J. Proteomics* **2016**, *16*, 1695–1706.
63. Cao, Y.; Lee, C.; Davis, E. T. J.; Si, W.; Wang, F.; Trimpin, S.; Luo, L. 1000-Fold Preconcentration of Per- and Polyfluorinated Alkyl Substances within 10 Minutes via Electrochemical Aerosol Formation. *Anal. Chem.* **2019**, *91*, 14352–14358.
64. Pennanen, K.; Kotiaho, T.; Huikko K.; Kostiainen, R. Identification of Ozone Oxidation Products of Oxycodone by Electrospray Ion Trap Mass Spectrometry. *J. Mass Spdectrom.* **2001**, *36*,791–797.
65. Strayer, K. E.; Antonides, H. M.; Juhascik, M. P.; Daniulaityte, R.; Sizemore, I. E. LC-MS/MS-Based Method for the Multiplex Detection of 24 Fentanyl Analogues and Metabolites in Whole Blood at Sub ng mL^{-1} Concentrations. ACS Omega **2018**, *3*, 514–523.
66. Zhang, Z.; Yan, B.; Liu, K.; Bo, T.; Liao, Y.; Liu, H. Fragmentation Pathways of Heroin-Related Alkaloids Revealed by Ion Trap and Quadrupole Time-of-Flight Tandem Mass Spectrometry. *Rapid Commun. Mass Spectrom.* **2008**, *22*, 2851–2862.
67. Bailey, M. J.; Bradshaw, R.; Francese, S.; Salter, T. L.; Costa, C.; Ismail, M.; Webb, R. P.; Bosman, I.; Wolff, K.; de Puit, M. Rapid Detection of Cocaine, Benzoylecgonine and Methylecgonine in Fingerprints using Surface Mass Spectrometry. *Analyst* **2015**, *140*, 6254–6259.
